# Visible-light-induced bromoetherification of alkenols for the synthesis of β-bromotetrahydrofurans and -tetrahydropyrans

**DOI:** 10.3762/bjoc.11.5

**Published:** 2015-01-08

**Authors:** Run Lin, Hongnan Sun, Chao Yang, Youdong Yang, Xinxin Zhao, Wujiong Xia

**Affiliations:** 1 State Key Lab of Urban Water Resource and Environment, the Academy of Fundamental and Interdisciplinary Sciences, Harbin Institute of Technology, Harbin 150080, P. R. China

**Keywords:** alkenols, bromoetherification, photoredox catalysis, visible light

## Abstract

A visible-light-induced photoredox-catalyzed bromoetherification of alkenols is described. This approach, with CBr_4_ as the bromine source through generation of bromine in situ, provides a mild and operationally simple access to the synthesis of β-bromotetrahydrofurans and -tetrahydropyrans with high efficiency and regioselectivity.

## Introduction

The halocyclization of alkenes provides an excellent synthetic method for halogenated heterocycles [1–3]. In recent years, haloaminocyclization [4–5], halolactonization [6–7] and haloetherification [8–9] of alkenes have received considerable attention from chemists, and various approaches have been made in this area. Initially, the classical synthetic pathway for bromocyclization proceeds utilizing bromine [10]. However, molecular bromine is hazardous and difficult to handle. Further research show that *N*-bromosuccinimide (NBS) is an effective alternative for the bromocyclization [11–14]. Furthermore, Wei Sun and co-workers disclosed an intriguing strategy to access the haloetherfication of alkenols with *N*-chlorosuccinimide (NCS), leading to the synthesis of β-chlorotetrahydrofurans [15]. Recently we have reported that visible-light-induced photoredox catalysis could serve as a more environmental-friendly alternative reaction system to obtain Br_2_ in situ from CBr_4_, an oxidative quencher of photoredox catalyst [16–22]. Thus, as part of difunctionalization of alkenes, with our continuous investigations on the photoredox catalytic reactions [16,23–27], herein we report our preliminary studies on visible-light-induced photoredox-catalyzed bromoetherification of alkenols using CBr_4_ as the bromine source.

## Results and Discussion

Our initial studies were focused on the reaction of alkenol **1a** as a model reaction for optimizing the reaction conditions. We were encouraged by the discovery that when **1a**, CBr_4_ and Ru(bpy)_3_Cl_2_ were irradiated by blue LEDs in MeCN for 4 hours, *trans*-β-bromotetrahydrofuran **2a** was obtained via 5-*endo* bromoetherification reaction, although the yield was only 31% (Table 1, entry 2). We have reported the bromoetherification of compound **1a** as an example in our previous article [16]. However, considering the value of this strategy for the synthesis of β-bromotetrahydrofurans and -tetrahydropyrans, further research were carried out to optimize the reaction conditions. Moreover, the stereochemistry of the bromotetrahydrofurans compound **2a** was misidentified before. Herein, the stereochemistry of the bromotetrahydrofurans compound **2a** was determined by NOE spectra, for details see Supporting Information File 1. After a screening of selected solvents, we found solvents had a significant effect on the reaction efficiency (Table 1, entries 1–5). The reaction in DMSO led to the highest yield up to 94% (Table 1, entry 1). In addition, 2 equivalents of CBr_4_ were required for the efficient transformation (Table 1, entries 6 and 7). Furthermore, when the catalyst loading was reduced to even 1 mol %, the reaction also gave a comparable result (Table 1, entry 8). It should be pointed out that no reaction was observed in the absence of light or photocatalyst.

**Table 1:** Survey on the photocatalytic bromoetherification of alkenols.

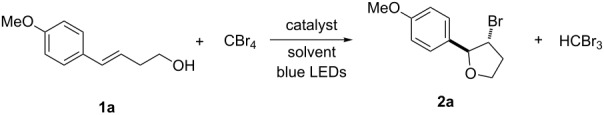			

Entry	Conditions	Time (h)	Yield (%)^b^

1	Standard conditions^a^	4	94
2	CH_3_CN as solvent	4	31
3	DCM as solvent	12	28
4	THF as solvent	24	71
5	DMF as solvent	22	77
6	Only 1 equiv CBr_4_ was used	6	76
7	Only 1.5 equiv CBr_4_ was used	5	88
8	Only 1 mol % Ru(bpy)_3_Cl_2_ was used	7	90

^a^Standard conditions: alkenol **1a** (0.2 mmol, 1 equiv), CBr_4_ (0.4 mmol, 2 equiv), Ru(bpy)_3_Cl_2_ (0.006 mmol, 3 mol %) in dry DMSO (0.1 M) irradiated by blue LEDs (1 W); ^b^isolated yield.

With the optimized reaction conditions in hand, various substituted butenols were subsequently investigated for the scope of the reaction. As shown in Table 2, electronically distinct styrenes ranging from electron-rich to electron-deficient provided good yields of the desired 5-*endo* bromoetherification products (Table 2, entries 1–16). Additionally, trisubstituted alkenols were also examined and showed high reactivity (Table 2, entries 17 and 18). The alkenol with geminal dimethyl substituent produced the expected 5-*endo* bromoetherification product in 90% yield (Table 2, entry 19).

**Table 2:** Photocatalytic bromoetherification of butenols.^a^

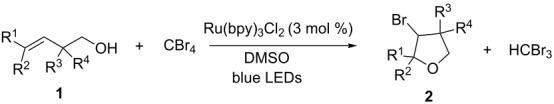			

Entry	Substrate	Product	Yield (%)^b^
	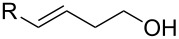	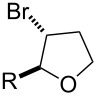	

1	R = 4-OMePh	**2a**	94
2	R = 3-OMePh	**2b**	93
3	R = 2-OMePh	**2c**	89
4	R = Ph	**2d**	88
5	R = 4-MePh	**2e**	90
6	R = 3-MePh	**2f**	85
7	R = 2-MePh	**2g**	84
8	R = 4-BrPh	**2h**	90
9	R = 3-BrPh	**2i**	87
10	R = 2-BrPh	**2j**	86
11	R = 4-FPh	**2k**	89
12	R = 4-NO_2_Ph	**2l**	74
13	R = 2,4-diOMePh	**2m**	93
14	R = 2,5-diOMePh	**2n**	84
15	R = 2-OMe-5-ClPh	**2o**	88
16	R = 2-OMe-naphthalen-1-yl	**2p**	86
	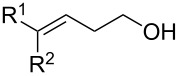	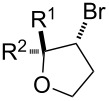	
17	R^1^ = 4-OTBDPSPh, R^2^ = Me	**2q**	87
18	R^1^ = R^2^ = 4-OMePh	**2r**	83
19	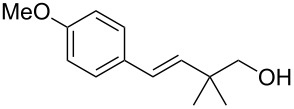	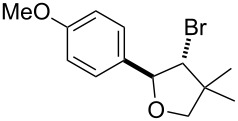 **2s**	90

^a^Standard conditions: butenol **1** (0.2 mmol, 1 equiv), CBr_4_ (0.4 mmol, 2 equiv), Ru(bpy)_3_Cl_2_ (0.006 mmol, 3 mol %) in dry DMSO (0.1 M) irradiated by blue LEDs (1W) for 4 h; ^b^isolated yield.

To further demonstrate the general value of this strategy, a number of longer-chain pentenols were prepared and submitted to the optimized reaction conditions. As can be seen in Table 3, various styrenes were reacted efficiently to form the substituted tetrahydropyrans in high yield via 6-*endo* bromoetherification (Table 3, entries 1 and 2). Furthermore, not only primary alcohols but also secondary alcohols were tolerated using the reaction conditions albeit a mixture of 6-*endo* and 5-*exo* bromoetherification products obtained (Table 3, entries 3 and 4). Interestingly, for terminal alkene, the 5-*exo* bromoetherification product was achieved in 84% yield (Table 3, entry 5).

**Table 3:** Photocatalytic bromoetherification of pentenols^a^.

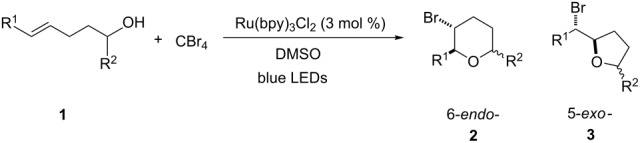			

Entry	Substrate	Product	Yield (%)^b^
	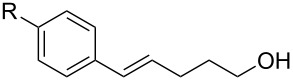	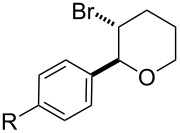	

1	R = 4-OMe	**2t**	91
2	R = 4-Me	**2u**	87

	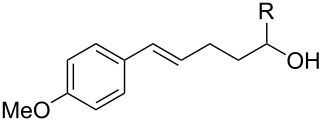	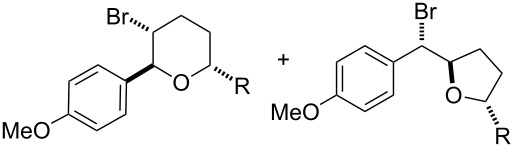	

3	R = Me	**2v**:**3v** = 1:1.1	74
4	R = Ph	**2w**:**3w** = 1:1.3	80
5	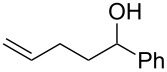	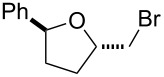 **3x**	84

^a^Standard conditions: pentenol **1** (0.2 mmol, 1 equiv), CBr_4_ (0.4 mmol, 2 equiv), Ru(bpy)_3_Cl_2_ (0.006 mmol, 3 mol %) in dry DMSO (0.1 M) irradiated by blue LEDs (1 W) for 4 hours; ^b^isolated yield.

**Scheme 1:**
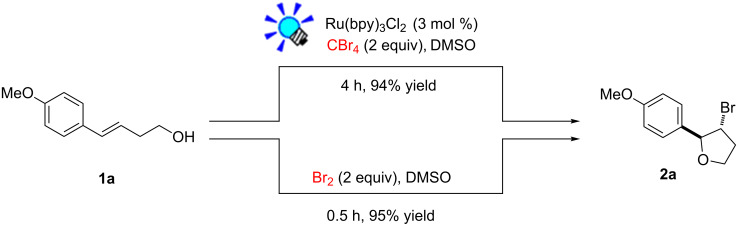
Control experiment with liquid bromine for bromoethrification of alkenols.

To add more credence to the involvement of bromine in this protocol, a control experiment was conducted by reaction of alkenol **1a** with liquid bromine in DMSO which led to *trans*-β-bromotetrahydrofuran **2a** in 95% yield (Scheme 1). Such a result is in accordance with the case of **1a** reacted under the standard reaction conditions of this protocol.

**Scheme 2:**
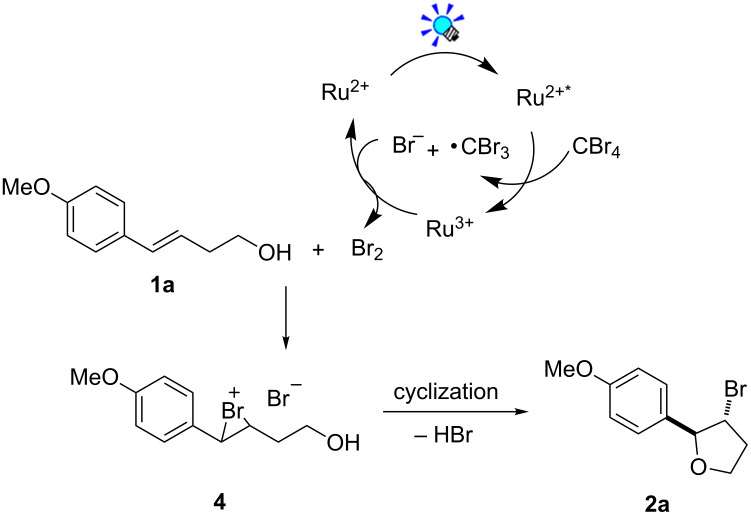
Proposed mechanism for the photocatalytic bromoetherification of alkenols.

Based upon the above results, the mechanism is proposed as shown in Scheme 2. Firstly, oxidative quenching of the visible-light-induced excited state Ru(bpy)_3_^2+^* by CBr_4_, generates Br^−^ along with the Ru(bpy)_3_^3+^ complex. Then bromine was generated in situ through the oxidation of Br^−^ by Ru(bpy)_3_^3+^ [16], sequential reaction with alkene **1a** forms the three-membered bromonium intermediate **4** [28]. Finally, intramolecular nucleophilic cyclization furnishes the desired product β-bromotetrahydrofuran **2a**.

## Conclusion

In summary, we have developed a mild and operationally simple method for the bromoetherification of alkenols with CBr_4_ as the bromine source, utilizing visible-light-induced phototedox catalysis. The reaction proceeds with high efficiency and regioselectivity for the synthesis of β-bromotetrahydrofurans and -tetrahydropyranes.

## Experimental

### General procedure for the photocatalytic bromoetherification of alkenols

To a 10 mL round bottom flask equipped with a magnetic stir bar were added alkenols **1** (0.2 mmol), CBr_4_ (132 mg, 0.4 mmol), Ru(bpy)_3_Cl_2_ (4.6 mg, 0.006 mmol) and dry DMSO (2 mL). The mixture was irradiated with blue LEDs (1 W) at room temperature without being degassed for 4 hours. Then water was added and the aqueous layer was extracted with ethyl acetate. The combined organic layers were washed with brine, dried over anhydrous Na_2_SO_4_ and concentrated. The residue was purified by flash column chromatography to give the final products **2**.

## Supporting Information

File 1 ^1^H and ^13^C NMR spectra for products.
