# Supplementary material for: Visible-light-induced bromoetherification of alkenols for the synthesis of β-bromotetrahydrofurans and -tetrahydropyrans
Source: Beilstein J Org Chem. 2015 Jan 8;11:31–6. doi: 10.3762/bjoc.11.5 (PMC4311724; doi:10.3762/bjoc.11.5)
Supplement: File 1 — 1H and 13C NMR spectra for products. [file Beilstein_J_Org_Chem-11-31-s001.pdf]

Supporting information

for

Visible-light-induced bromoetherification of alkenols for the synthesis of β-bromotetrahydrofurans and -tetrahydropyrans

Run Lin, Hongnan Sun, Chao Yang*, Youdong Yang, Xinxin Zhao and Wujiong Xia*

Address: State Key Lab of Urban Water Resource and Environment, the Academy of Fundamental and Interdisciplinary Sciences, Harbin Institute of Technology, Harbin 150080, P. R. China

Email: Chao Yang* - xyyang@hit.edu.cn; Wujiong Xia* - xiawj@hit.edu.cn

*Corresponding author

Additional experimental data and ^{1}H and ^{13}C NMR spectra for products

Contents

1. General information……………………………….………………………S2

2. Preparation and characterization of alkenols……………………………………...S2

3. General prodedure for photocatalytic bromoetherification of alkenols..………….S8

4. Stereochemical determination of β-bromotetrahydrofuran **2a**…………………....S9

5. Characterization of photocatalytic bromoetherification products…………….….S11

6. ^{1}H and ^{13}C NMR spectra……………..……………………….……………….…S20

1. General information

All reagents were purchased from commercial sources unless otherwise noted. Solvent was freshly distilled prior to use unless otherwise noted. Reactions were monitored by thin-layer chromatography (TLC) and visualized by UV-light (254 nm) or by treatment with a solution of 10 g phosphomolybdic acid and 100 mL EtOH followed by heating. ^{1}H NMR (400 MHz) and ^{13}C NMR (100 MHz) spectra were obtained on Bruker AV-400 instrument. Chemical shifts for ^{1}H NMR spectra were reported in δ ppm referenced to an internal $SiMe_4$ standard. Chemical shifts for ^{13}C NMR spectra were reported in parts per million relative to the center line signal of the CDCl3 triplet at 77.0 ppm. HR-ESI-MS spectra were recorded on a Bruker Esquire LC mass spectrometer using electrospray ionization.

2. Preparation and characterization of alkenols

Alkenols **1a-s** were prepared according to reported method[1].

(*E*)-4-(4-methoxyphenyl)but-3-en-1-ol (1a)

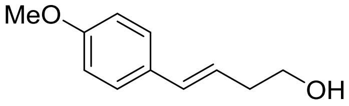

^{1}HNMR ($CDCl_3$, 400 MHz): δ 7.27 (d, J = 8.7 Hz, 2H), 6.82 (d, J = 8.7 Hz, 2H), 6.40 (d, J = 15.9 Hz, 1H), 6.03 (dt, J = 15.7, 7.1 Hz, 1H), 3.77 (s, 3H), 3.69 (t, J = 6.4 Hz, 2H), 2.42 (td, J = 7.3, 1.0 Hz, 2H), 2.18 (brs, 1H). Data are consistent with literature values.[1]

(*E*)-4-(3-methoxyphenyl)but-3-en-1-ol (1b)

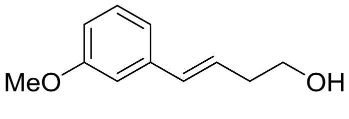

^{1}HNMR ($CDCl_3$, 400 MHz): δ 7.21 (t, J = 7.9 Hz, 1H), 6.95 (d, J = 7.7 Hz, 1H), 6.89 (s, 1H), 6.77 (dd, J = 8.2, 2.4 Hz, 1H), 6.45 (d, J = 15.9 Hz, 1H), 6.23-6.16 (m, 1H), 3.80 (s, 1H), 3.74 (t, J = 6.3 Hz, 2H), 2.47 (q, J = 6.5 Hz, 2H), 1.84 (brs, 1H). Data are

1 Zeng, X. H.; Miao, C. X.; Wang, S. F.; Xia, C. G.; Sun, W. *Chem. Commun.* 2013, 49, 2418.

consistent with literature values. [1]

(*E*)-4-(2-methoxyphenyl)but-3-en-1-ol (1c)

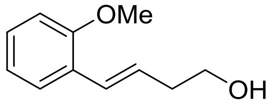

^{1}HNMR ($CDCl_3$, 400 MHz): δ 7.43-7.40 (m,1H), 7.26-7.18 (m, 1H), 6.95-6.79 (m, 3H), 6.19 (dt, J = 15.9, 7.2 Hz, 1H), 3.84 (s, 3H), 3.76-3.69 (m, 2H), 2.53-2.47 (m, 2H), 1.68 (brs, 1H). Data are consistent with literature values. [1]

(*E*)-4-phenylbut-3-en-1-ol (1d)

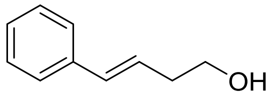

^{1}HNMR ($CDCl_3$, 400 MHz): δ 7.36-7.19 (m, 5H), 6.48 (d, J = 15.9 Hz, 1H), 6.20 (dt, J = 15.8, 7.1 Hz, 1H), 3.76-3.73 (m, 2H), 2.50-2.45 (m, 2H), 1.81 (brs, 1H). Data are consistent with literature values. [1]

(*E*)-4-*p*-tolylbut-3-en-1-ol (1e)

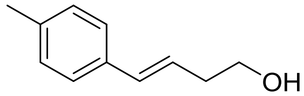

^{1}HNMR ($CDCl_3$, 400 MHz): δ 7.16 (d, J = 8.0 Hz, 2H), 7.01 (d, J = 8.0 Hz, 2H), 6.36 (d, J = 15.9 Hz, 1H), 6.05 (dt, J = 15.8, 7.1 Hz, 1H), 3.64-3.60 (m, 2H), 2.36 (td, J = 7.3, 0.9 Hz, 2H), 2.23 (s, 3H), 2.00 (brs, 1H). Data are consistent with literature values. [1]

(*E*)-4-*m*-tolylbut-3-en-1-ol (1f)

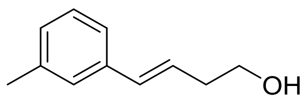

^{1}HNMR ($CDCl_3$, 400 MHz): δ 7.24-7.14 (m, 3H), 7.03 (d, J = 7.0 Hz, 1H), 6.46 (d, J = 15.9 Hz, 1H), 6.18 (dt, J = 15.7, 7.1 Hz, 1H), 3.73 (t, J = 6.3 Hz, 2H), 2.49-2.44 (m, 2H), 2.33 (s, 3H), 1.66 (brs, 1H). Data are consistent with literature values. [1]

(*E*)-4-*o*-tolylbut-3-en-1-ol (1g)

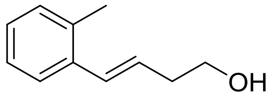

^{1}HNMR ($CDCl_3$, 400 MHz): δ 7.16-7.12 (m, 4H), 6.68 (d, J = 15.7 Hz, 1H), 6.06 (dt,

J = 15.5, 7.2 Hz, 1H), 3.73 (t, *J* = 6.4 Hz, 2H), 2.49 (td, *J* = 7.6, 1.3 Hz, 2H), 2.33 (s, 3H), 1.81 (brs, 1H). Data are consistent with literature values. [1]

(*E*)-4-(4-bromophenyl)but-3-en-1-ol (1h)

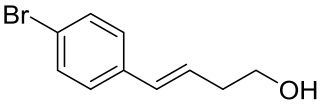

^{1}HNMR ($CDCl_3$, 400 MHz): δ 7.41 (d, *J* = 8.4 Hz, 2H), 7.20 (d, *J* = 8.4 Hz, 2H), 6.41 (d, *J* = 15.9 Hz, 1H), 6.19 (dt, *J* = 15.7, 7.1 Hz, 1H), 3.76-3.73 (m, 2H), 2.48-2.43 (m, 2H), 1.72 (brs, 1H). Data are consistent with literature values. [1]

(*E*)-4-(3-bromophenyl)but-3-en-1-ol (1i)

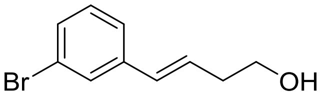

^{1}HNMR ($CDCl_3$, 400 MHz): δ 7.50 (s, 1H), 7.32 (d, *J* = 7.9 Hz, 1H), 7.26-7.23 (m, 1H), 7.15 (t, *J* = 7.8 Hz, 1H), 6.40 (d, *J* = 15.9 Hz, 1H), 6.21 (dt, *J* = 15.8, 7.1 Hz, 1H), 3.74 (t, *J* = 6.3 Hz, 2H), 2.47 (td, *J* = 7.3, 1.1 Hz, 1H), 1.83 (brs, 1H). Data are consistent with literature values.[2]

(*E*)-4-(2-bromophenyl)but-3-en-1-ol (1j)

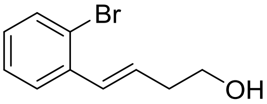

^{1}HNMR ($CDCl_3$, 400 MHz): δ 7.33-7.24 (m, 4H), 6.43 (d, *J* = 15.9 Hz, 1H), 6.18 (dt, *J* = 15.7, 7.1 Hz, 1H), 3.75 (t, *J* = 6.3 Hz, 2H), 2.50-2.45 (m, 2H), 1.84(brs, 1H). Data are consistent with literature values. [2]

(*E*)-4-(4-fluorophenyl)but-3-en-1-ol (1k)

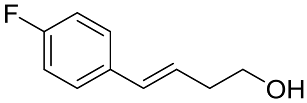

^{1}HNMR ($CDCl_3$, 400 MHz): δ 7.32-7.29 (m, 2H), 6.98 (t, *J* = 8.7 Hz, 2H), 6.44 (d, *J* = 15.9 Hz, 1H), 6.15-6.07 (m, 1H), 3.74 (t, *J* = 6.3 Hz, 2H), 2.49-2.44 (m, 3H). Data are consistent with literature values. [1]

---

2 Zeng, X. H.; Miao, C. X.; Wang, S. F.; Xia, C. G.; Sun, W. *Synthesis*, 2013, 45, 2391.

(E)-4-(4-nitrophenyl)but-3-en-1-ol (1l)

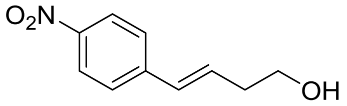

^{1}HNMR ($CDCl_3$, 400 MHz): δ 8.14 (d, J = 8.4 Hz, 2H), 7.47 (d, J = 8.4 Hz, 2H), 6.56 (d, J = 16.0 Hz, 1H), 6.49-6.42 (m, 1H), 3.81 (t, J = 6.1 Hz, 2H), 2.54 (q, J = 6.0 Hz, 2H), 1.79 (brs, 1H). ^{13}C NMR ($CDCl_3$, 100 MHz): δ 146.5, 143.7, 132.1, 130.5, 126.5, 123.9, 61.6, 36.4.

(*E*)-4-(2,4-dimethoxyphenyl)but-3-en-1-ol (1m)

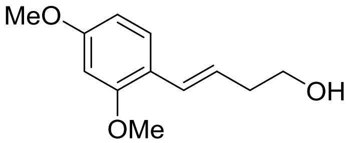

^{1}HNMR ($CDCl_3$, 400 MHz): δ 7.35 (d, J = 8.3 Hz, 1H), 6.74 (d, J = 16.0 Hz, 1H), 6.50-6.45 (m, 2H), 6.10 (dt, J = 15.9, 7.2 Hz, 1H), 3.84 (s, 3H), 3.83 (s, 3H), 3.75 (t, J = 6.3 Hz, 2H), 2.53-2.47 (m, 2H), 1.74 (brs, 1H); ^{13}C NMR ($CDCl_3$, 100 MHz): δ 160.1, 157.4, 127.2, 127.2, 124.7, 119.4, 104.7, 98.38, 62.1, 55.4, 55.3, 36.8.

(*E*)-4-(2,5-dimethoxyphenyl)but-3-en-1-ol (1n)

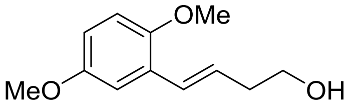

^{1}HNMR ($CDCl_3$, 400 MHz): δ 6.85 (d, J = 2.8 Hz, 1H), 6.78 (dd, J = 8.5, 5.8 Hz, 1H), 6.63 (d, J = 11.6 Hz, 1H), 5.75 (dt, J = 11.6, 7.5 Hz, 1H), 3.78 (s, 3H), 3.77 (s, 3H), 3.71 (t, J = 6.4 Hz, 2H), 2.51 (dd, J = 7.5, 1.5 Hz, 2H), 1.72 (brs, 1H).

(*E*)-4-(5-chloro-2-methoxyphenyl)but-3-en-1-ol (1o)

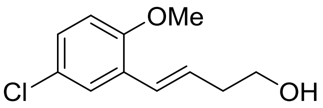

^{1}HNMR ($CDCl_3$, 400 MHz): δ 7.38 (d, J = 2.5 Hz, 1H), 7.13 (dd, J = 8.7, 2.5 Hz, 1H), 6.78-6.72 (m, 2H), 6.23-6.16 (m, 1H), 3.81 (s, 3H), 3.75 (t, J = 6.3 Hz, 2H), 2.49 (d, J = 6.8 Hz, 2H), 1.67 (brs, 1H).

(*E*)-4-(2-methoxynaphthalen-1-yl)but-3-en-1-ol (1p)

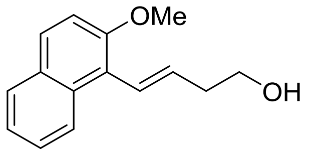

^{1}HNMR ($CDCl_3$, 400 MHz): δ 8.02 (d, J = 8.6 Hz, 1H), 7.68 (t, J = 8.4 Hz, 1H), 7.63 (d, J = 9.0 Hz, 1H), 7.34 (t, J = 7.2 Hz, 1H), 7.24 (dd, J = 12.8, 5.9 Hz, 1H), 7.16 (t, J = 8.6 Hz,1H), 6.73 (d, J = 16.1 Hz, 1H), 6.02 (dt, J = 15.9, 7.0 Hz, 1H), 3.82 (s, 3H), 3.70 (t, J = 6.3 Hz, 2H), 2.52 (q, J = 6.2 Hz, 2H). ^{13}C NMR ($CDCl_3$, 100 MHz): δ 153.0, 131.9, 130.6, 128.2, 127.4, 127.2, 125.3, 124.8, 123.3, 122.4, 119.8, 112.1, 60.8, 55.4, 36.3.

(*E*)-4-(4-((*tert*-butyldiphenylsilyl)oxy)phenyl)pent-3-en-1-ol (1q)

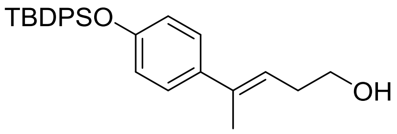

^{1}HNMR ($CDCl_3$, 400 MHz): δ 7.66 (dd, J = 7.9, 1.4 Hz, 4H), 7.34 (dd, J = 5.0, 3.6 Hz, 2H), 7.29 (t, J = 7.1 Hz, 4H), 6.86 (d, J = 8.6 Hz, 2H), 6.65 (d, J = 8.6 Hz, 2H), 5.30 (td, J = 7.3, 1.2 Hz, 1H), 3.50 (t, J = 6.5 Hz, 2H), 2.15 (q, J = 6.5 Hz, 2H), 1.90 (s, 3H), 1.04 (s, 9H); ^{13}C NMR (100 MHz, $CDCl_3$): δ 154.3, 139.0, 135.59, 134.2, 133.0, 129.8, 128.7, 127.7, 122.4, 119.3, 62.7, 35.4, 32.6, 26.5, 19.4.

4,4-bis(4-methoxyphenyl)but-3-en-1-ol (1r)

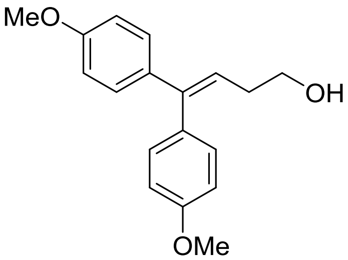

^{1}HNMR ($CDCl_3$, 400 MHz): δ 7.18-7.14 (m, 2H), 7.12-7.09 (m, 2H), 6.92-6.88 (m, 2H), 6.81-6.78 (m, 2H), 5.96 (t, J = 7.5 Hz, 1H), 3.83 (s, 3H), 3.78 (s, 3H), 3.70 (t, J = 6.6 Hz, 2H), 2.40 (dd, J = 14.0, 6.7 Hz, 2H); ^{13}C NMR ($CDCl_3$, 100 MHz): δ 158.8, 158.6, 143.4, 135.5, 132.3, 131.0, 128.4, 123.2, 113.6, 113.4, 62.7, 55.3, 55.2, 33.3.

(*E*)-4-(4-methoxyphenyl)-2,2-dimethylbut-3-en-1-ol (1s)

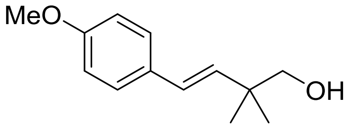

^{1}HNMR ($CDCl_3$, 400 MHz): δ 7.12 (d, J = 8.2 Hz, 2H), 6.83 (d, J = 8.6 Hz, 2H), 6.54 (d, J = 12.6 Hz, 1H), 5.50 (d, J = 12.6 Hz, 1H), 3.79 (s, 3H), 3.31 (s, 2H), 0.97 (s, 6H).^{13}C NMR ($CDCl_3$, 100 MHz): δ 158.3, 138.2, 129.7, 127.2, 113.9, 113.1, 71.9, 55.1, 39.5, 25.7.

Alkenols **1t-w** were prepared according to reported method.[3]

(*E*)-5-(4-methoxyphenyl)pent-4-en-1-ol (1t)

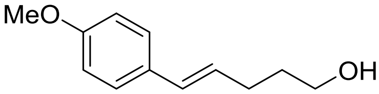

^{1}HNMR ($CDCl_3$, 400 MHz): δ 7.21 (d, J = 8.7 Hz, 2H), 6.86 (d, J = 8.7 Hz, 2H), 6.34 (d, J = 11.0 Hz, 1H), 6.06 (dt, J = 15.8, 6.9 Hz, 1H), 3.79 (s, 3H), 3.64 (t, J = 5.7 Hz, 2H), 3.44 (brs, 1H), 2.43-2.37 (m, 1.8 Hz, 2H), 1.75-1.67 (m, 2H). Data are consistent with literature values.[4]

(*E*)-5-*p*-tolylpent-4-en-1-ol (1u)

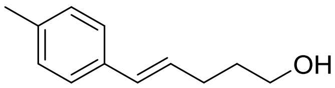

^{1}HNMR ($CDCl_3$, 400 MHz): δ 7.23 (d, J = 8.1 Hz, 2H), 7.09 (d, J = 7.9 Hz, 2H), 6.38 (d, J = 15.8 Hz, 1H), 6.16 (dt, J = 15.7, 6.9 Hz, 1H), 3.68 (t, J = 6.5 Hz, 2H), 2.33-2.26 (m, 5H), 1.81-1.69 (m, 3H). Data are consistent with literature values.[5]

(*E*)-6-(4-methoxyphenyl)hex-5-en-2-ol (1v)

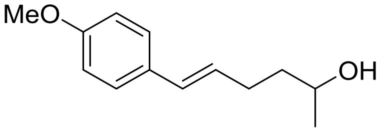

^{1}HNMR ($CDCl_3$, 400 MHz): δ 7.27 (d, J = 8.1 Hz, 2H), 6.83 (d, J = 8.6 Hz, 2H), 6.36

---

3 Logan, A.W.J.; Parker, J.S.; Hallside, M.S.; Burton J.W. *Org. Lett.* 2012, 14, 2940.

4 Belanger, G.; Levesque, F.; Paquet, J.; Barbe, G. *J. Org. Chem.* 2005, 70, 291.

5 Crombie, L.; Wyvill, R. D. *J. Chem. Soc. Perkin Trans. 1* 1985, 1983.

(d, J = 16.0 Hz, 1H), 6.12-6.04 (m, 1H), 3.88-3.83 (m, 1H), 3.79 (s, 3H), 2.34-2.25 (m, 2H), 1.67-1.57 (m, 2H), 1.47 (brs, 1H), 1.22 (d, J = 6.2 Hz, 3H); ^{13}C NMR ($CDCl_3$, 100 MHz): δ ^{13}C NMR (101 MHz, $CDCl_3$) δ 158.7, 130.5, 129.6, 128.1, 127.0, 113.9, 67.6, 55.3, 38.8, 29.3, 23.5.

(*E*)-5-(4-methoxyphenyl)-1-phenylpent-4-en-1-ol (1w)

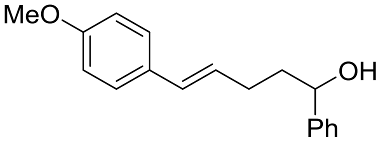

^{1}HNMR ($CDCl_3$, 400 MHz): δ 7.37-7.25 (m, 7H), 6.83 (d, J = 8.7 Hz, 2H), 6.35 (d, J = 15.8 Hz, 1H), 6.08 (dt, J = 15.7, 6.9 Hz, 1H), 4.74 (t, J = 6.5 Hz, 1H), 3.79 (s, 3H), 2.35-2.21 (m, 2H), 2.02-1.85 (m, 3H); ^{13}C NMR ($CDCl_3$, 100 MHz): δ158.7, 144.6, 130.5, 129.8, 128.5, 127.8, 127.6, 127.0, 125.9, 113.9, 74.0, 55.3, 38.6, 29.3.

1x was prepared according to reported method.[6]

1-phenylpent-4-en-1-ol (1x)

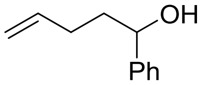

^{1}HNMR ($CDCl_3$, 400 MHz): δ 7.42-7.29 (m, 5H), 5.93-5.83 (m, 1H), 5.11-5.02 (m, 2H), 4.72-4.68 (m, 1H), 2.45-2.08 (m, 3H), 1.97-1.78 (m, 2H). Data are consistent with literature values.[7]

3. General prodedure for photocatalytic bromoetherification of alkenols

General method: To a 10 mL round bottom flask equipped with a magnetic stir bar were added alkenols **1** (0.2 mmol), CBr_4 (132 mg, 0.4 mmol), Ru(bpy)$_3$Cl$_2$ (4.6 mg, 0.006mmol) and dry DMSO (2 mL). The mixture was irradiated with blue LEDs (1W) at room temperature without degassed for 4 hours. Then water was added and the aqueous layer was extracted with ethyl acetate. The combined organic layers were washed with brine, dried over anhydrous Na_2SO_4, concentrated. The residue was purified by flash column chromatography to give the final product **2**.

6 Janza, B.; Studer, A. *J. Org. Chem.* 2005, 70, 6991.
7 Rawal, V.; Singh, S. P.; Dufour, C.; Michoud, C. *J. Org. Chem.* 1993, *58,* 7734.

4. Stereochemical determination of β-bromotetrahydrofuran 2a

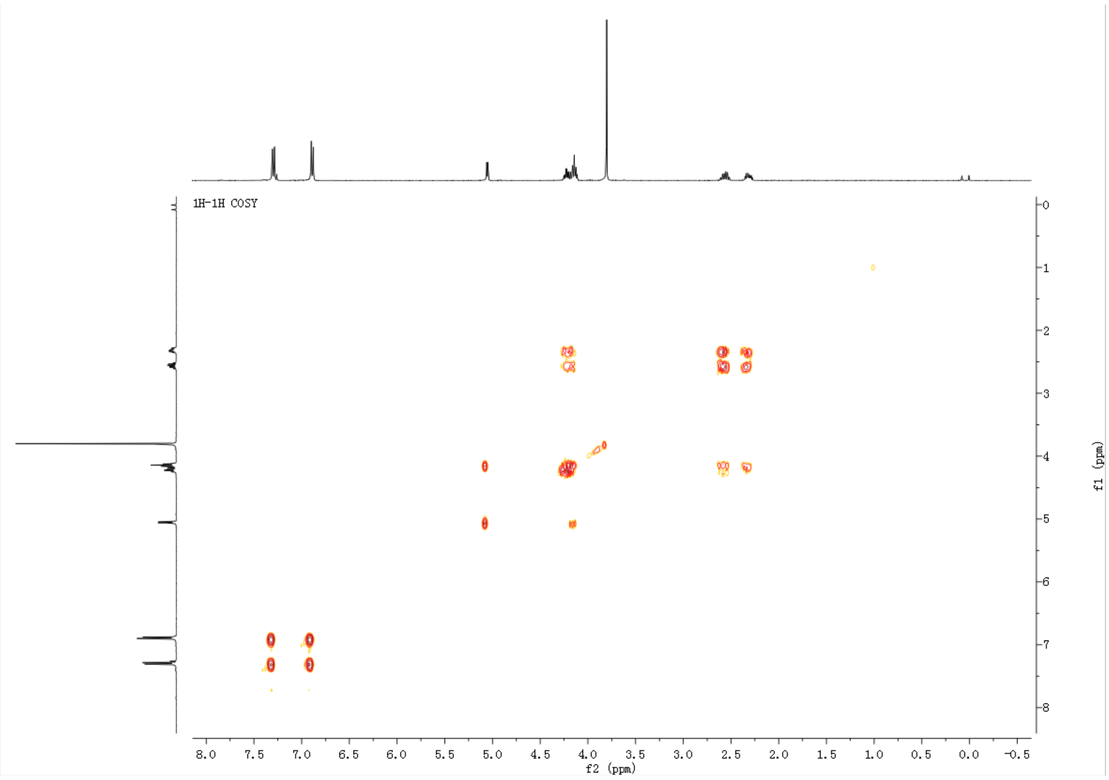

Fig. 1 COSY spectrum of the product **2a**.

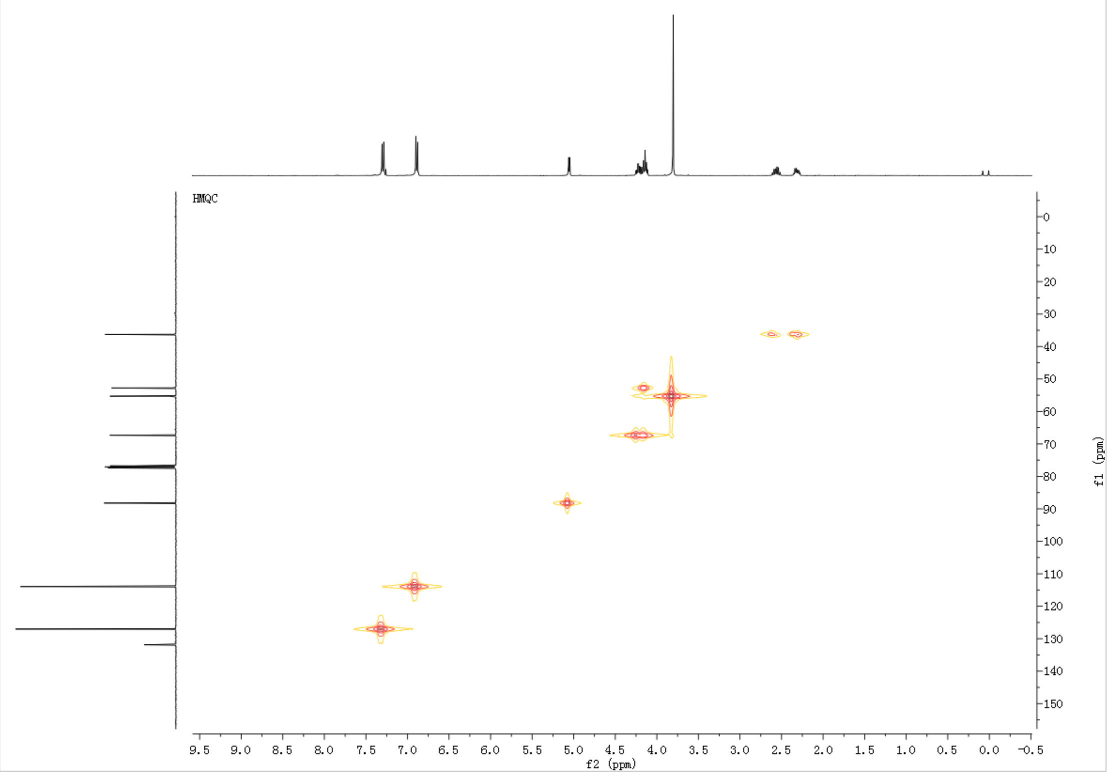

Fig. 2 HMQC spectrum of the product **2a**.

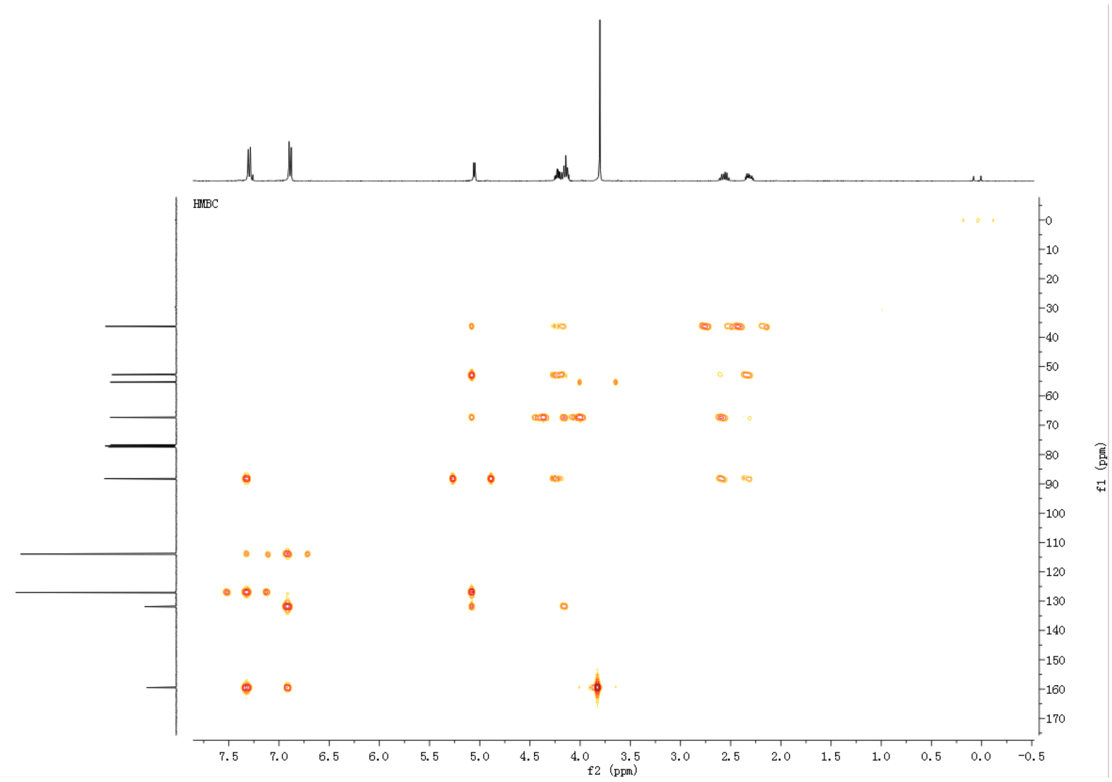

Fig. 3 HMBC spectrum of the product **2a**.

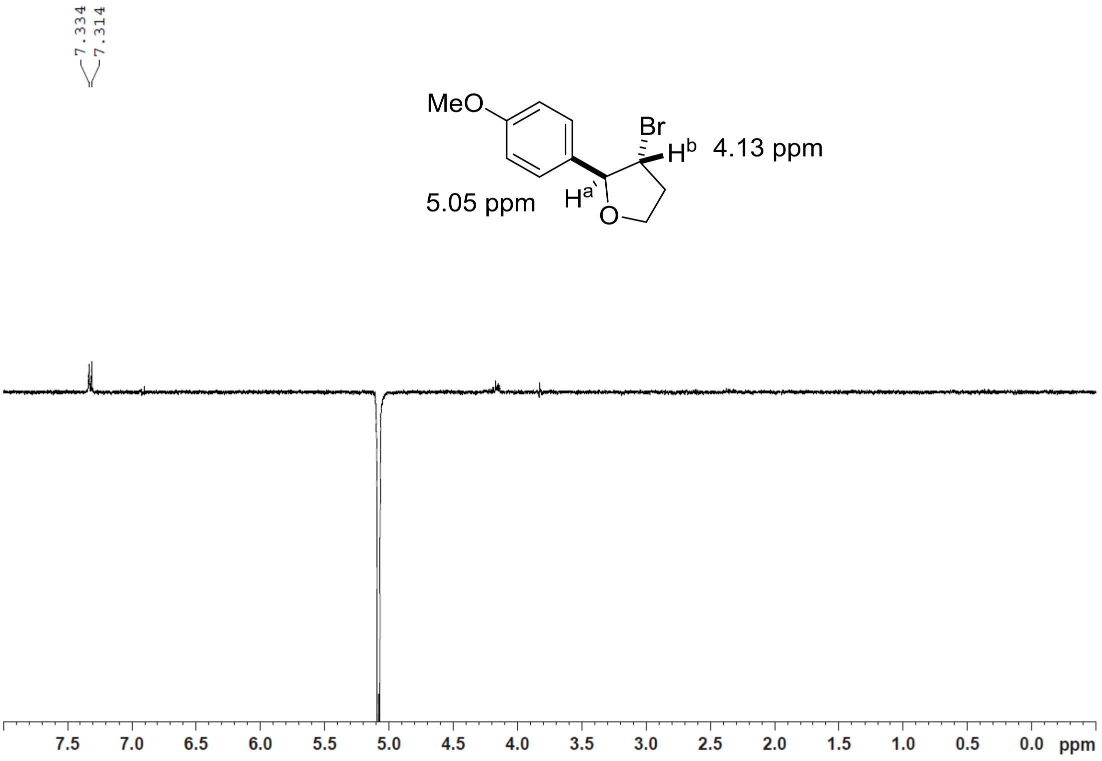

Fig. 4 NOE spectrum of the product **2a**. (There is no NOE between H^a and H^b.)

The NOE data indicate that product **2a** should be *trans* configuration. The stereo-chemistry of all other products was inferred by analogy.

5. Characterization of photocatalytic bromoetherification products

3-bromo-2-(4-methoxyphenyl)tetrahydrofuran (2a)

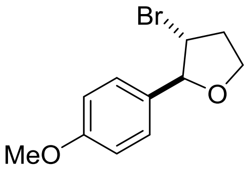

^{1}HNMR ($CDCl_3$, 400 MHz): δ 7.30 (d, J = 8.7 Hz, 2H), 6.89 (d, J = 8.7 Hz, 2H), 5.05 (d, J = 5.0 Hz, 1H), 4.23 (td, J = 8.2, 4.1 Hz, 1H), 4.19-4.10 (m, 2H), 3.80 (s, 3H), 2.56 (dd, J = 13.7, 7.3 Hz, 1H), 2.37-2.26 (m, 1H); ^{13}C NMR ($CDCl_3$, 100 MHz): δ 159.5, 131.9, 127.0, 114.0, 88.2, 67.3, 55.3, 52.8, 36.3. HRMS-ESI (*m*/*z*): $[M+H]^+$ calculated for $C_{11}H_{14}BrO_2$ 257.0177, found 257.0176.

3-bromo-2-(3-methoxyphenyl)tetrahydrofuran (2b)

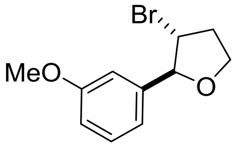

^{1}HNMR ($CDCl_3$, 400 MHz): δ 7.27 (t, J = 7.9 Hz, 2H), 6.94 (dd, J = 13.5, 4.8 Hz, 2H), 6.84 (dd, J = 8.1, 2.5 Hz, 1H), 5.13 (d, J = 4.5 Hz, 1H), 4.29 – 4.15 (m, 3H), 3.82 (s, 3H), 2.54 (dd, J = 13.7, 7.0 Hz, 1H), 2.39 – 2.26 (m, 1H); ^{13}C NMR ($CDCl_3$, 100 MHz): δ 159.8, 141.8, 129.6, 117.9, 113.5, 111.2, 88.4, 67.5, 55.3, 52.7, 36.2. HRMS-ESI (*m*/*z*): $[M+H]^+$ calculated for $C_{11}H_{14}BrO_2$ 257.0177, found 257.0175.

3-bromo-2-(2-methoxyphenyl)tetrahydrofuran (2c)

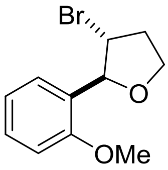

^{1}HNMR ($CDCl_3$, 400 MHz): δ 7.35-7.28 (m, 1H), 7.29-7.23 (m, 1H), 6.95 (t, J = 7.4 Hz, 1H), 6.87 (d, J = 8.2 Hz, 1H), 5.52 (s, 1H), 4.50-4.41 (m, 1H), 4.37-4.23 (m, 2H), 3.88 (s, 3H), 2.44-2.29 (m, 1H), 2.28-2.17 (m, 1H); ^{13}C NMR ($CDCl_3$, 100 MHz): δ 156.2, 129.4, 128.9, 126.1, 120.5, 110.2, 85.2, 67.7, 55.4, 53.3, 35.3. HRMS-ESI (*m*/*z*): $[M+H]^+$ calculated for $C_{11}H_{14}BrO_2$ 257.0177, found 257.0177.

3-bromo-2-phenyltetrahydrofuran (2d)

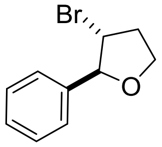

^{1}HNMR ($CDCl_3$, 400 MHz): δ 7.43-7.33 (m, 4H), 7.32 (dd, *J* = 5.8, 2.9 Hz, 1H), 5.14 (d, *J* = 4.7 Hz, 1H), 4.26 (td, *J* = 8.2, 4.0 Hz, 1H), 4.23-4.15 (m, 2H), 2.56 (dd, *J* = 13.8, 7.2 Hz, 1H), 2.40-2.26 (m, 1H), 2.28-2.17 (m, 1H); ^{13}C NMR ($CDCl_3$, 100 MHz): δ 140.0, 128.5, 128.1, 125.6, 88.5, 67.5, 52.7, 36.2. HRMS-ESI (*m*/*z*): $[M+H]^+$ calculated for $C_{10}H_{12}BrO$ 227.0072, found 227.0070. Data are consistent with literature values.[8]

3-bromo-2-*p*-tolyltetrahydrofuran (2e)

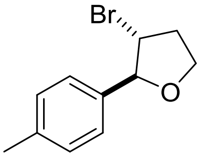

^{1}HNMR ($CDCl_3$, 400 MHz): δ 7.26 (d, *J* = 7.9 Hz, 2H), 7.17 (d, *J* = 7.9 Hz, 2H), 5.10 (d, *J* = 4.7 Hz, 1H), 4.30-4.20 (m, 1H), 4.20-4.12 (m, 2H), 2.64-2.45(m, 1H), 2.35 (s, 3H), 2.33-2.27 (m, 1H); ^{13}C NMR ($CDCl_3$, 100 MHz): δ 137.8, 137.0, 129.2, 125.5, 88.4, 67.4, 52.8, 36.2, 21.1. HRMS-ESI (*m*/*z*): $[M+H]^+$ calculated for $C_{11}H_{14}BrO$ 241.0228, found 241.0230.

3-bromo-2-*m*-tolyltetrahydrofuran (2f)

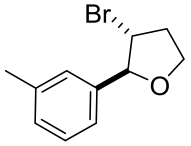

^{1}HNMR ($CDCl_3$, 400 MHz): δ 7.28 (dd, *J*= 6.7, 4.7 Hz, 1H), 7.24 (d, *J* = 7.5 Hz, 1H), 7.17 (d, *J* = 8.9 Hz, 2H), 7.12 (d, *J* = 7.4 Hz, 1H), 5.11 (d, *J* = 4.6 Hz, 1H), 4.26 (td, *J* = 8.2, 4.0 Hz, 1H), 4.22-4.16 (m, 2H), 2.55 (dt, *J* = 15.1, 8.0 Hz, 1H), 2.36 (s, 3H), 2.35-2.24 (m, 1H); ^{13}C NMR ($CDCl_3$, 100 MHz): δ 139.9, 138.2, 128.8, 128.4, 126.2, 122.8, 88.6, 67.5, 52.8, 36.2, 21.4. HRMS-ESI (*m*/*z*): $[M+H]^+$ calculated for $C_{11}H_{14}BrO$ 241.0228, found 241.0230.

---

[8] Crich, D.; Sartillo-Piscil, F.; Quintero-Cortes, L.; Wink, D. J. *J. Org. Chem.* 2002, 67, 3360-3364.

3-bromo-2-*o*-tolyltetrahydrofuran (2g)

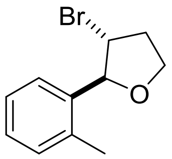

^{1}HNMR ($CDCl_3$, 400 MHz): δ 7.33 (dd, J = 5.1, 3.9 Hz, 1H), 7.20 (dd, J = 5.9, 3.3 Hz, 2H), 7.17 (dd, J = 8.1, 3.7 Hz, 2H), 5.44 (d, J = 2.5 Hz, 1H), 4.38 (td, J = 8.2, 2.2 Hz, 1H), 4.33-4.22 (m, 2H), 2.54-2.44 (m, 1H), 2.24 (s, 3H), 2.28 (ddt, J = 13.9, 5.7, 2.3 Hz, 1H); ^{13}C NMR ($CDCl_3$, 100 MHz): δ 139.2, 134.9, 130.4, 127.8, 126.1, 124.9, 86.7, 67.9, 52.9, 35.6, 19.6. HRMS-ESI (*m*/*z*): $[M+H]^+$ calculated for $C_{11}H_{14}BrO$ 241.0228, found 241.0227.

3-bromo-2-(4-bromophenyl)tetrahydrofuran (2h)

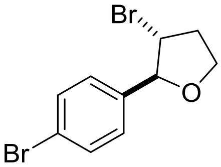

^{1}HNMR ($CDCl_3$, 400 MHz): δ 7.48 (d, J = 8.4 Hz, 2H), 7.27 (d, J = 8.2 Hz, 2H), 5.05 (d, J = 5.1 Hz, 1H), 4.23 (td, J = 8.2, 4.3 Hz, 1H), 4.17 (dd, J = 15.5, 8.2 Hz, 1H), 4.08 (dt, J = 7.0, 4.9 Hz, 1H), 2.54 (td, J = 15.1, 7.7 Hz, 1H), 2.39-2.25 (m, 1H); ^{13}C NMR ($CDCl_3$, 100 MHz): δ 139.0, 131.8, 127.4, 122.0, 87.8, 67.5, 52.1, 36.3. HRMS-ESI (*m*/*z*): $[M+H]^+$ calculated for $C_{10}H_{11}Br_2O$ 306.9156, found 306.9158.

3-bromo-2-(3-bromophenyl)tetrahydrofuran (2i)

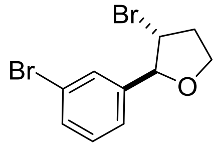

^{1}HNMR ($CDCl_3$, 400 MHz): δ 7.54 (s, 1H), 7.44 (d, J = 7.9 Hz, 1H), 7.31 (d, J = 7.7 Hz, 1H), 7.22 (t, J = 7.8 Hz, 2H), 5.08 (d, J = 4.8 Hz, 1H), 4.31-4.08 (m, 3H), 2.53 (dt, J = 15.0, 7.5 Hz, 1H), 2.39-2.27 (m, 1H); ^{13}C NMR ($CDCl_3$, 100 MHz): δ 142.4, 131.2, 130.1, 128.7, 124.4, 122.8, 87.7, 67.6, 52.2, 36.3. HRMS-ESI (*m*/*z*): $[M+H]^+$ calculated for $C_{10}H_{11}Br_2O$ 306.9156, found 306.9153.

3-bromo-2-(2-bromophenyl)tetrahydrofuran (2j)

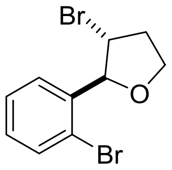

^{1}HNMR ($CDCl_3$, 400 MHz): δ 7.56 (d, *J* = 7.9 Hz, 1H), 7.43-7.27 (m, 2H), 7.21-7.12 (m, 1H), 5.54 (s, 1H), 4.50-4.32 (m, 3H), 2.54-2.20 (m, 2H); ^{13}C NMR ($CDCl_3$, 100 MHz): δ 140.7, 132.9, 129.4, 127.5, 127.1, 121.8, 88.5, 68.5, 53.0, 34.7. HRMS-ESI (*m*/*z*): $[M+H]^+$ calculated for $C_{10}H_{11}Br_2O$ 306.9156, found 306.9157.

3-bromo-2-(4-fluorophenyl)tetrahydrofuran (2k)

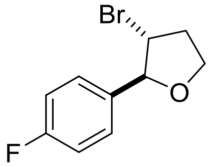

^{1}HNMR ($CDCl_3$, 400 MHz): δ 7.36 (dd, *J* = 8.5, 5.4 Hz, 2H), 7.04 (t, *J* = 8.7 Hz, 2H), 5.06 (d, *J* = 5.2 Hz, 1H), 4.28-4.20 (m, 1H), 4.20-4.13 (m, 1H), 4.10 (dt, *J* = 7.0, 5.0 Hz, 3H), 2.56 (dd, *J* = 13.7, 7.4 Hz, 1H), 2.38-2.25 (m, 1H); ^{13}C NMR ($CDCl_3$, 100 MHz): δ 163.8, 161.3, 135.6, 135.5, 127.5, 127.4, 115.5, 115.3, 87.8, 67.4, 52.3, 36.3. ^{19}F NMR (376MHz, $CDCl_3$): δ -114.2 (S). HRMS-ESI (*m*/*z*): $[M+H]^+$ calculated for $C_{10}H_{11}BrFO$ 244.9977, found 244.9980.

3-bromo-2-(4-nitrophenyl)tetrahydrofuran (2l)

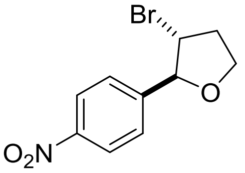

^{1}HNMR ($CDCl_3$, 400 MHz): δ 8.23 (d, *J* = 8.0 Hz, 2H), 7.60 (d, *J* = 8.3 Hz, 2H), 5.16 (d, *J* = 5.0 Hz, 1H), 4.30-4.19 (m, 2H), 4.08 (dd, *J* = 11.0, 5.7 Hz, 1H), 2.62-2.53 (m, 1H), 2.41-2.33 (m, 1H). ^{13}C NMR ($CDCl_3$, 100 MHz): δ 147.8, 147.0, 126.5, 123.8, 87.3, 67.8, 51.3, 36.4. HRMS-ESI (*m*/*z*): $[M+H]^+$ calculated for $C_{10}H_{11}BrNO_3$ 271.9922, found 271.9922.

3-bromo-2-(2,4-dimethoxyphenyl)tetrahydrofuran (2m)

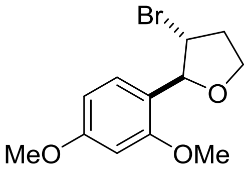

93%, ^{1}HNMR ($CDCl_3$, 400 MHz): δ 7.20 (d, J = 7.9 Hz, 1H), 6.50-6.41 (m, 2H), 5.42 (d, J = 1.9 Hz, 1H), 4.46-4.38 (m, 1H), 4.34-4.20 (m, 2H), 3.84 (s, 3H), 3.80 (s, 3H), 2.38 (d, J = 9.9 Hz, 1H), 2.23 (d, J = 5.8 Hz, 1H); ^{13}C NMR ($CDCl_3$, 100 MHz): δ 160.7, 157.3, 127.0, 121.7 103.9, 98.4, 85.1, 67.5, 55.4, 55.4, 53.2, 35.3. HRMS-ESI (*m/z*): $[M+H]^+$ calculated for $C_{12}H_{16}BrO_3$ 287.0283, found 287.0280.

3-bromo-2-(2,5-dimethoxyphenyl)tetrahydrofuran (2n)

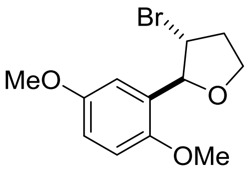

^{1}HNMR ($CDCl_3$, 400 MHz): δ 6.91 (m, 1H), 6.78 (d, J= 2.3 Hz, 2H), 5.48 (s, 1H), 4.44 (d, J= 5.4 Hz, 1H), 4.31 (m, 2H), 3.83 (s, 3H), 3.77 (s, 3H), 2.41-2.28 (m, 1H), 2.26-2.17 (m, 1H); ^{13}C NMR ($CDCl_3$, 100 MHz): δ 153.7, 150.4, 130.7, 112.9, 112.7, 111.1, 85.4, 67.8, 55.9, 55.8, 53.3, 35.2. HRMS-ESI (*m/z*): $[M+H]^+$ calculated for $C_{12}H_{16}BrO_3$ 287.0283, found 287.0280.

3-bromo-2-(5-chloro-2-methoxyphenyl)tetrahydrofuran (2o)

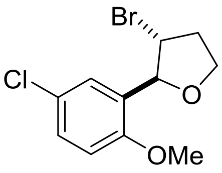

^{1}HNMR ($CDCl_3$, 400 MHz): δ 7.28 (d, J = 2.4 Hz, 1H), 7.21 (dd, J = 8.7, 2.6 Hz, 1H), 6.78 (d, J = 8.7 Hz, 1H), 5.46 (s, 1H), 4.39 (d, J = 5.4 Hz, 1H), 4.35 (td, J = 8.2, 1.9 Hz, 1H), 4.32-4.24 (m, 1H), 3.86 (s, 3H), 2.31 (ddd, J = 10.3, 8.3, 5.4 Hz, 1H), 2.22 (dd, J = 13.9, 5.6 Hz, 1H); ^{13}C NMR ($CDCl_3$, 100 MHz): δ 154.8, 131.4, 128.5, 126.2, 125.8, 111.4, 84.7, 67.9, 55.7, 52.8, 35.3. HRMS-ESI (*m/z*): $[M+H]^+$ calculated for $C_{11}H_{13}BrClO_2$ 290.9787, found 290.9785.

3-bromo-2-(2-methoxynaphthalen-1-yl)tetrahydrofuran (2p)

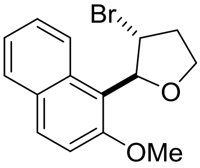

^{1}HNMR ($CDCl_3$, 400 MHz): δ 8.15 (d, J = 8.7 Hz, 1H), 7.81 (dd, J = 18.3, 8.7 Hz, 2H), 7.53-7.41 (m, 1H), 7.39-7.22 (m, 2H), 6.01 (d, J = 6.9 Hz, 1H), 4.84-4.73 (m, 1H), 4.39-4.27 (m, 1H), 4.21-4.09 (m, 1H), 3.96 (s, 3H), 2.99-2.81 (m, 1H), 2.57-2.44 (m, 1H); ^{13}C NMR ($CDCl_3$, 100 MHz): δ 156.3, 132.7, 130.8, 129.6, 128.8, 126.7, 123.5, 123.3, 118.1, 114.0, 83.0, 67.3, 57.0, 50.4, 38.2. HRMS-ESI (*m*/*z*): $[M+H]^+$ calculated for $C_{15}H_{16}BrO_2$ 307.0334, found 307.0330.

(4-(3-bromo-2-methyltetrahydrofuran-2-yl)phenoxy)(*tert*-butyl)diphenylsilane (2q)

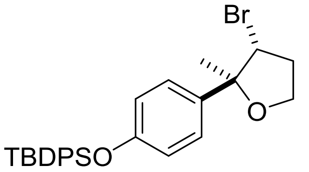

^{1}HNMR ($CDCl_3$, 400 MHz): δ 7.77-7.66 (m, 4H), 7.43 (dd, J = 8.4, 6.2 Hz, 2H), 7.36 (dd, J =11.0, 4.1 Hz, 4H), 7.14-7.06 (m, 2H), 6.75-6.68 (m, 2H), 4.50 (dd, J = 5.6, 3.2 Hz, 1H), 4.18 (dd, J = 15.8, 8.1 Hz, 1H), 3.98 (td, J = 8.4, 3.7 Hz, 1H), 2..37-2.30 (m, 1H), 2.26-2.20 (m, 1H), 1.6 (s, 3H), 1.1 (s, 9H); ^{13}C NMR ($CDCl_3$, 100 MHz): δ 154.7, 137.7, 135.5, 132.9, 129.9, 127.7, 125.5, 119.5, 86.4, 65.5, 58.7, 36.4, 28.0, 26.5, 19.4. HRMS-ESI (*m*/*z*): $[M+H]^+$ calculated for $C_{27}H_{32}BrO_2Si$ 495.1355, found 495.1358.

3-bromo-2,2-bis(4-methoxyphenyl)tetrahydrofuran (2r)

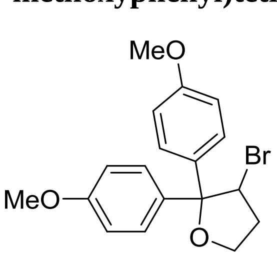

^{1}HNMR ($CDCl_3$, 400 MHz): δ 7.38 (dd, J = 20.5, 8.8 Hz, 4H), 6.82 (dd, J = 14.0, 8.8 Hz, 4H), 5.20 (dd, J = 4.5, 2.6 Hz, 1H), 4.39 (q, J = 8.0 Hz, 1H), 4.05 (td, J = 8.2, 4.4

Hz, 1H), 3.75 (d, *J* = 5.2 Hz, 6H), 2.45 (dd, *J* = 7.8, 4.6 Hz, 2H); ^{13}C NMR ($CDCl_3$, 100 MHz): δ 158.7, 158.3, 136.9, 136.3, 127.3, 126.4, 114.0, 113.0, 90.5, 65.2, 57.3, 55.3, 55.1, 36.58. HRMS-ESI (*m*/*z*): $[M+H]^+$ calculated for $C_{18}H_{20}BrO_3$ 363.0596, found 363.0598.

3-bromo-2-(4-methoxyphenyl)-4,4-dimethyltetrahydrofuran (2s)

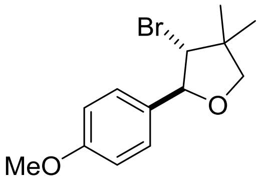

^{1}HNMR ($CDCl_3$, 400 MHz): δ 7.35 (d, *J* = 8.6 Hz, 2H), 6.9 (d, *J* = 8.7 Hz, 1H), 4.92 (d, *J* = 9.1 Hz, 1H), 3.92-3.85 (m, 2H), 3.81 (s, 3H), 3.77 (d, *J* = 9.1 Hz, 1H), 1.26 (s, 3H), 1.14 (s, 3H); ^{13}C NMR ($CDCl_3$, 100 MHz): δ 159.6, 131.8, 127.4, 113.8, 85.6, 78.7, 65.3, 55.2, 42.7, 23.9, 23.7. HRMS-ESI (*m*/*z*): $[M+H]^+$ calculated for $C_{13}H_{18}BrO_2$ 285.0490, found 285.0492.

3-bromo-2-(4-methoxyphenyl)tetrahydro-2*H*-pyran (2t)

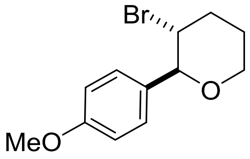

^{1}HNMR ($CDCl_3$, 400 MHz): δ 7.31 (d, *J* = 8.7 Hz, 2H), 6.89 (d, *J* = 8.7 Hz, 2H), 4.28 (d, *J* = 10 Hz, 1H), 4.14-4.01 (m, 2H), 3.80 (s, 3H), 3.63 (td, *J* =12.0 , 2.2 Hz, 1H), 2.61-2.56 (m, 1H), 217-2.06 (m, 1H), 1.99-1.87 (m, 1H), 1.75-1.71 (m, 1H); ^{13}C NMR ($CDCl_3$, 100 MHz): δ 159.6, 132.0, 128.7, 113.6, 85.1, 68.7, 55.2, 52.7, 36.2, 28.4. HRMS-ESI (*m*/*z*): $[M+H]^+$ calculated for $C_{12}H_{16}BrO_2$ 271.0334, found 271.0330.

3-bromo-2-p-tolyltetrahydro-2*H*-pyran (2u)

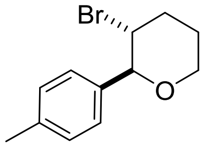

^{1}HNMR ($CDCl_3$, 400 MHz): δ 7.27 (d, *J* = 8.4 Hz, 2H), 7.17 (d, *J* = 7.9 Hz, 2H), 4.29 (d, *J* = 10.0 Hz, 1H), 4.14-4.03 (m, 2H), 3.63 (td, *J* = 12.0, 2.2 Hz, 1H), 2.61-2.55 (m, 1H), 2.35 (s, 3H), 2.12 (ddd, *J* = 24.8, 13.0, 4.2 Hz, 1H), 2.06-1.84 (m, 1H), 1.74 (dd,

J = 13.7, 2.3 Hz, 1H); ^{13}C NMR ($CDCl_3$, 100 MHz): δ 138.2, 136.7, 129.0, 127.4, 85.4, 68.7, 52.5, 36.2, 28.4, 21.3. Data are consistent with literature values.[9]

3-bromo-2-(4-methoxyphenyl)-6-methyltetrahydro-2*H*-pyran (2v)

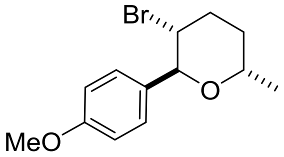

^{1}HNMR ($CDCl_3$, 400 MHz): δ 7.30 (d, J = 8.7 Hz, 2H), 6.89 (d, J = 8.7 Hz, 2H), 4.73 (d, J = 8.4 Hz, 1H), 4.23 (ddd, J = 14.3, 9.5, 5.1 Hz, 2H), 3.80 (s, 3H), 2.31 (d, J = 5.2 Hz, 2H), 2.04-1.91 (m, 1H), 1.75-1.64 (m, 1H), 1.37 (d, J = 6.7 Hz, 3H); ^{13}C NMR ($CDCl_3$, 100 MHz): δ 159.4, 132.0, 128.5, 113.8, 68.8, 55.3, 53.0, 30.9, 30.3, 17.4. HRMS-ESI (m/z): $[M+H]^+$ calculated for $C_{13}H_{18}BrO_2$ 285.0490, found 285.0492.

2-(bromo(4-methoxyphenyl)methyl)-5-methyltetrahydrofuran (3v)

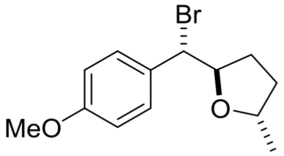

^{1}HNMR ($CDCl_3$, 400 MHz): δ 7.31 (d, J = 8.1 Hz, 2H), 6.88 (d, J = 8.1 Hz, 2H), 4.34 (d, J = 10.0 Hz, 1H), 4.07-3.91 (m, 1H), 3.80 (s, 3H), 3.72 (dd, J = 10.6, 5.7 Hz, 1H), 2.54 (d, J = 13.4 Hz, 1H), 2.15 (qd, J = 13.0, 3.6 Hz, 1H), 1.77 (d, J = 13.3 Hz, 1H), 1.58 (d, J = 12.0 Hz, 1H), 1.21 (d, J = 6.0 Hz, 3H); ^{13}C NMR ($CDCl_3$, 100 MHz): δ 159.5, 132.2, 128.7, 113.6, 84.7, 74.6, 55.2, 53.0, 36.2, 35.5, 21.6. HRMS-ESI (m/z): $[M+H]^+$ calculated for $C_{13}H_{18}BrO_2$ 285.0490, found 285.0492.

3-bromo-2-(4-methoxyphenyl)-6-phenyltetrahydro-2*H*-pyran (2w)

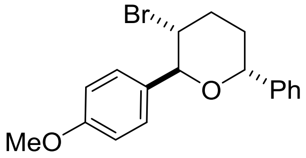

^{1}HNMR ($CDCl_3$, 400 MHz): δ 7.51-7.38 (m, 4H), 7.33 (dd, J = 8.4, 5.3 Hz, 3H), 6.95-6.88 (m, 2H), 5.10 (s, 1H), 4.64 (d, J = 8.2 Hz, 1H), 4.42 (td, J = 9.0, 4.1 Hz, 1H), 3.82 (s, 3H), 2.44 (ddd, J = 15.7, 10.1, 5.0 Hz, 2H), 2.33-2.12 (m, 2H); ^{13}C

9 S. E. Denmark, M. T. Burk, Org. Lett., 2012, 14, 256-259.

NMR ($CDCl_3$, 100 MHz): δ 159.5, 139.8, 131.6, 128.8, 128.5, 127.4, 126.6, 113.9, 77.8, 73.5, 55.3, 52.3, 30.9, 28.4. HRMS-ESI (*m*/*z*): $[M+H]^+$ calculated for $C_{18}H_{20}BrO_2$ 347.0647, found 347.0648.

2-(bromo(4-methoxyphenyl)methyl)-5-phenyltetrahydrofuran (3w)

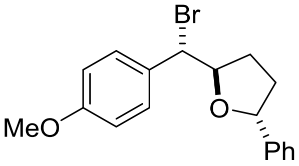

^{1}HNMR ($CDCl_3$, 400 MHz): δ 7.32 (ddd, *J* = 27.5, 18.9, 7.3 Hz, 8H), 6.89 (d, *J* = 8.7 Hz, 2H), 4.65 (dd, *J* = 11.2, 2.0 Hz, 1H), 4.53 (d, *J* = 10.0 Hz, 1H), 4.14- 4.02 (m, 1H), 3.80 (s, 3H), 2.66 (dd, *J* = 13.2, 3.7 Hz, 1H), 2.33 (ddd, *J* = 25.3, 13.1, 4.2 Hz, 1H), 2.14-2.00 (m, 1H), 1.90 (ddd, *J* = 25.0, 13.6, 3.8 Hz, 1H); ^{13}C NMR ($CDCl_3$, 100 MHz): δ^{13}C NMR (101 MHz, $CDCl_3$) δ 159.6, 141.8, 132.0, 128.83 (s), 128.3, 127.6, 125.8, 113.5, 85.1, 80.2, 55.2, 52.8, 36.6, 36.1. HRMS-ESI (*m*/*z*): $[M+H]^+$ calculated for $C_{18}H_{20}BrO_2$ 347.0647, found 347.0648.

2-(bromomethyl)-5-phenyltetrahydrofuran (3x)

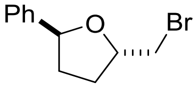

^{1}HNMR ($CDCl_3$, 400 MHz): δ 7.40-7.30 (m, 4H), 5.15-5.05 (m, 1H), 4.58-4.44 (m, 1H), 3.55 (dd, *J*= 10.1, 4.7 Hz, 1H), 3.46 (dd, *J*= 10.1, 6.8 Hz, 1H), 2.41 (dt, *J*= 11.3, 5.6 Hz, 1H), 2.31-2.14 (m, 1H), 2.02-1.76 (m, 2H); ^{13}C NMR ($CDCl_3$, 100 MHz): δ 142.7, 128.4, 127.4, 125.6, 81.54 (s), 78.7, 36.0, 35.2, 31.2. Data are consistent with literature values.[10]

[10] M. Greb, J. Hartung, F. Köhler, K. Spehar, R. Kluge, R. Csuk, *Eur. J. Org. Chem.* **2004**, 3799-3812.

6. ^{1}H and ^{13}C NMR spectra

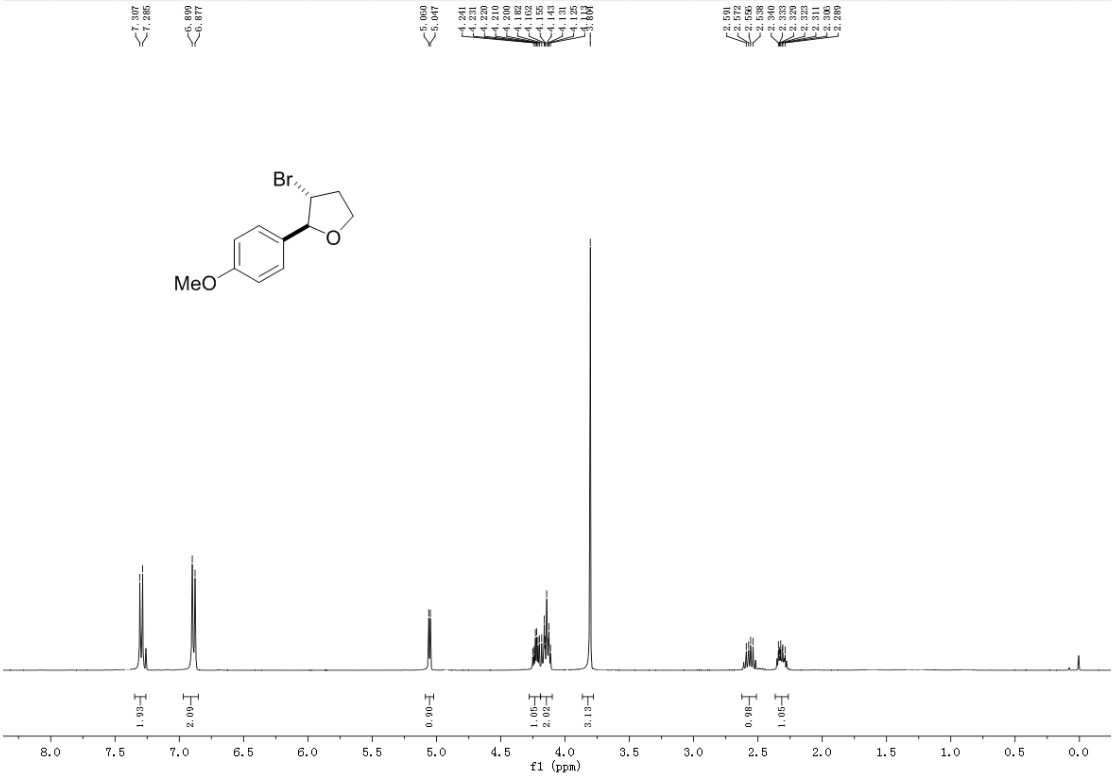

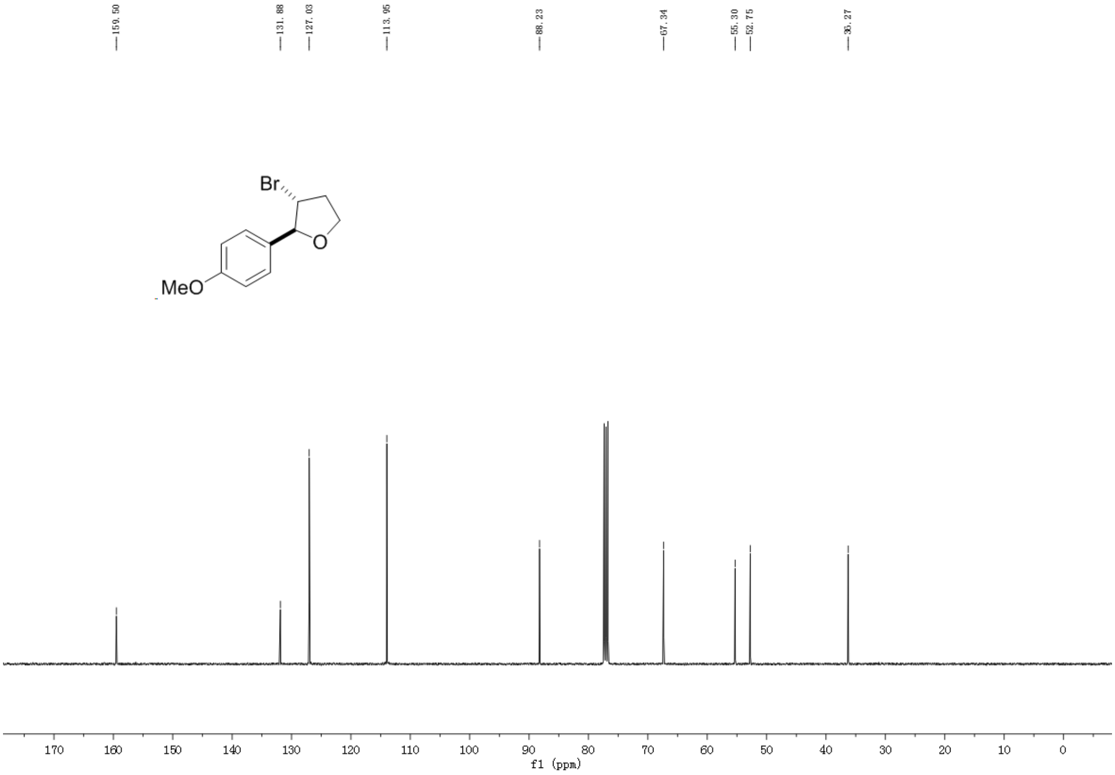

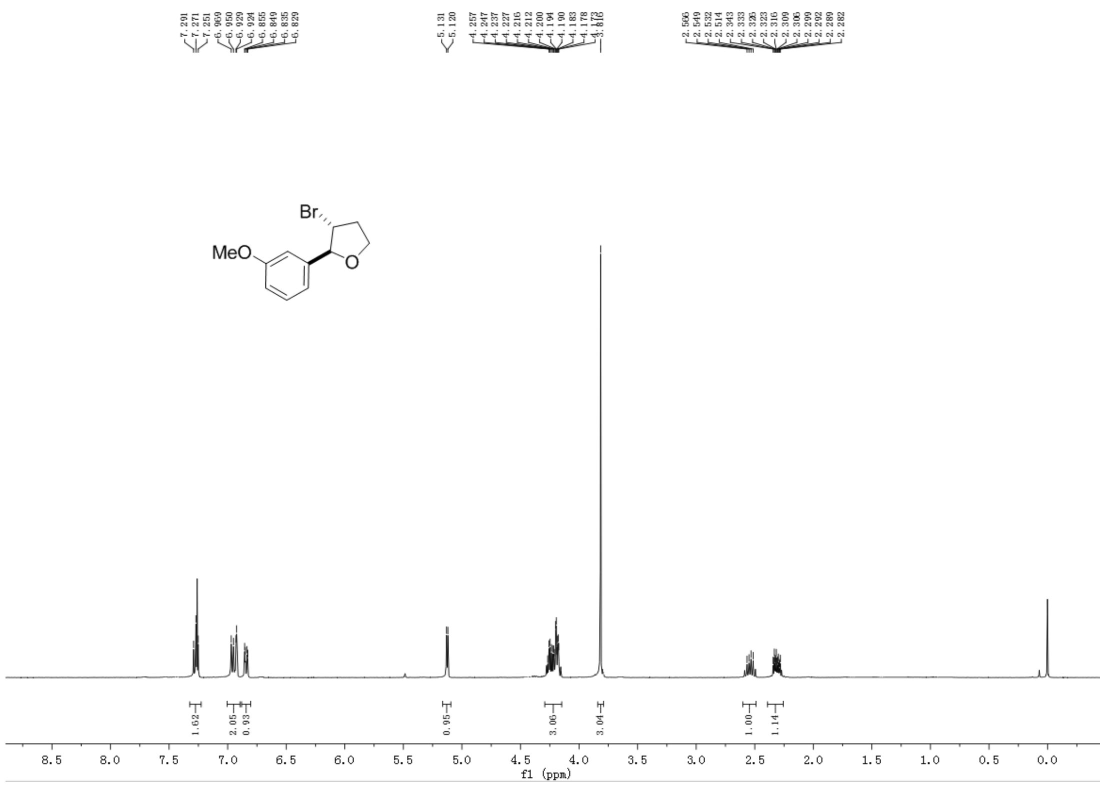
Br
MeO
O
f1 (ppm)

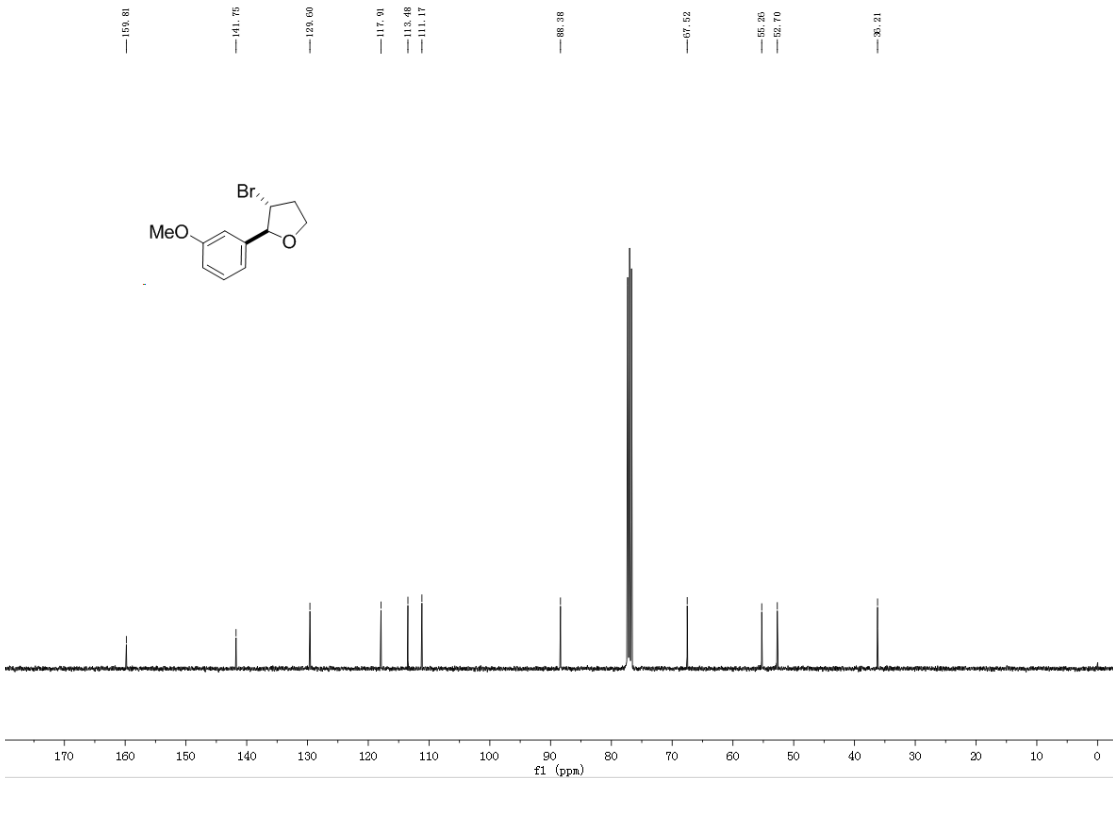
159.81
141.75
129.60
117.91
113.48
111.17
88.38
67.52
55.26
52.70
36.21
Br
MeO
O
f1 (ppm)

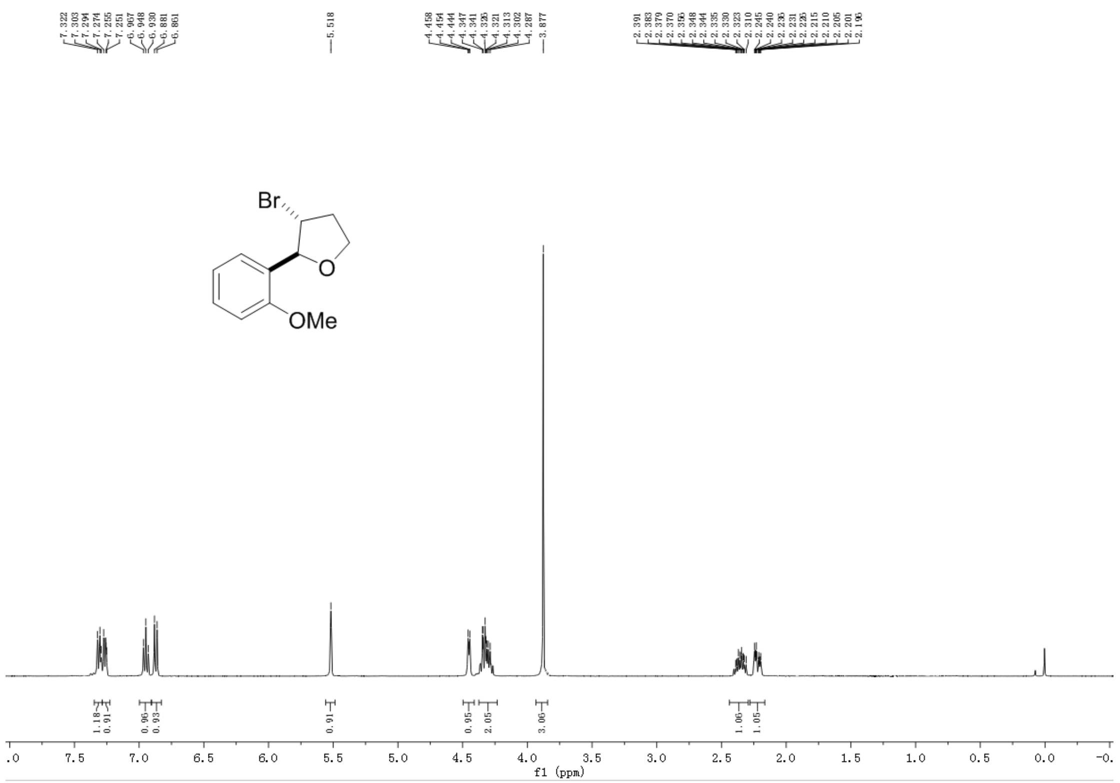
7.322
7.303
7.294
7.274
7.255
7.251
6.967
6.948
6.930
6.881
6.861
5.518
4.458
4.454
4.444
4.347
4.341
4.326
4.321
4.313
4.302
4.287
3.877
2.391
2.383
2.379
2.370
2.356
2.348
2.344
2.335
2.330
2.323
2.310
2.245
2.240
2.236
2.231
2.226
2.215
2.210
2.205
2.201
2.196
Br
O
OMe
1.18
0.91
0.96
0.93
0.91
0.95
2.05
3.06
1.06
1.05
f1 (ppm)

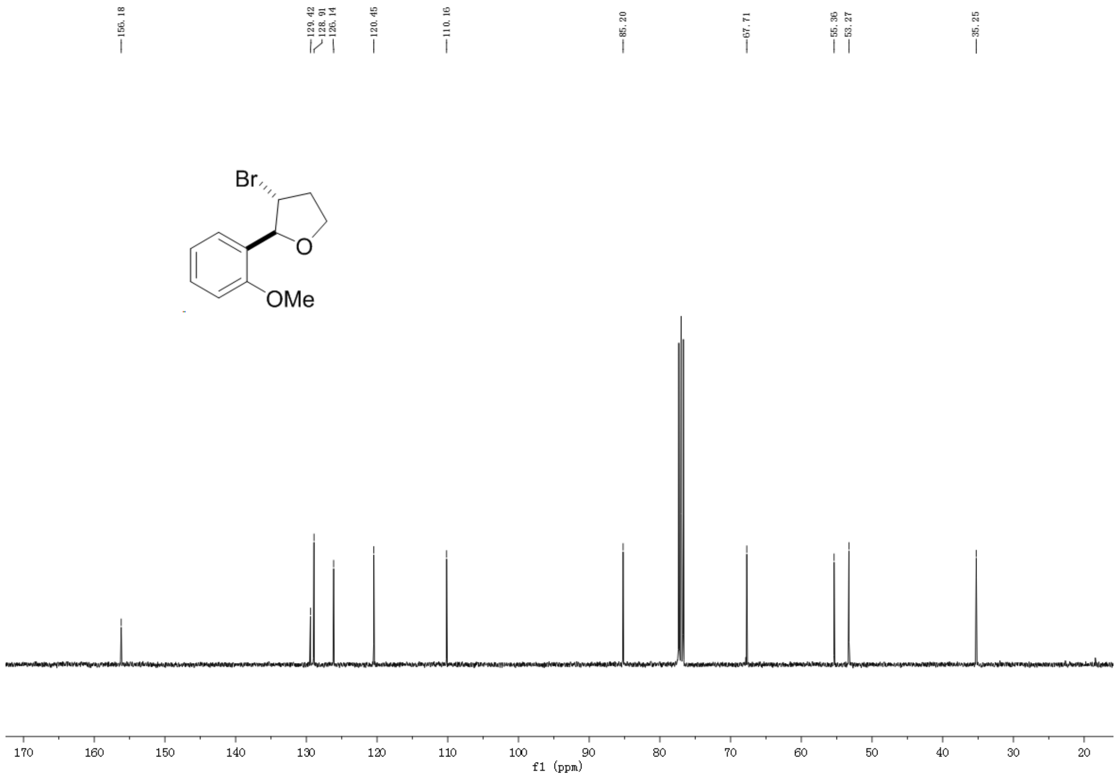
156.18
129.42
128.91
126.14
120.45
110.16
85.20
67.71
55.36
53.27
35.25
Br
O
OMe
f1 (ppm)

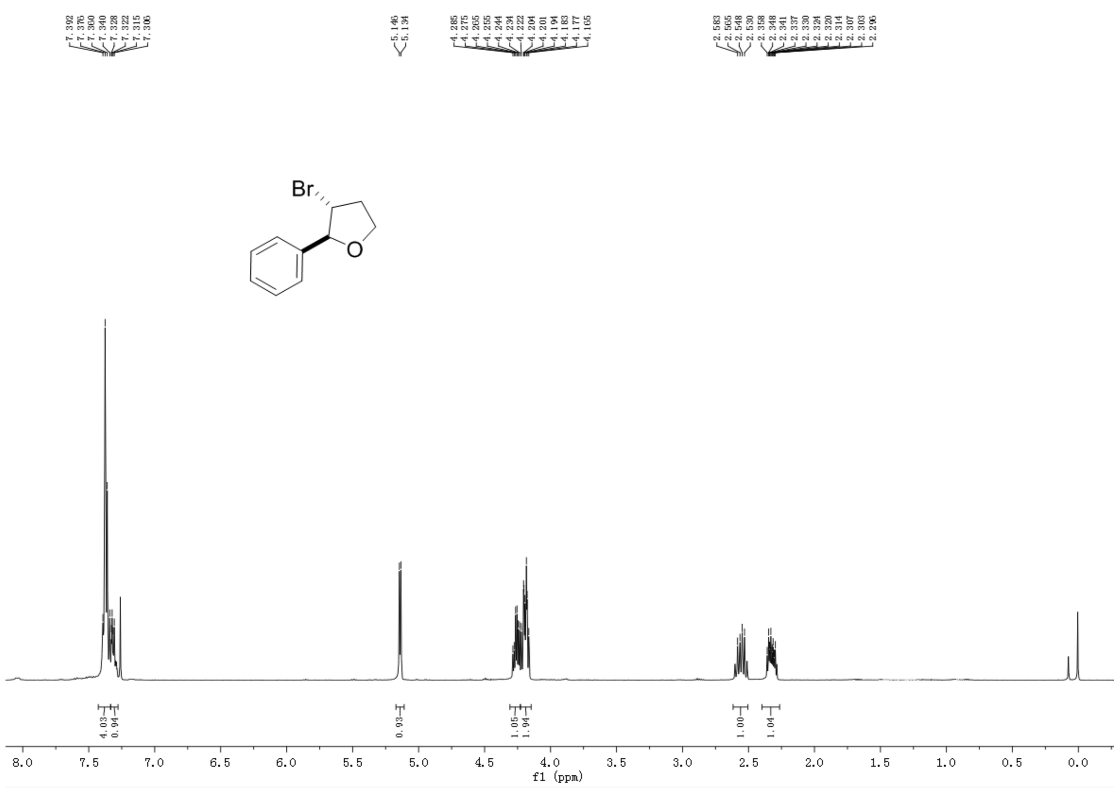

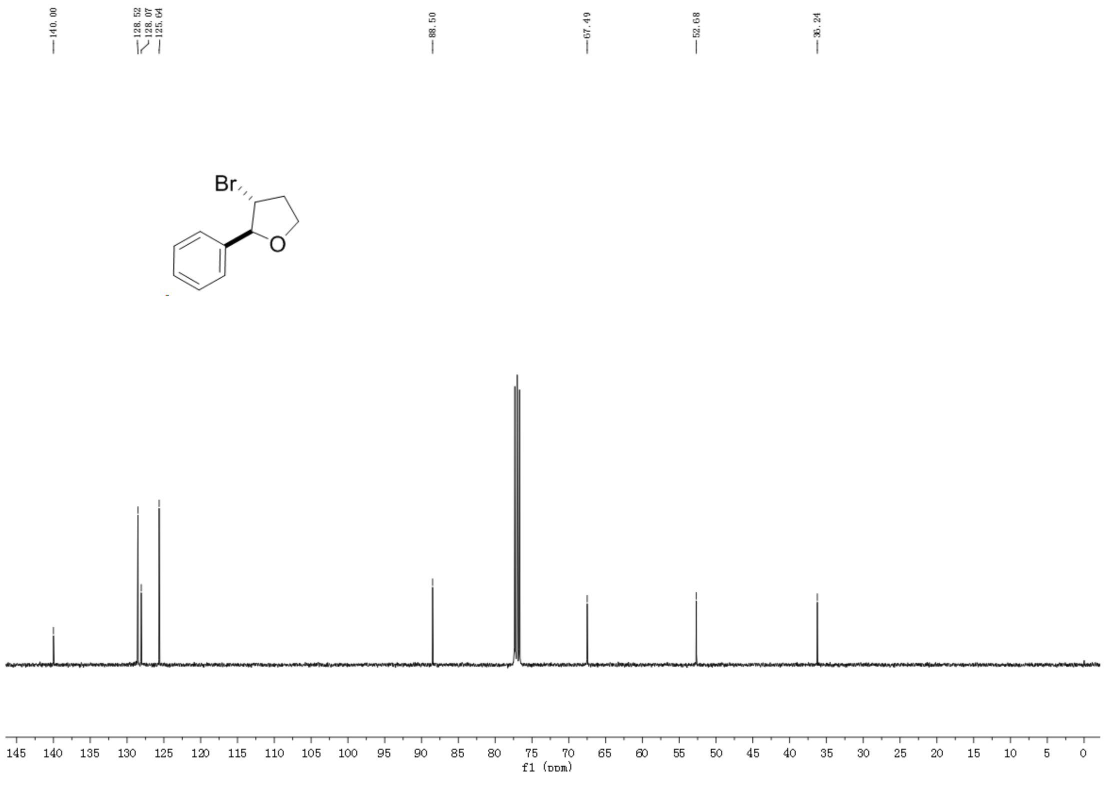

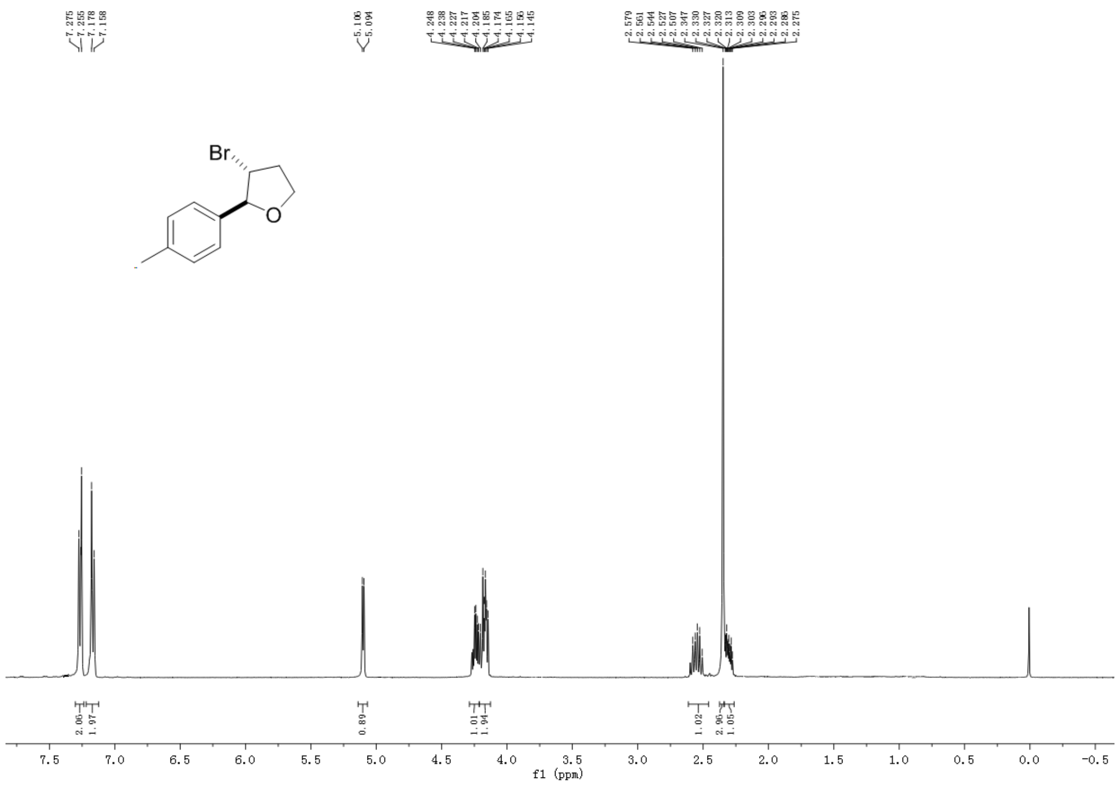

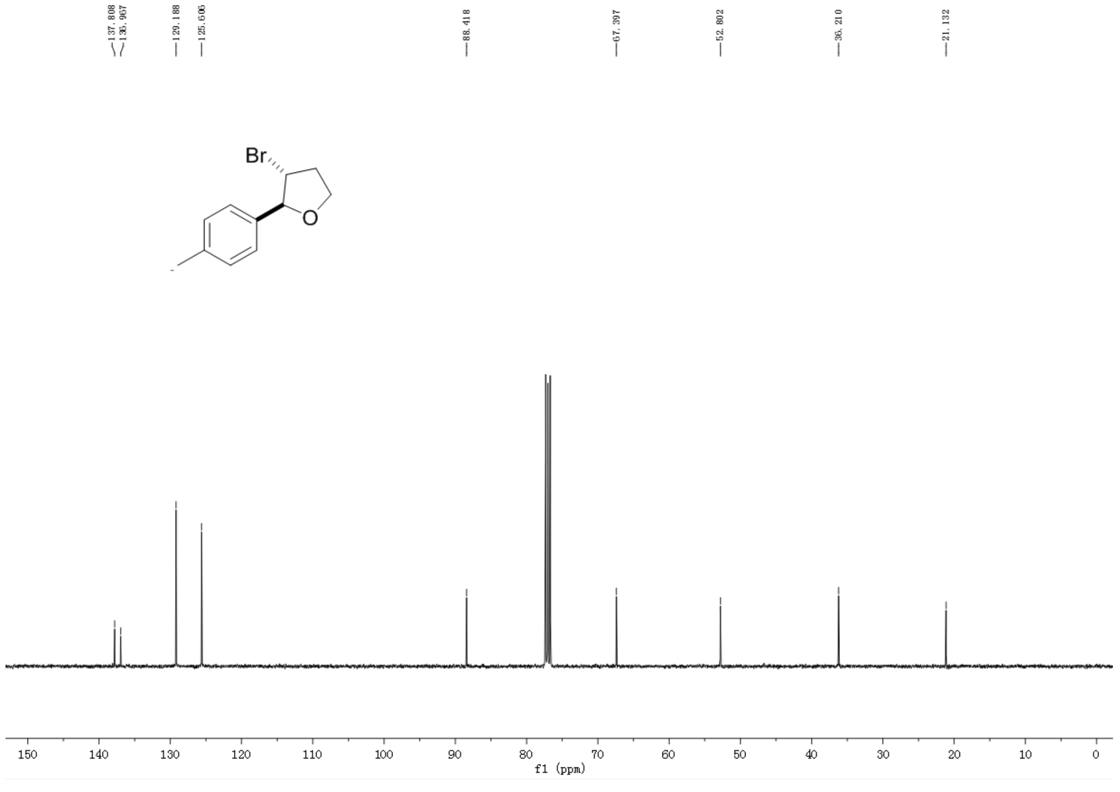

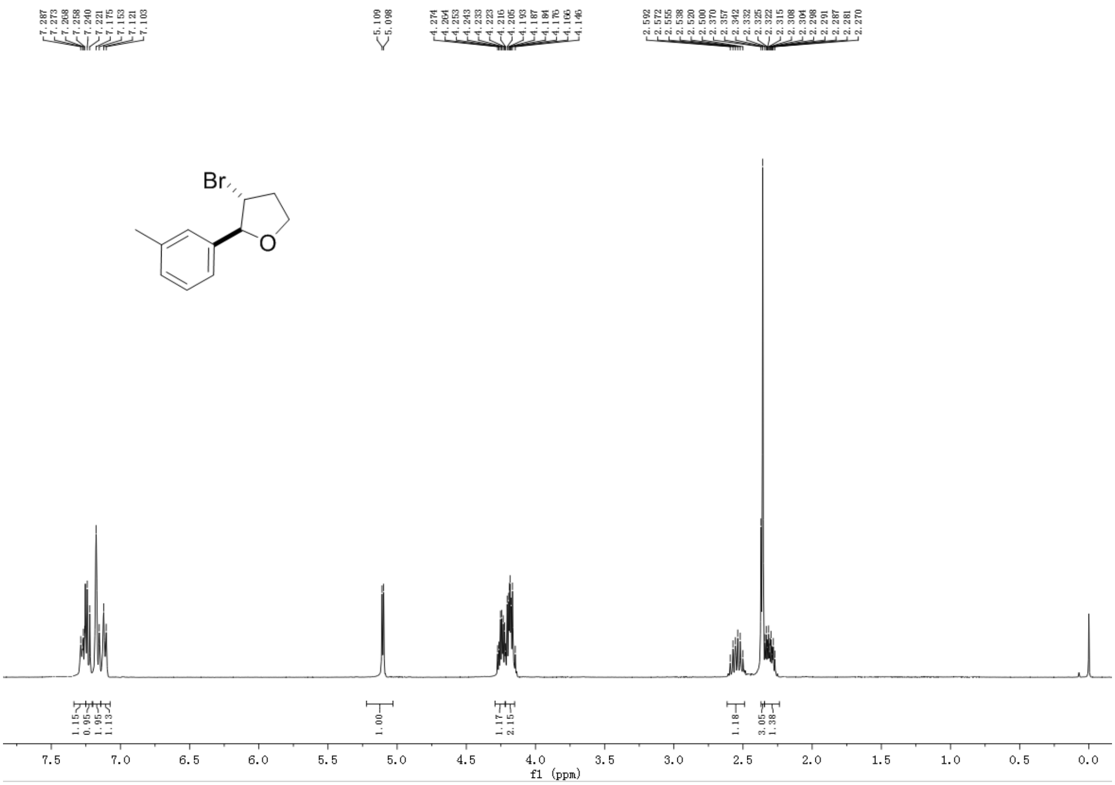
7.287 7.273 7.268 7.258 7.240 7.221 7.175 7.153 7.121 7.103
5.109 5.098
4.274 4.264 4.253 4.243 4.233 4.223 4.216 4.205 4.193 4.187 4.184 4.176 4.166 4.146
2.592 2.572 2.555 2.538 2.520 2.500 2.370 2.357 2.342 2.332 2.325 2.322 2.315 2.308 2.304 2.298 2.291 2.287 2.281 2.270
Br
O
1.15 0.95 1.95 1.13
1.00
1.17 2.15
1.18
3.05 1.38
7.5 7.0 6.5 6.0 5.5 5.0 4.5 4.0 3.5 3.0 2.5 2.0 1.5 1.0 0.5 0.0
f1 (ppm)

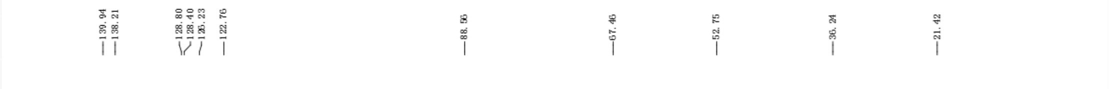
139.94 138.21
128.80 128.40 126.23 122.76
88.56
67.46
52.75
36.24
21.42
Br
O
150 140 130 120 110 100 90 80 70 60 50 40 30 20 10 0
f1 (ppm)

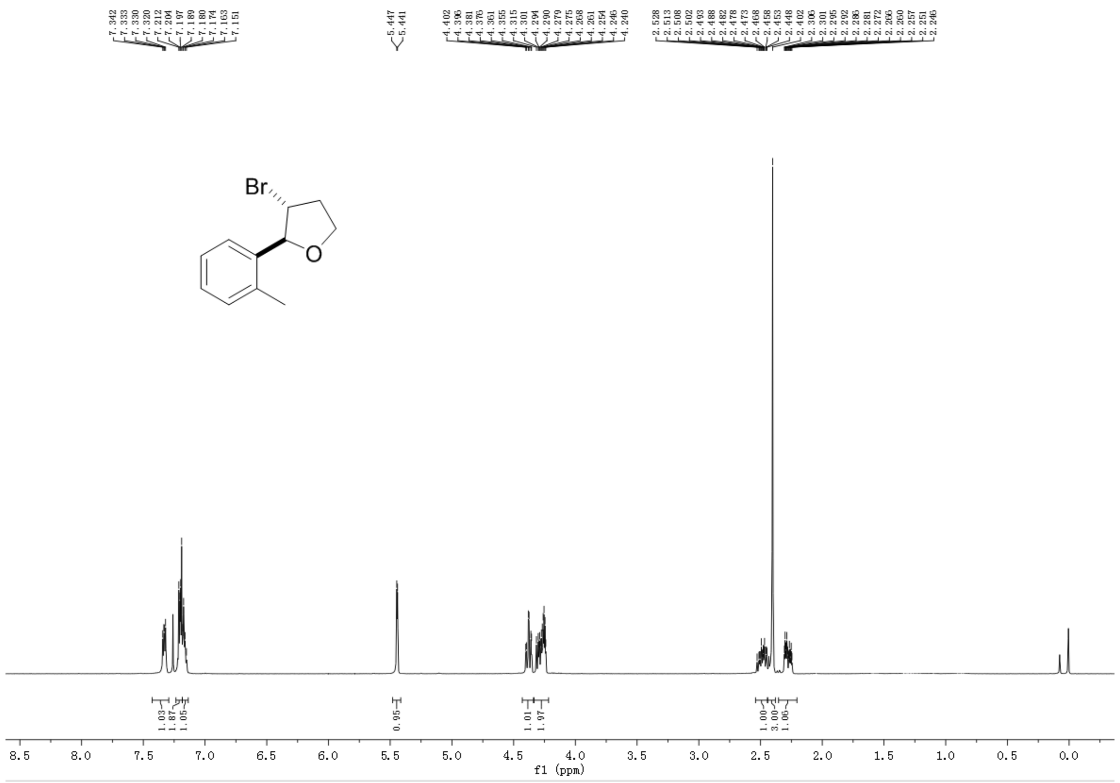
7.342
7.333
7.330
7.320
7.212
7.204
7.197
7.189
7.180
7.174
7.163
7.151
5.447
5.441
4.402
4.396
4.381
4.376
4.361
4.355
4.315
4.301
4.294
4.290
4.279
4.275
4.268
4.261
4.254
4.246
4.240
2.528
2.513
2.508
2.502
2.493
2.488
2.482
2.478
2.473
2.468
2.458
2.453
2.448
2.402
2.306
2.301
2.295
2.292
2.286
2.281
2.272
2.266
2.260
2.257
2.251
2.246
Br
O
1.03
1.87
1.05
0.95
1.01
1.97
1.00
3.00
1.06
8.5 8.0 7.5 7.0 6.5 6.0 5.5 5.0 4.5 4.0 3.5 3.0 2.5 2.0 1.5 1.0 0.5 0.0
f1 (ppm)

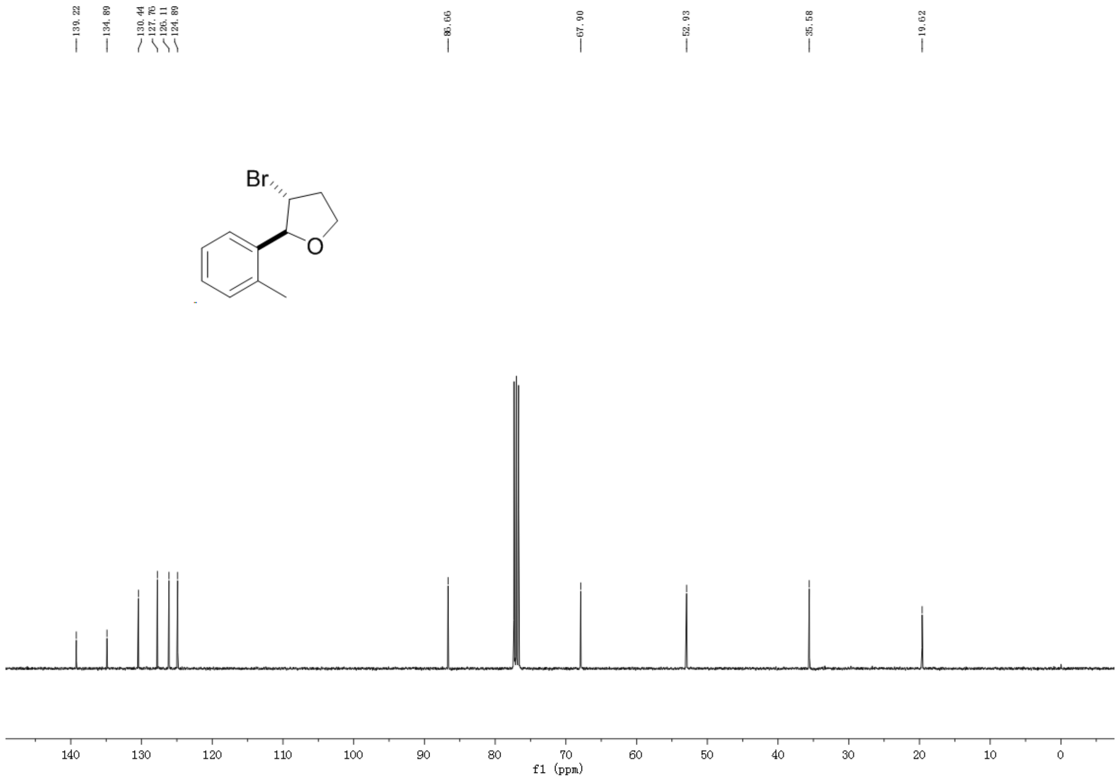
139.22
134.89
130.44
127.76
126.11
124.89
86.66
67.90
52.93
35.58
19.62
Br
O
140 130 120 110 100 90 80 70 60 50 40 30 20 10 0
f1 (ppm)

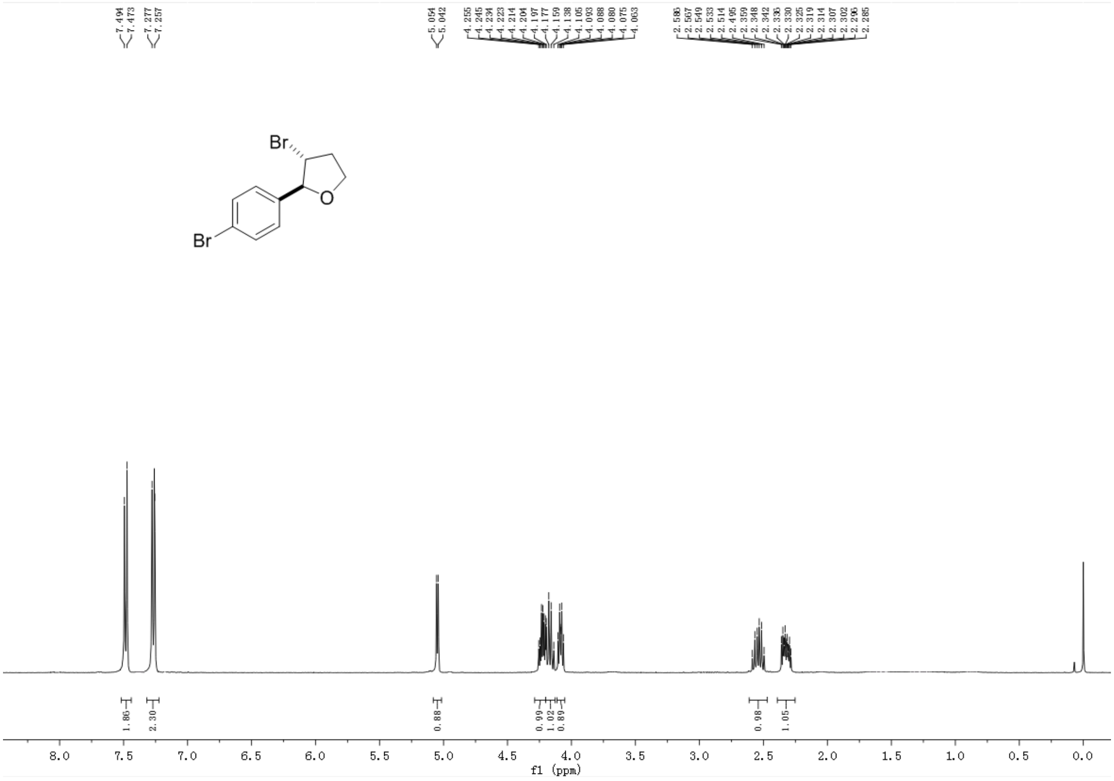

7.494
7.473
7.277
7.257
5.054
5.042
4.255
4.245
4.234
4.223
4.214
4.204
4.197
4.177
4.159
4.138
4.105
4.093
4.088
4.080
4.075
4.063
2.586
2.567
2.549
2.533
2.514
2.495
2.359
2.348
2.342
2.336
2.330
2.325
2.319
2.314
2.307
2.302
2.296
2.285
Br
O
Br
1.86
2.30
0.88
0.99
1.02
0.89
0.98
1.05
8.0
7.5
7.0
6.5
6.0
5.5
5.0
4.5
4.0
3.5
3.0
2.5
2.0
1.5
1.0
0.5
0.0
f1 (ppm)

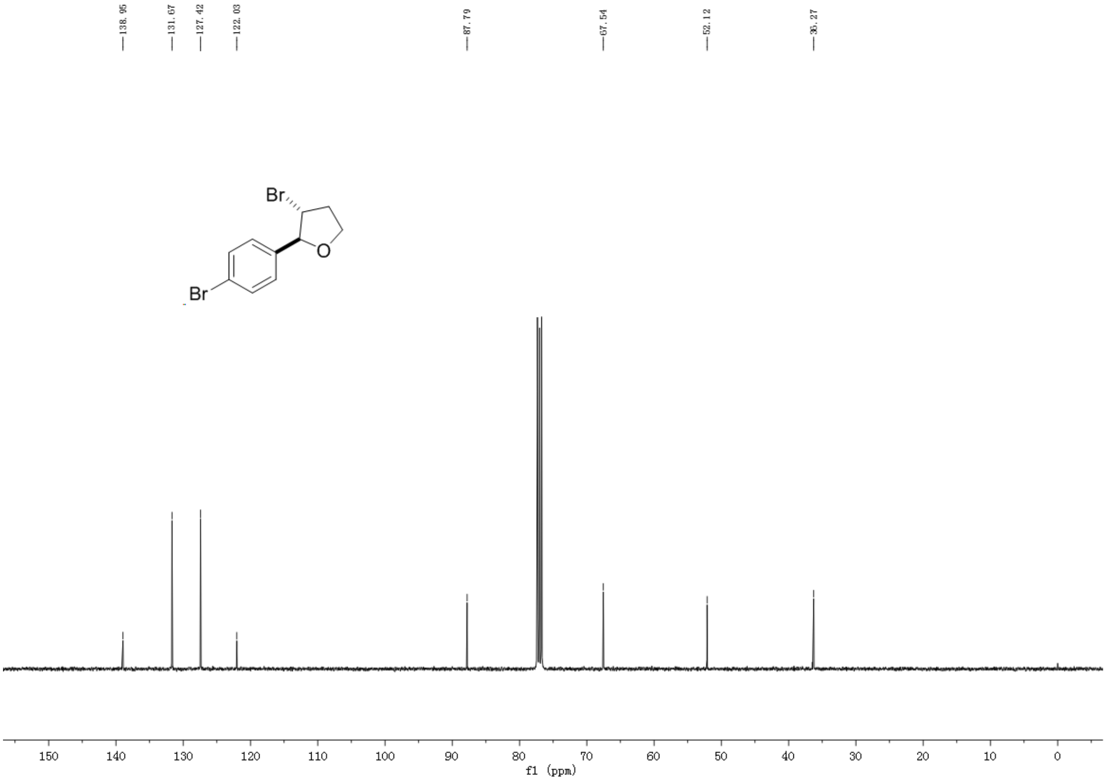

138.95
131.67
127.42
122.03
87.79
67.54
52.12
36.27
Br
O
Br
150
140
130
120
110
100
90
80
70
60
50
40
30
20
10
0
f1 (ppm)

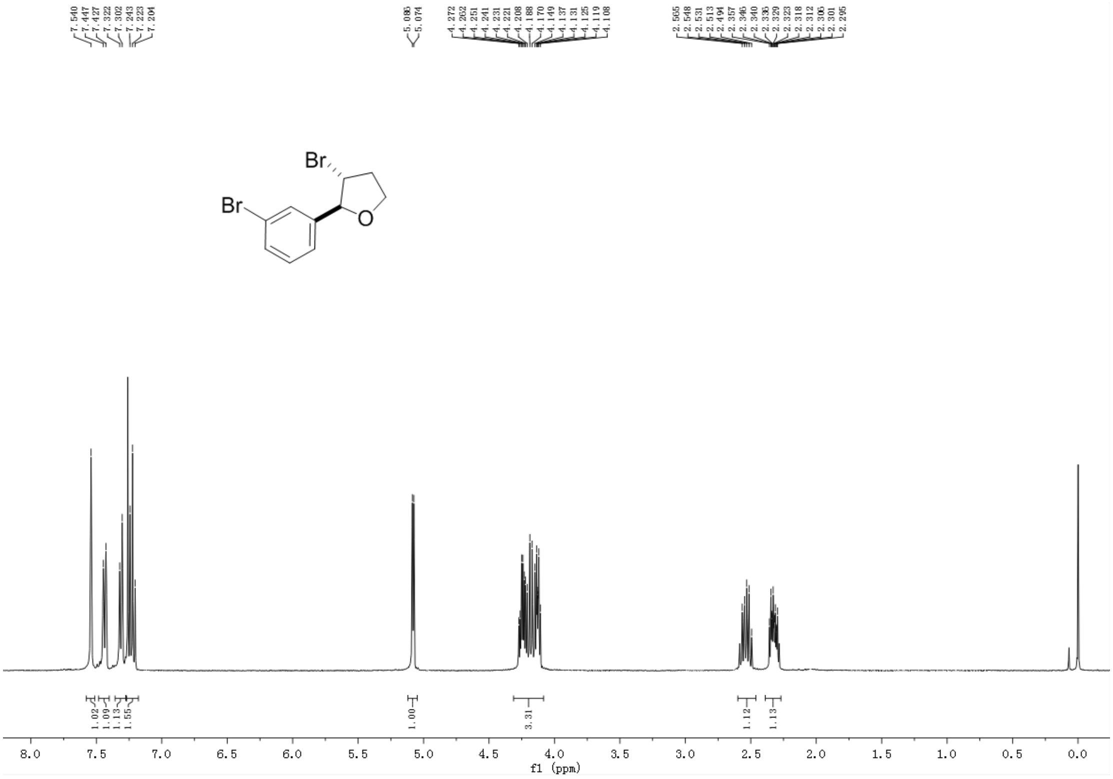
7.540
7.447
7.427
7.322
7.302
7.243
7.223
7.204
5.086
5.074
4.272
4.262
4.251
4.241
4.231
4.221
4.208
4.188
4.170
4.149
4.137
4.131
4.125
4.119
4.108
2.565
2.548
2.531
2.513
2.494
2.357
2.346
2.340
2.335
2.329
2.323
2.318
2.312
2.306
2.301
2.295
Br
Br
O
1.02
1.09
1.13
1.55
1.00
3.31
1.12
1.13
f1 (ppm)

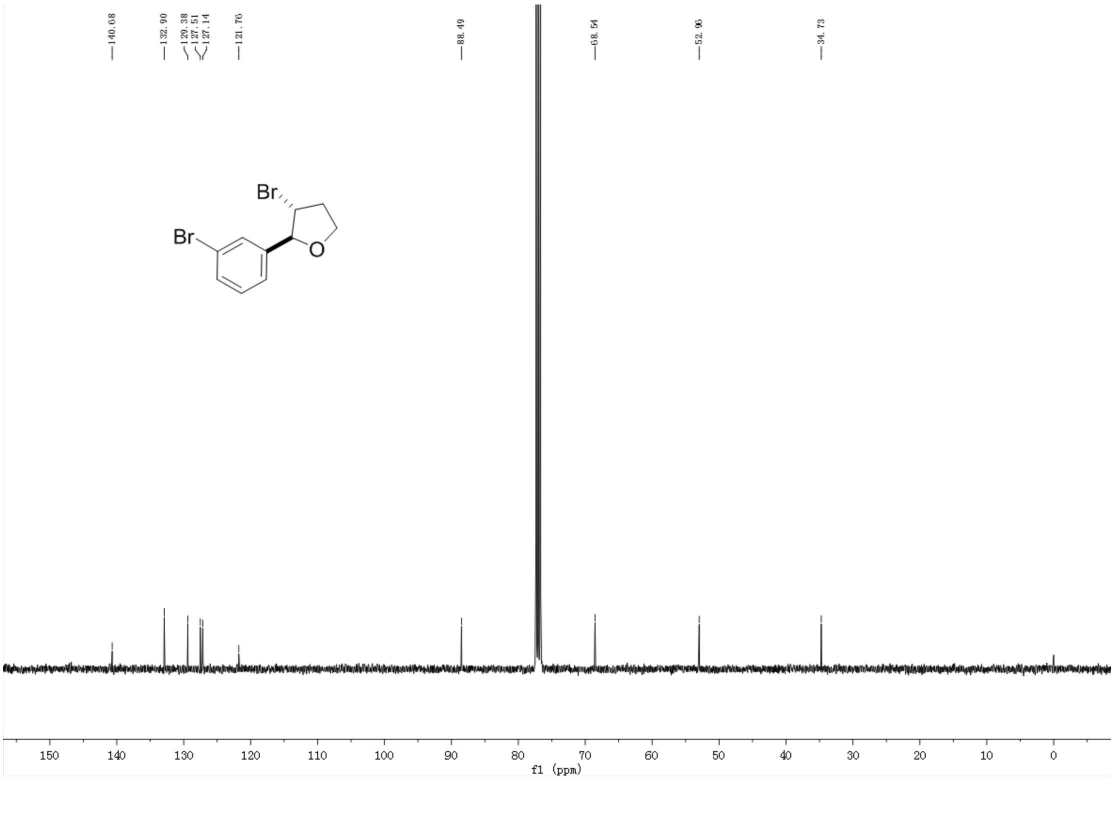
140.68
132.90
129.38
127.51
127.14
121.76
88.49
68.54
52.96
34.73
Br
Br
O
f1 (ppm)

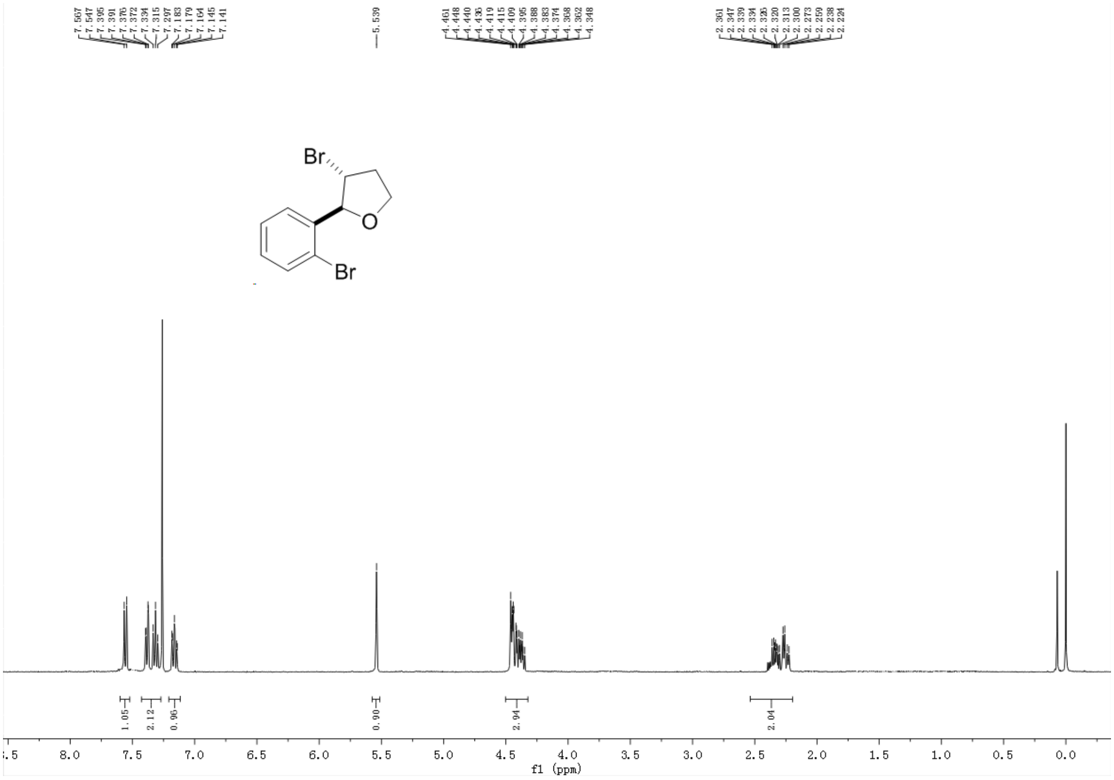
7.567
7.547
7.395
7.391
7.376
7.372
7.334
7.315
7.297
7.183
7.179
7.164
7.145
7.141
5.539
4.461
4.448
4.440
4.436
4.419
4.415
4.409
4.395
4.388
4.383
4.374
4.368
4.362
4.348
2.361
2.347
2.339
2.334
2.326
2.320
2.313
2.300
2.273
2.259
2.238
2.224
Br
O
Br
1.05
2.12
0.96
0.90
2.94
2.04
f1 (ppm)

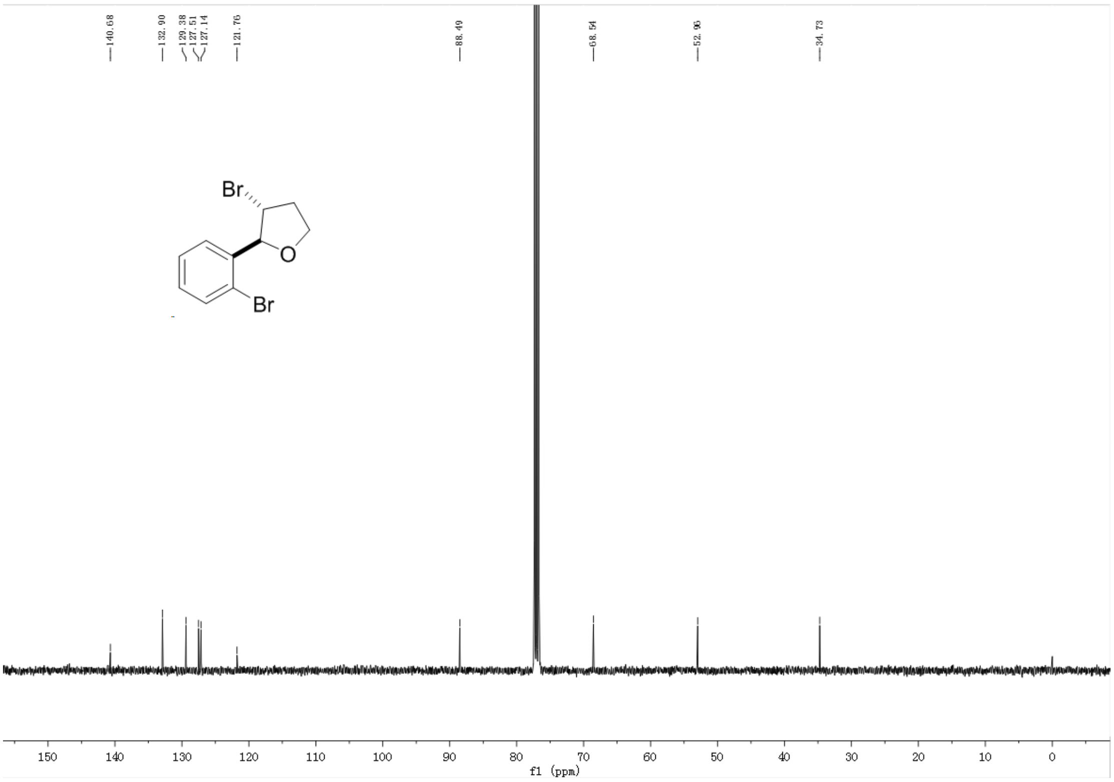
140.68
132.90
129.38
127.51
127.14
121.76
88.49
68.54
52.96
34.73
Br
O
Br
f1 (ppm)

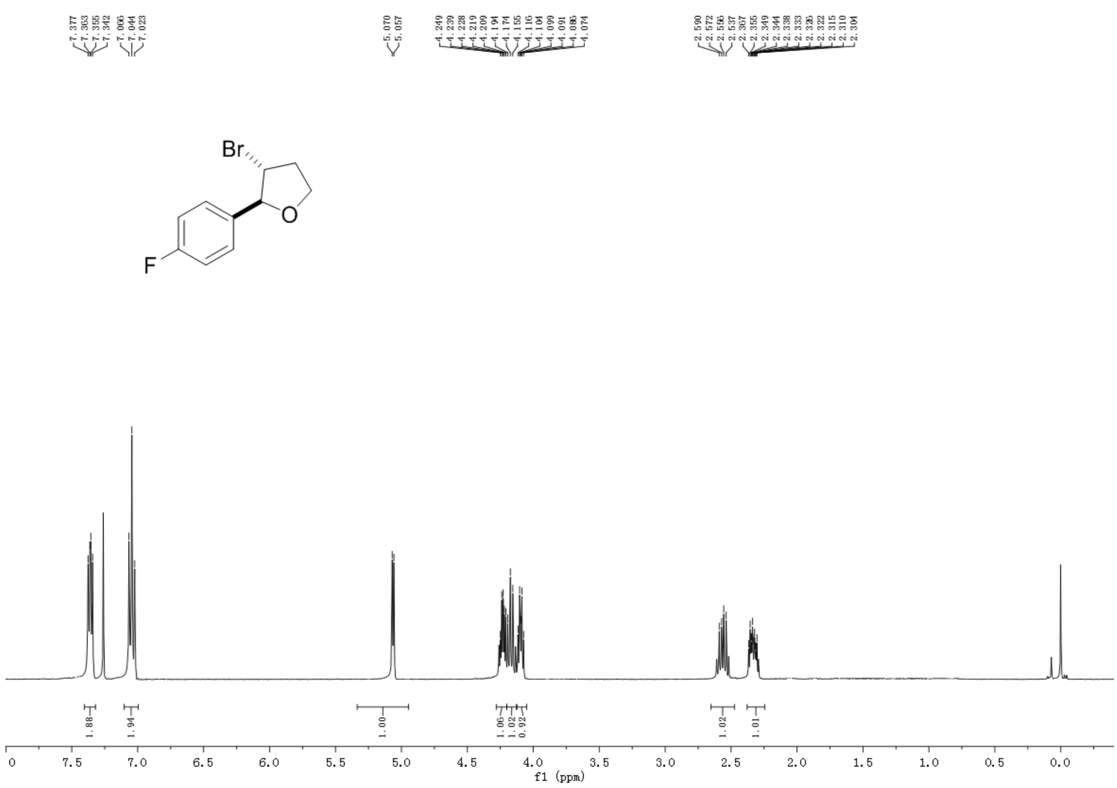

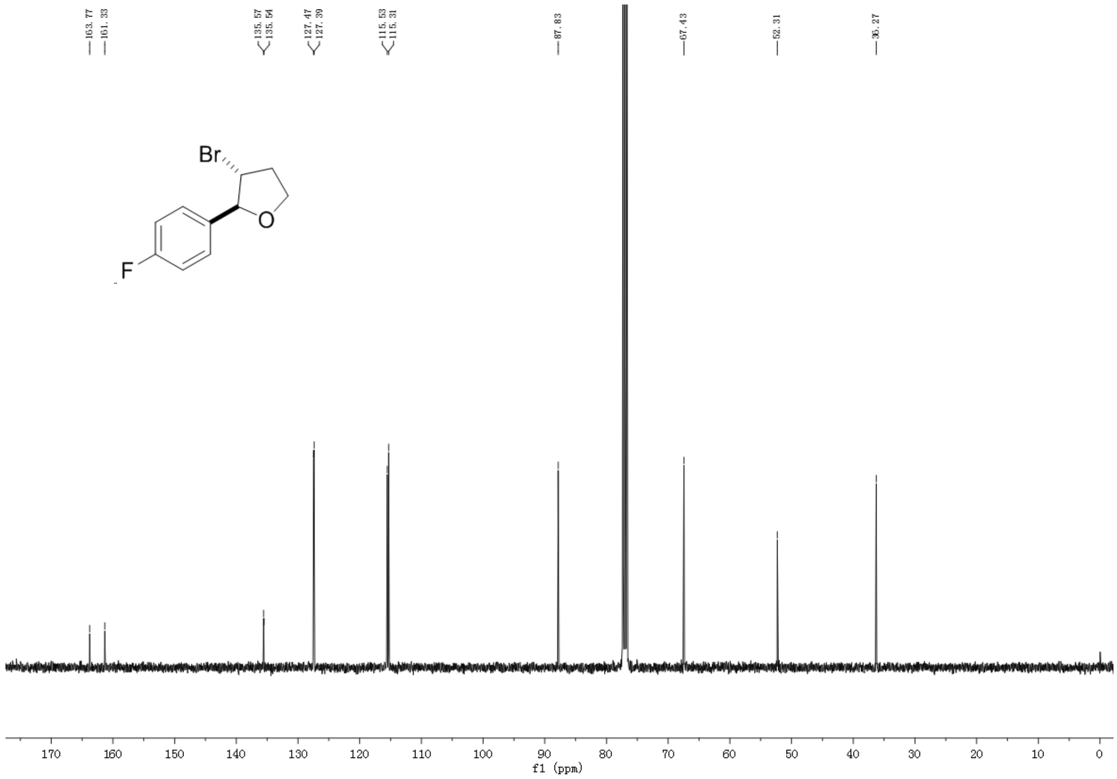

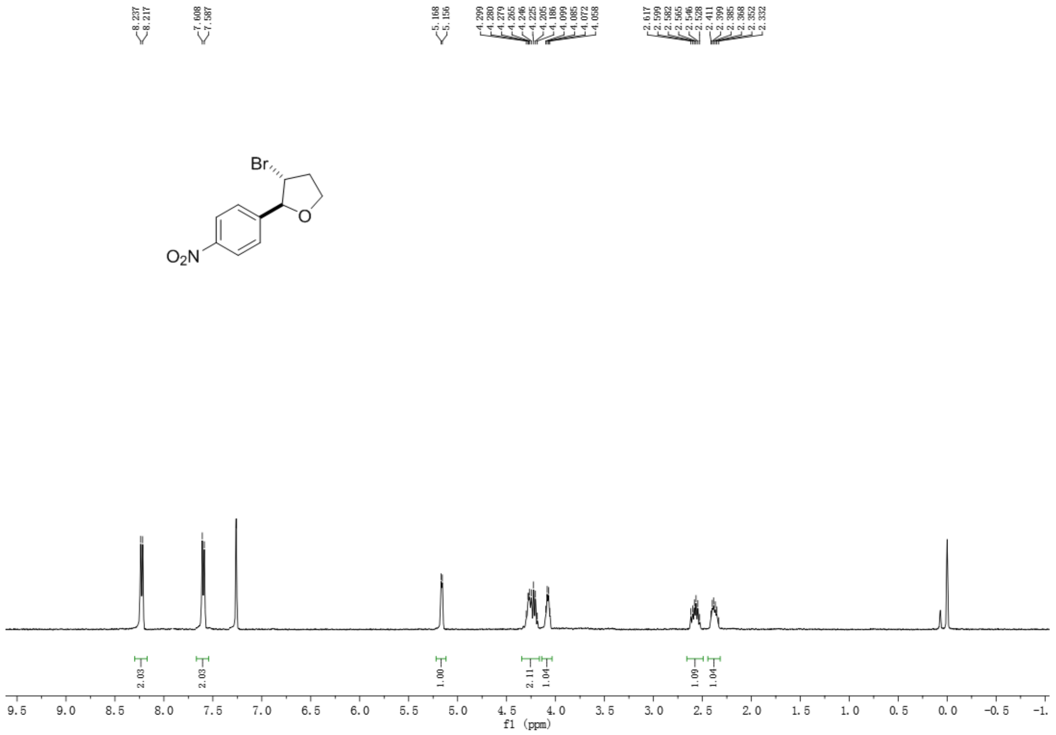

Br
O
O2N
f1 (ppm)

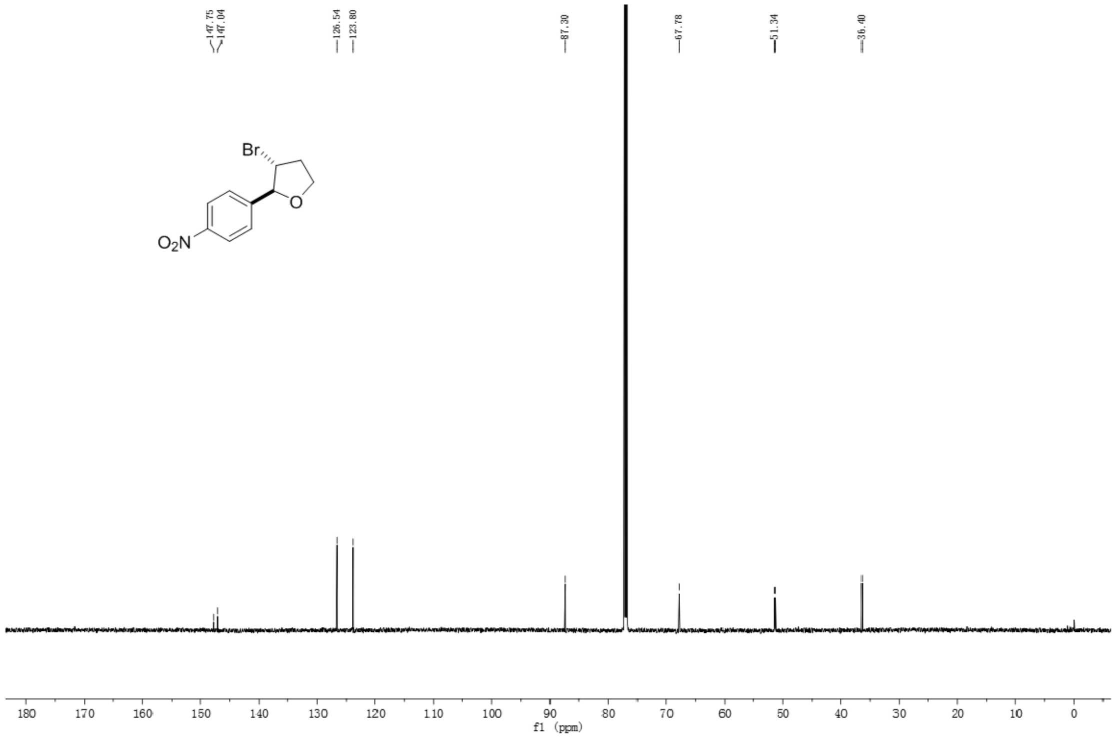

147.75
147.04
126.54
123.80
87.30
67.78
51.34
36.40
Br
O
O2N
f1 (ppm)

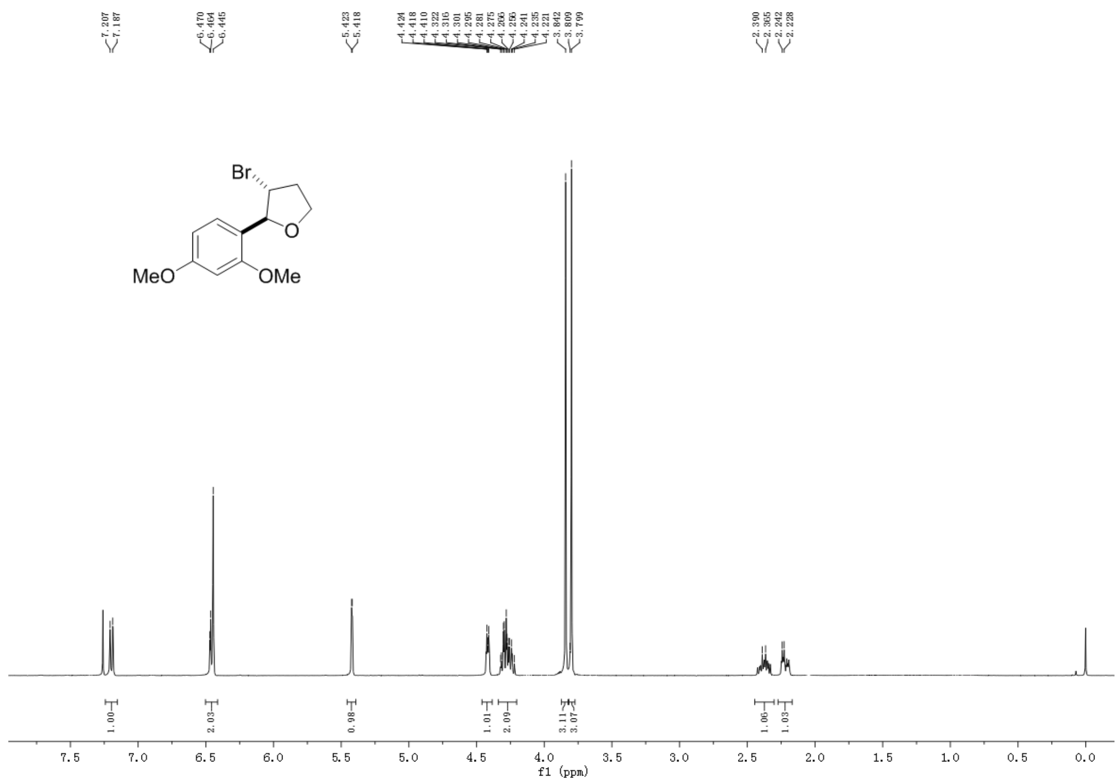
7.207
7.187
6.470
6.464
6.445
5.423
5.418
4.424
4.418
4.410
4.322
4.316
4.301
4.295
4.281
4.275
4.266
4.256
4.241
4.235
4.221
3.842
3.809
3.799
2.390
2.365
2.242
2.228
Br
O
MeO
OMe
1.00
2.03
0.98
1.01
2.09
3.11
3.07
1.06
1.03
f1 (ppm)

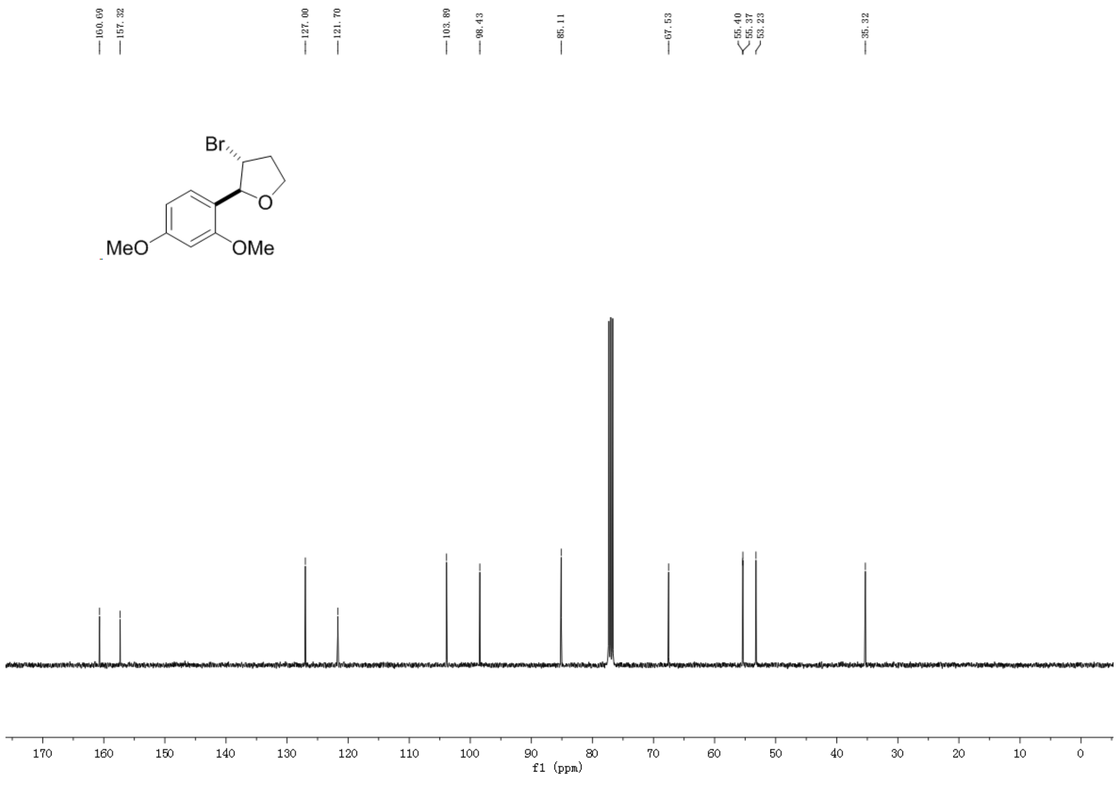
160.69
157.32
127.00
121.70
103.89
98.43
85.11
67.53
55.40
55.37
53.23
35.32
Br
O
MeO
OMe
f1 (ppm)

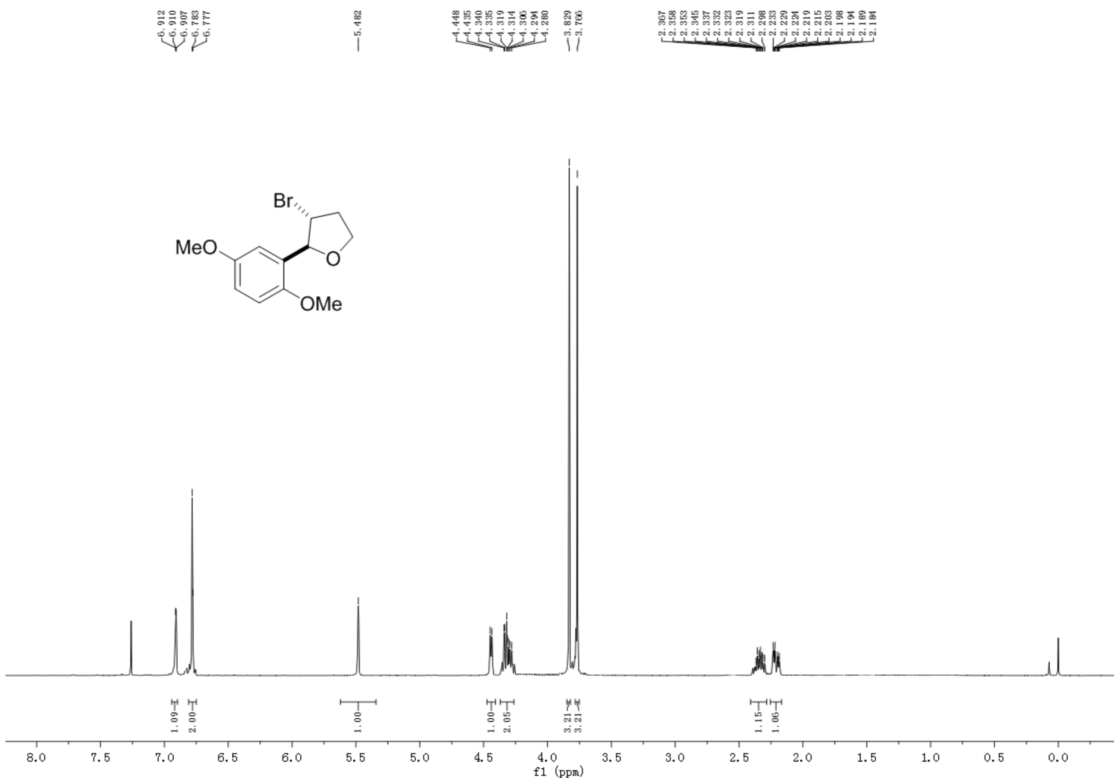

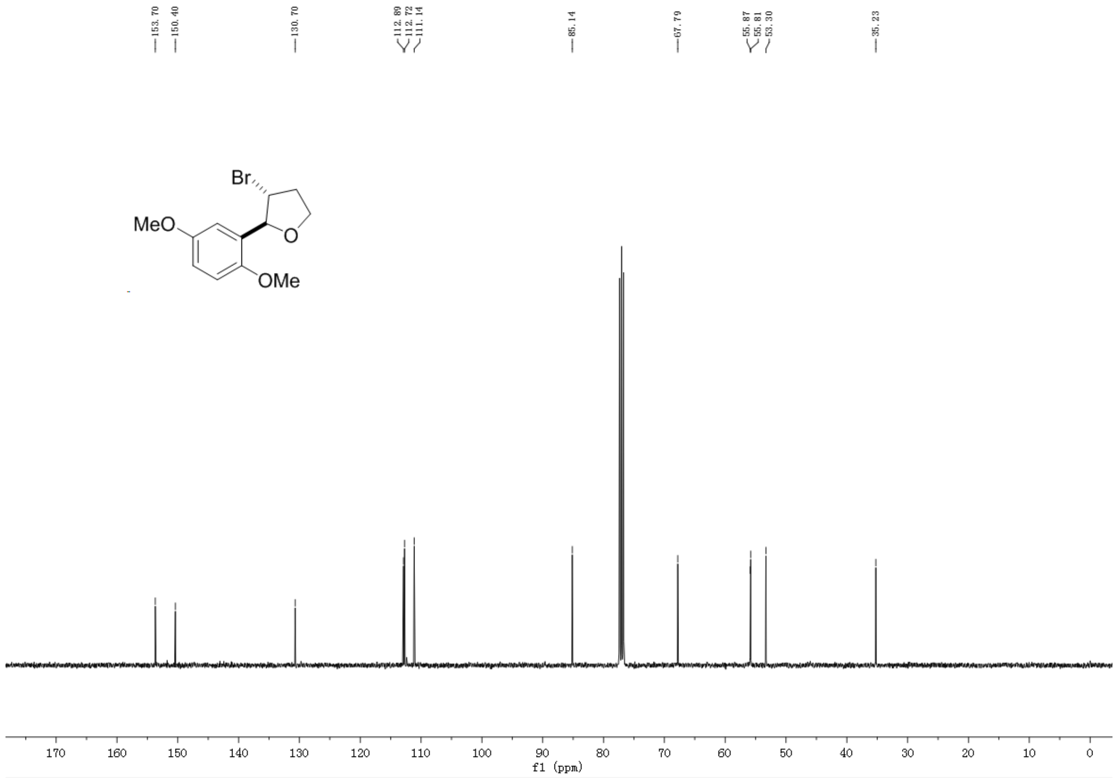

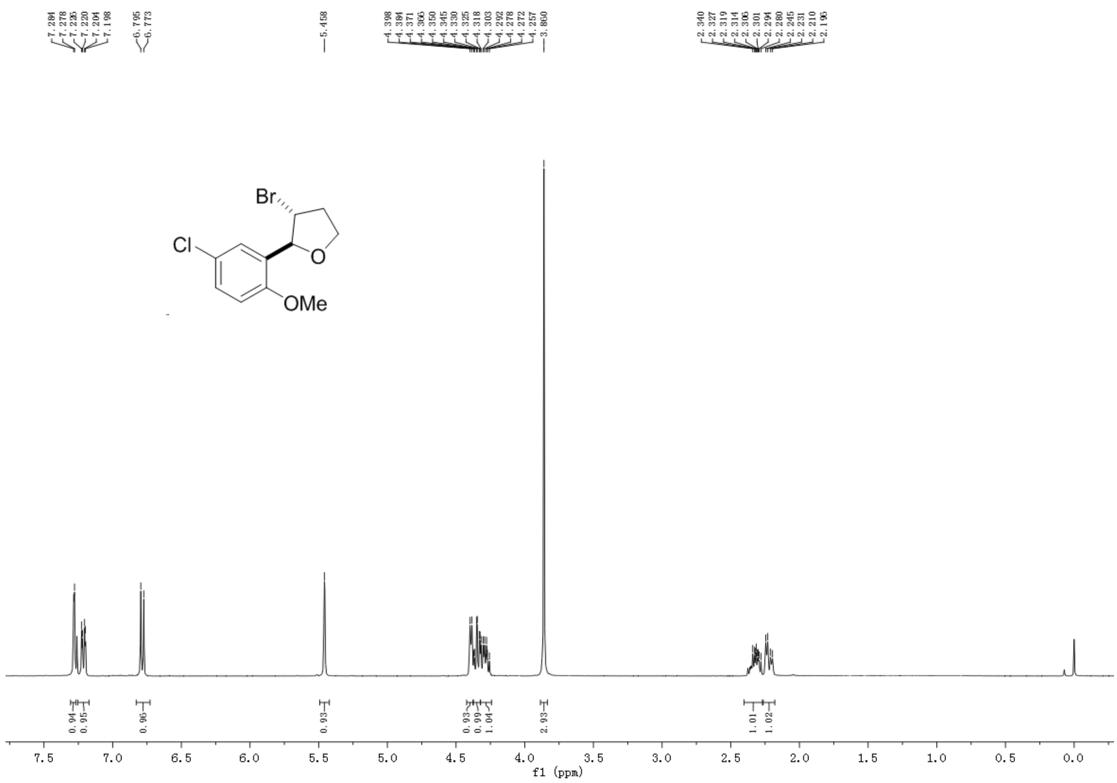
7.284
7.278
7.226
7.220
7.204
7.198
6.795
6.773
5.458
4.398
4.384
4.371
4.356
4.350
4.345
4.330
4.325
4.318
4.303
4.292
4.278
4.272
4.257
3.860
2.340
2.327
2.319
2.314
2.306
2.301
2.294
2.280
2.245
2.231
2.210
2.196
Br
Cl
O
OMe
0.94
0.95
0.96
0.93
0.93
0.99
1.04
2.93
1.01
1.02
f1 (ppm)

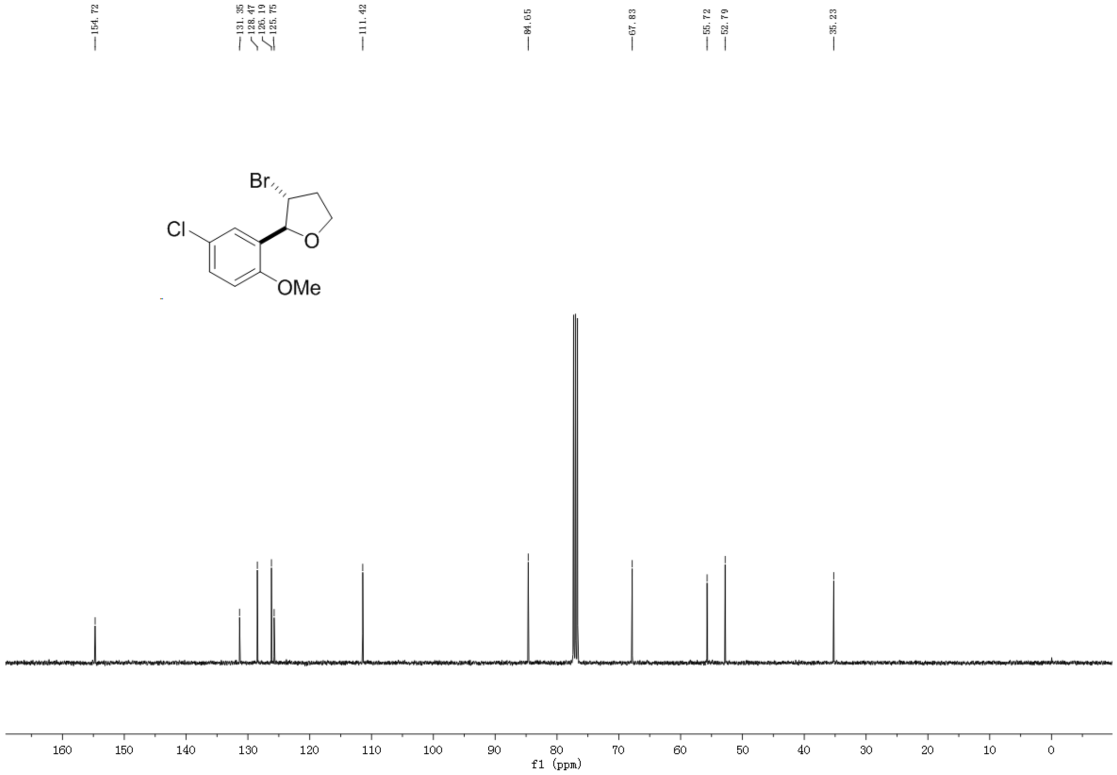
154.72
131.35
128.47
126.19
125.75
111.42
84.65
67.83
55.72
52.79
35.23
Br
Cl
O
OMe
f1 (ppm)

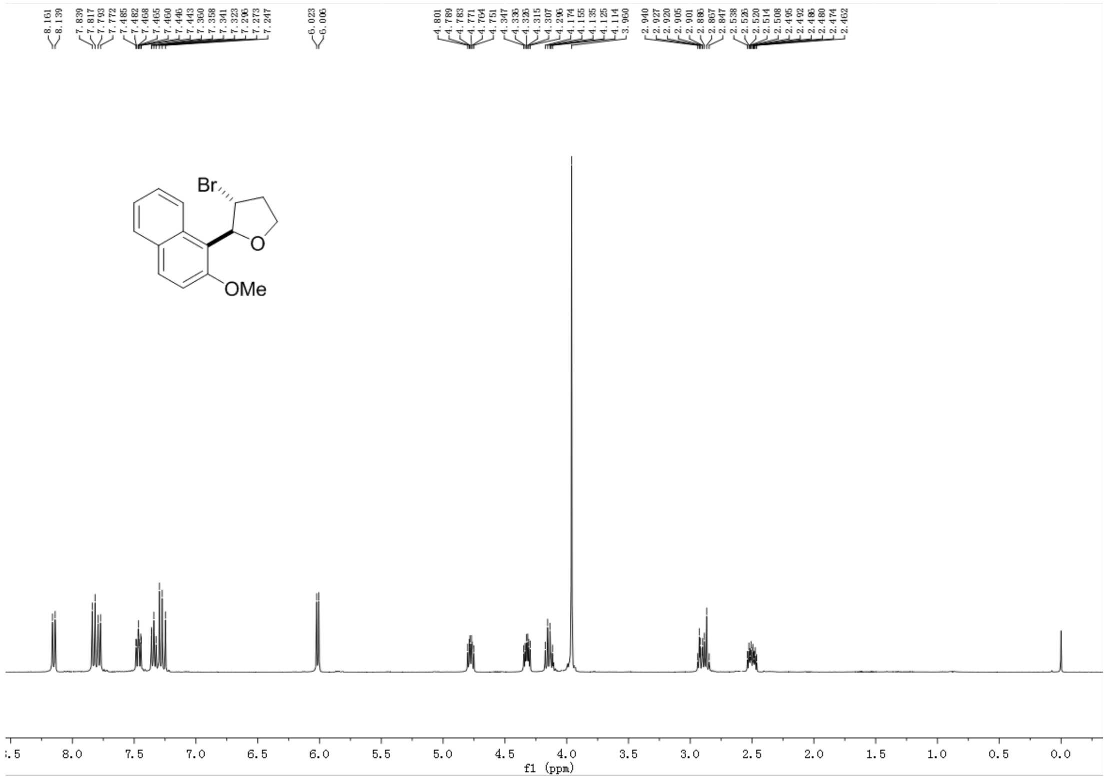

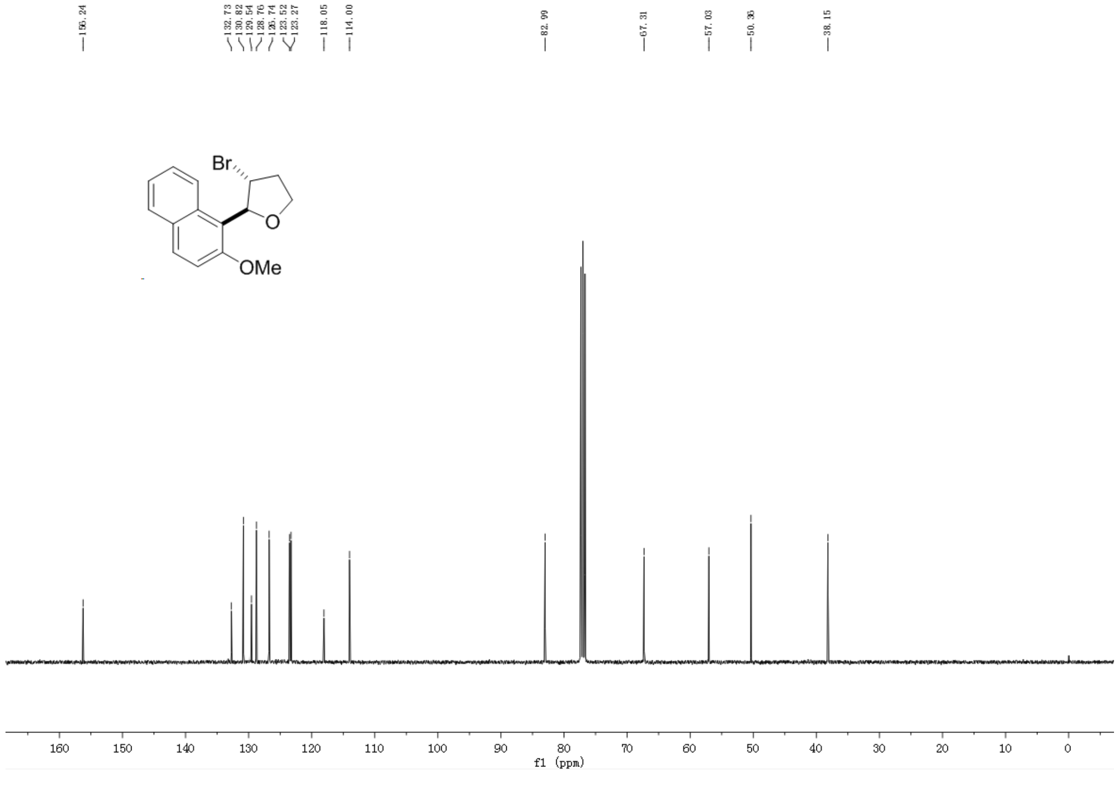

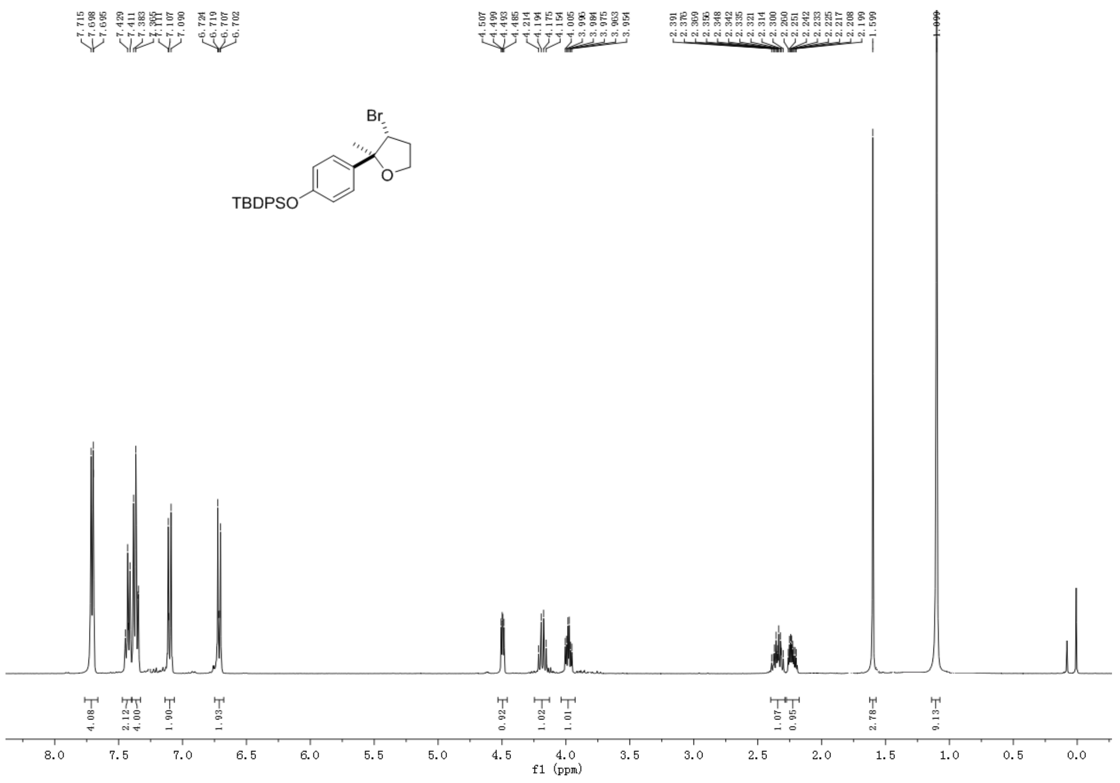

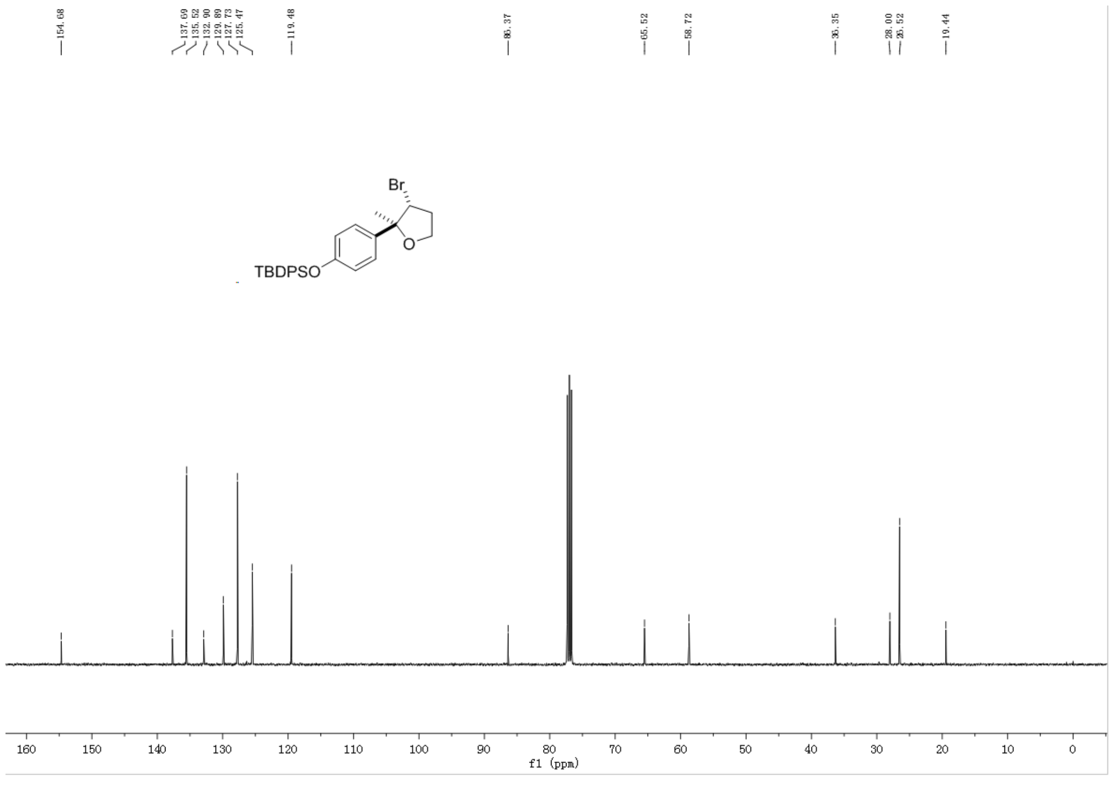

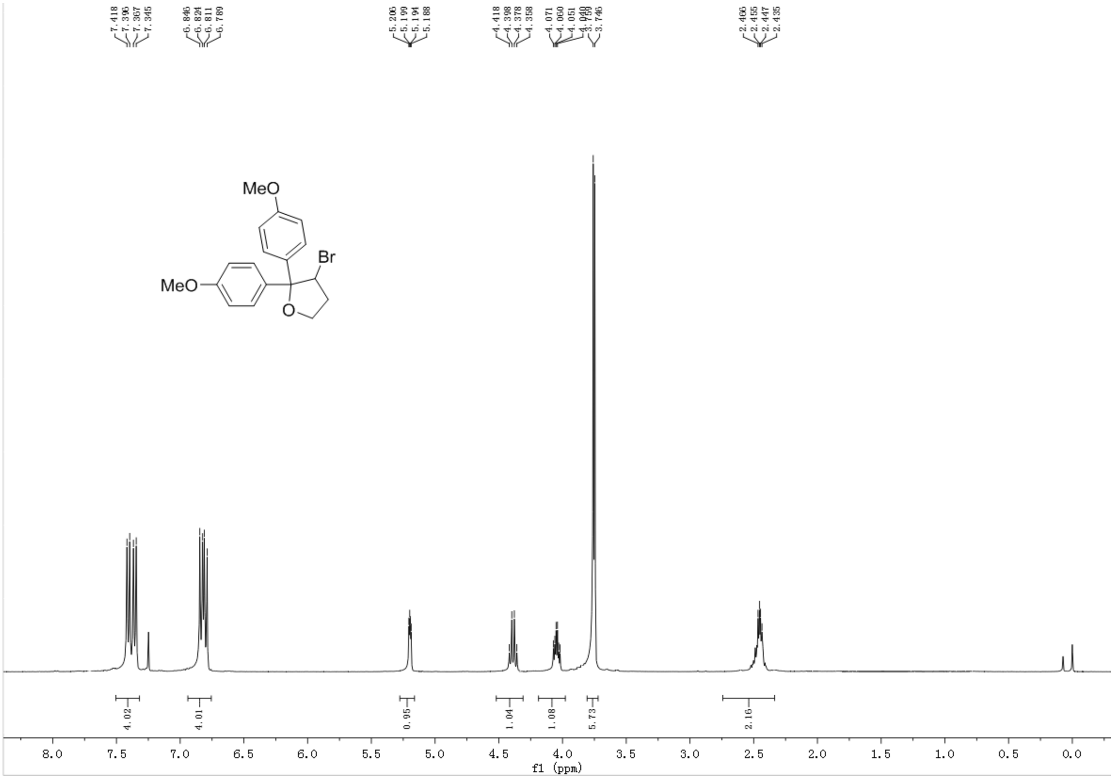

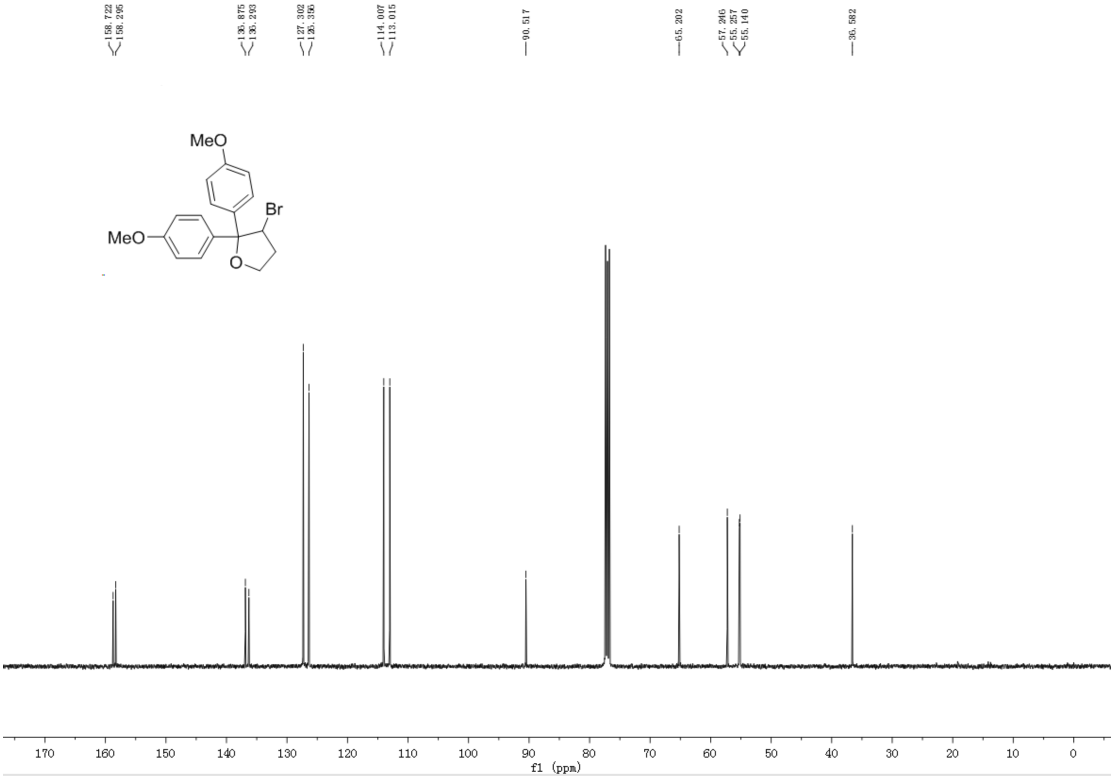

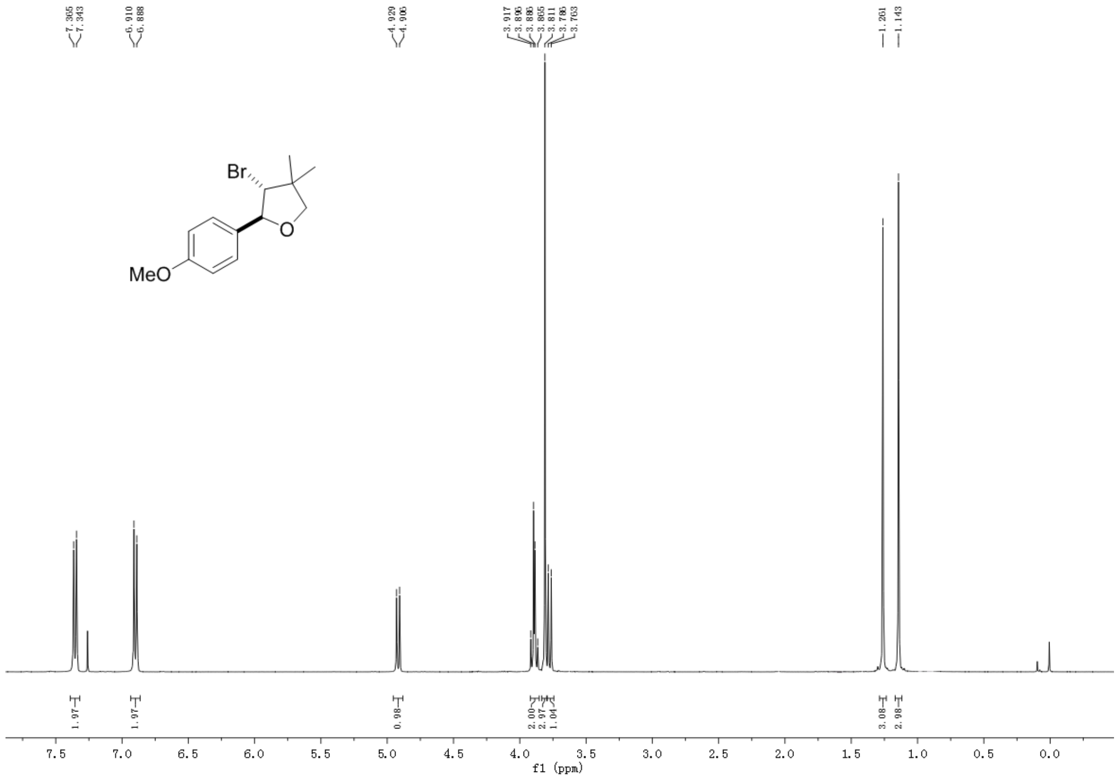
7.365
7.343
6.910
6.888
4.929
4.906
3.917
3.896
3.886
3.865
3.811
3.795
3.763
1.261
1.143
Br
O
MeO
1.97
1.97
0.98
2.00
2.97
1.04
3.08
2.98
f1 (ppm)

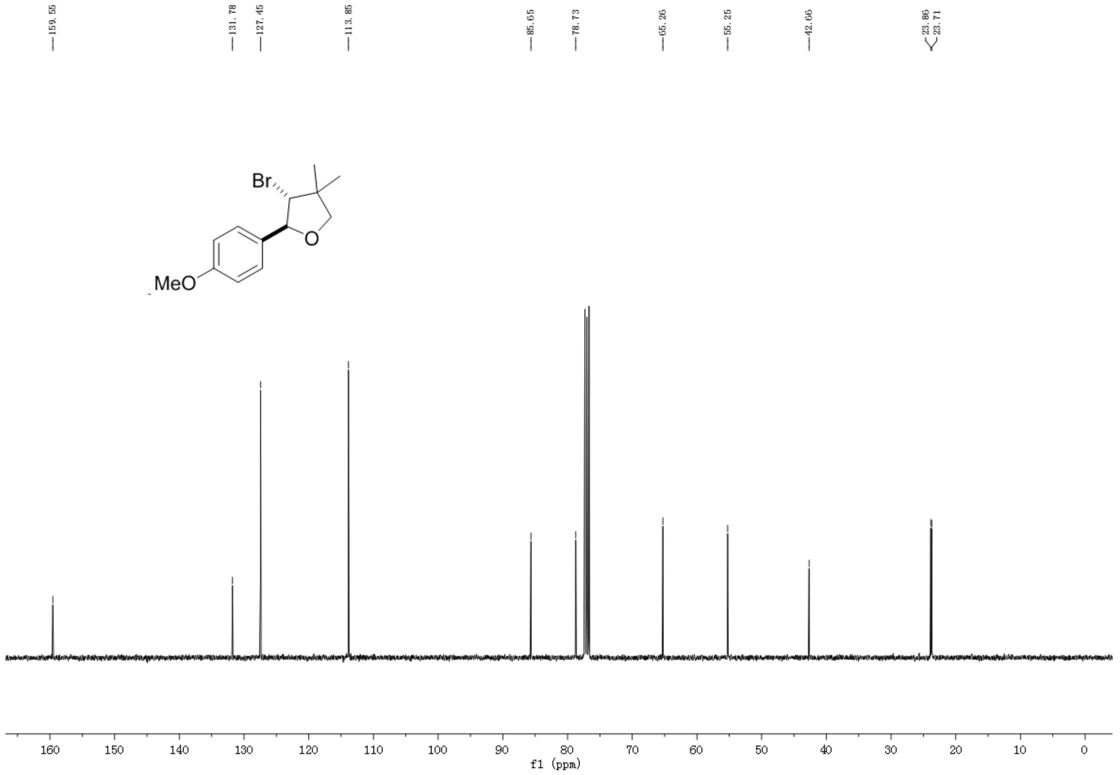
159.55
131.78
127.45
113.85
85.55
78.73
65.26
55.25
42.66
23.86
23.71
Br
O
MeO
f1 (ppm)

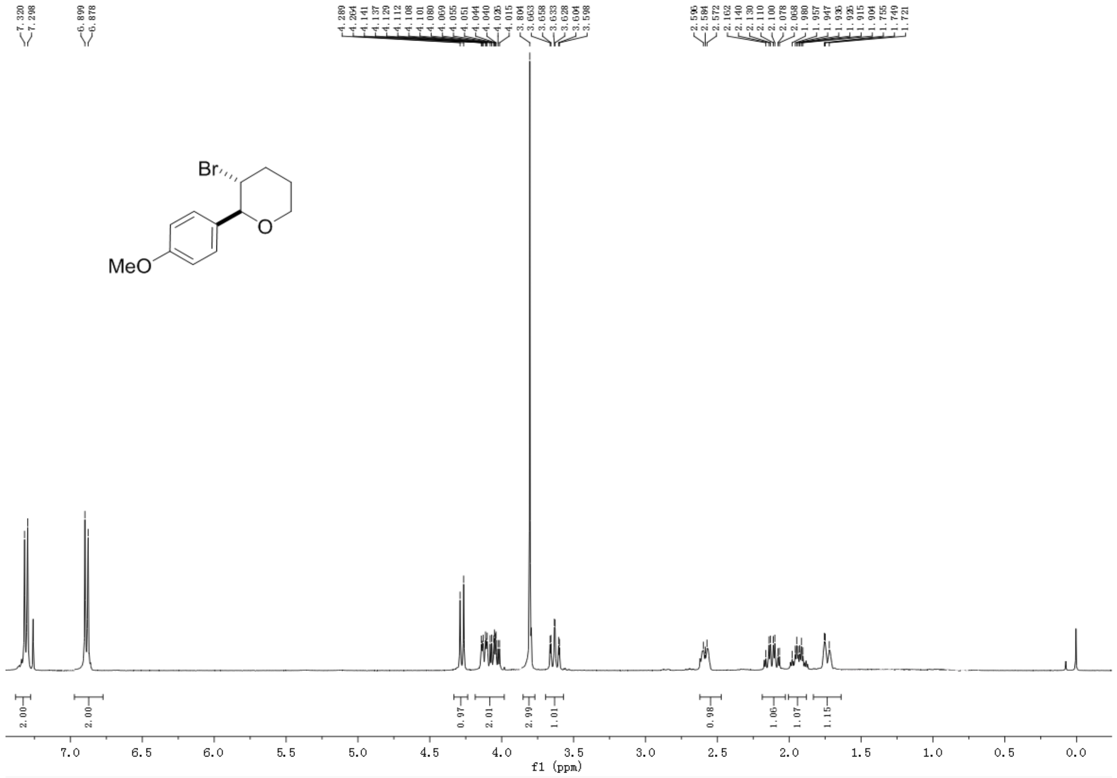
Br
O
MeO
f1 (ppm)

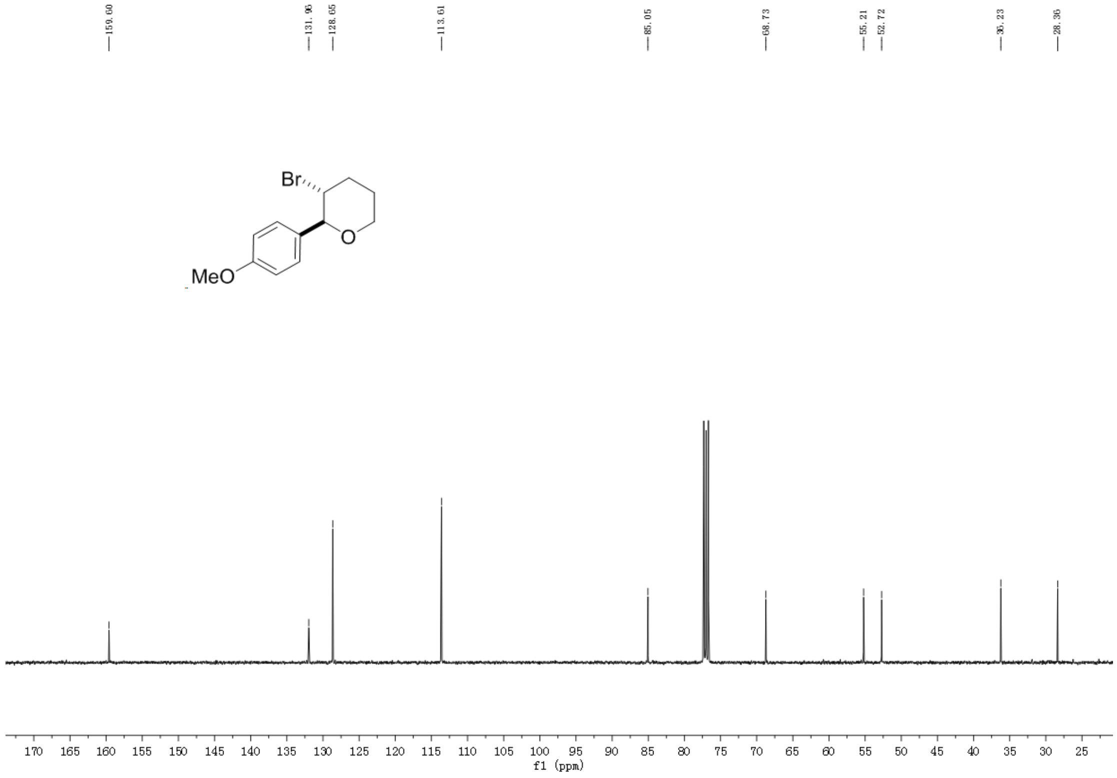
159.60
131.96
128.65
113.61
85.05
68.73
55.21
52.72
36.23
28.36
Br
O
MeO
f1 (ppm)

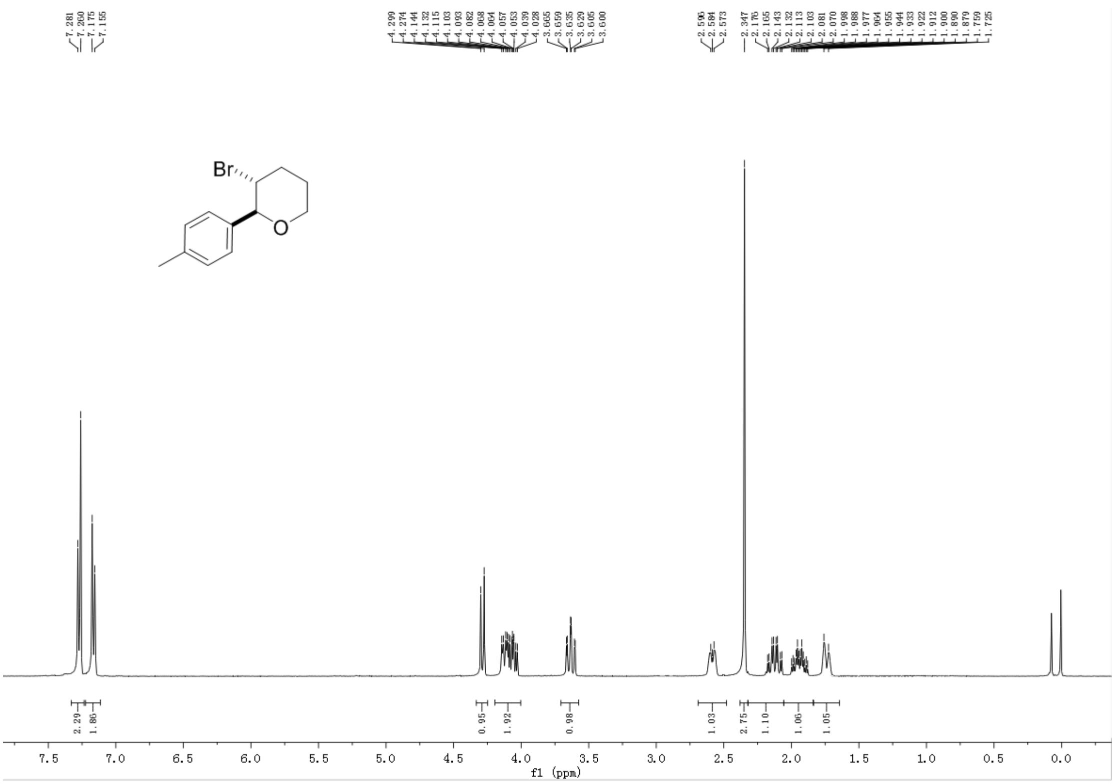
7.281
7.260
7.175
7.155
4.299
4.274
4.144
4.132
4.115
4.103
4.093
4.082
4.068
4.064
4.057
4.053
4.039
4.028
3.665
3.659
3.635
3.629
3.605
3.600
2.596
2.584
2.573
2.347
2.176
2.165
2.143
2.132
2.113
2.103
2.081
2.070
1.998
1.988
1.977
1.964
1.955
1.944
1.933
1.922
1.912
1.900
1.890
1.879
1.759
1.725
Br
O
2.29
1.86
0.95
1.92
0.98
1.03
2.75
1.10
1.06
1.05
f1 (ppm)

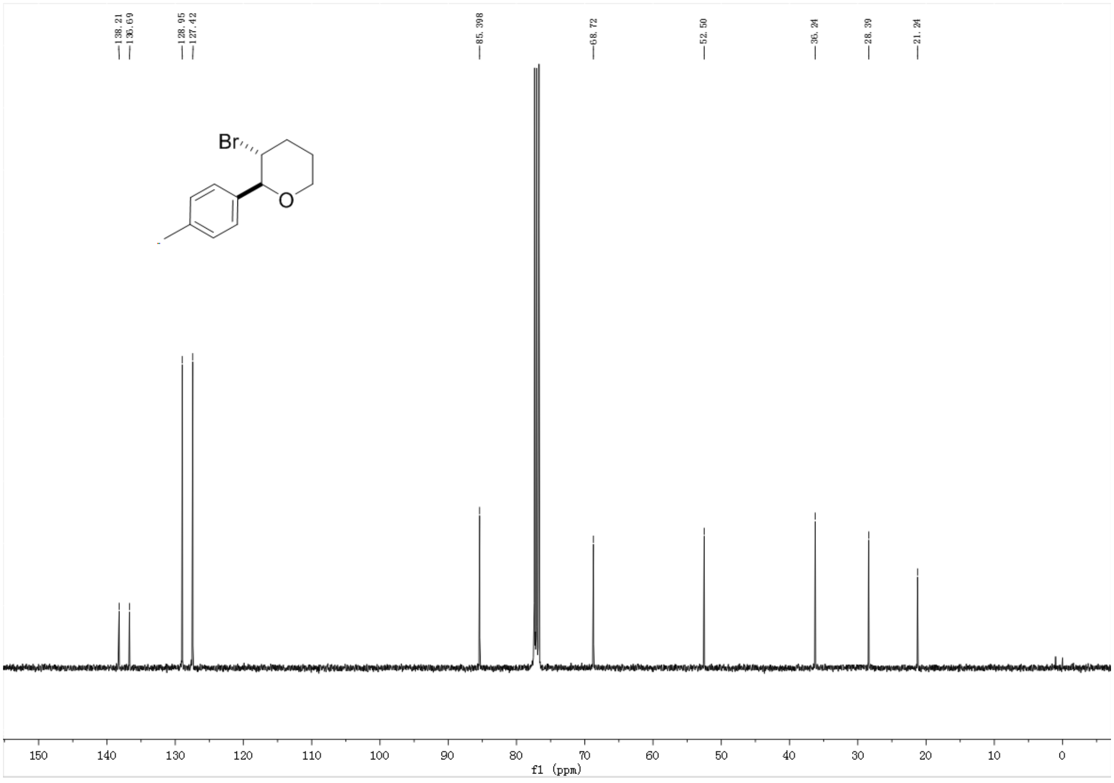
138.21
136.69
128.95
127.42
85.398
68.72
52.50
36.24
28.39
21.24
Br
O
f1 (ppm)

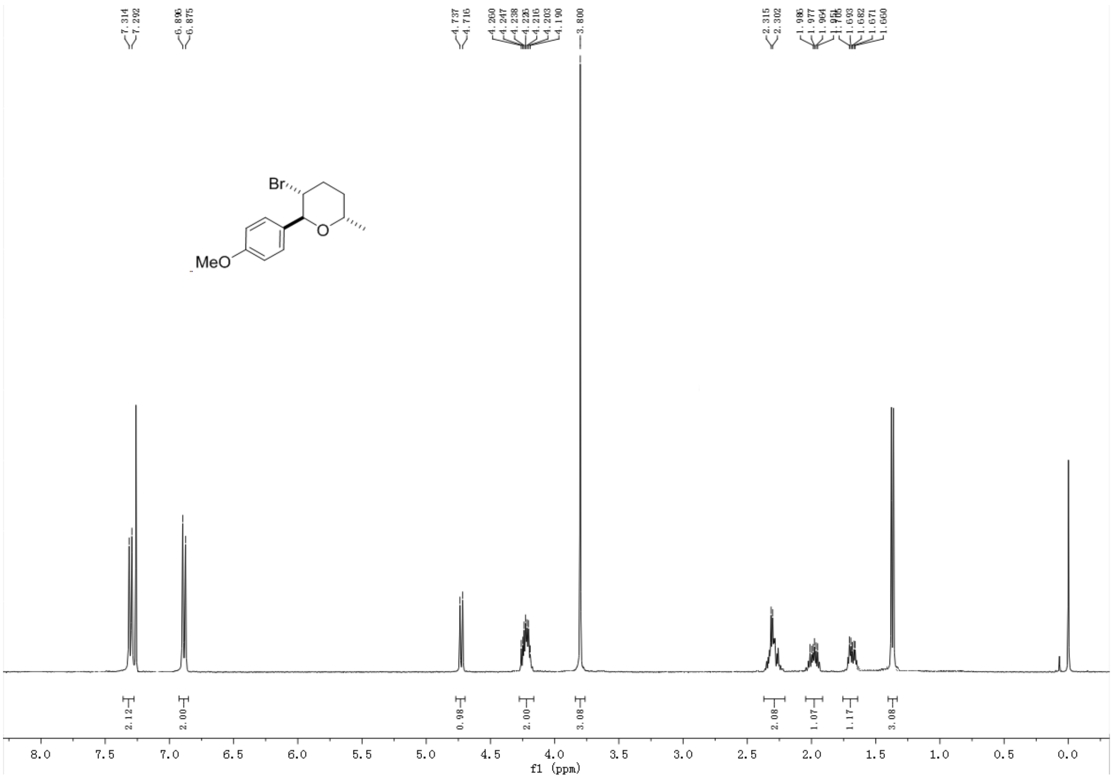
Br
MeO
O
f1 (ppm)

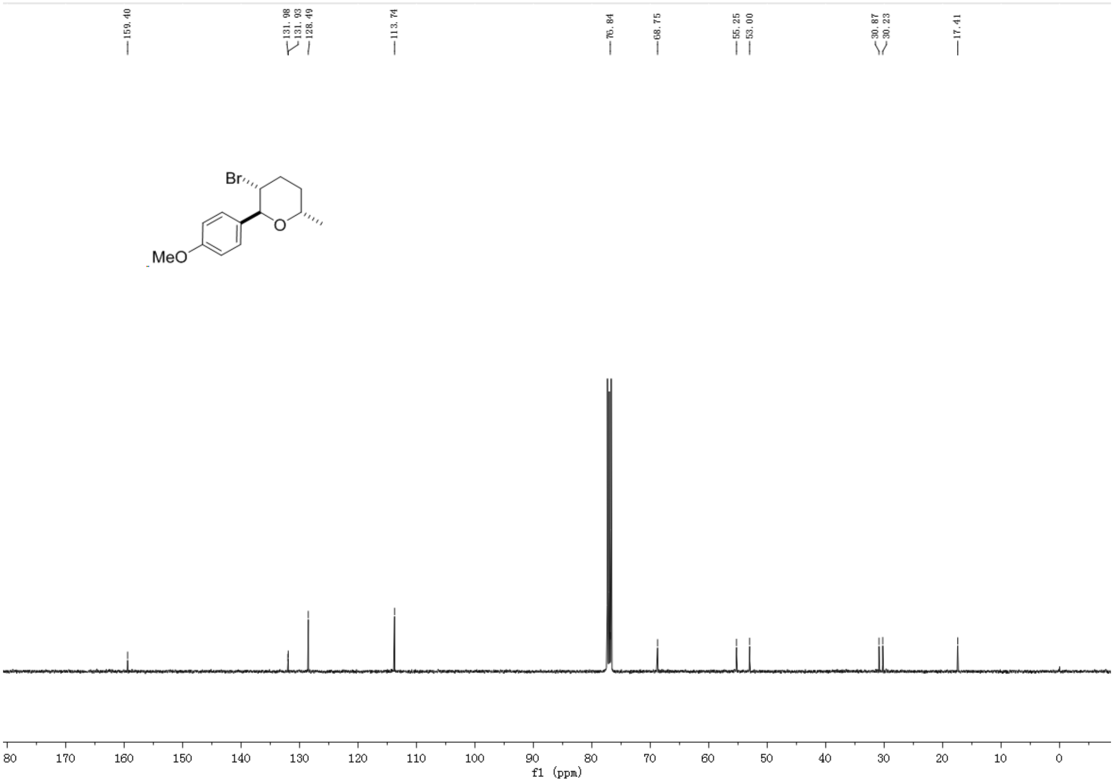
Br
MeO
O
f1 (ppm)

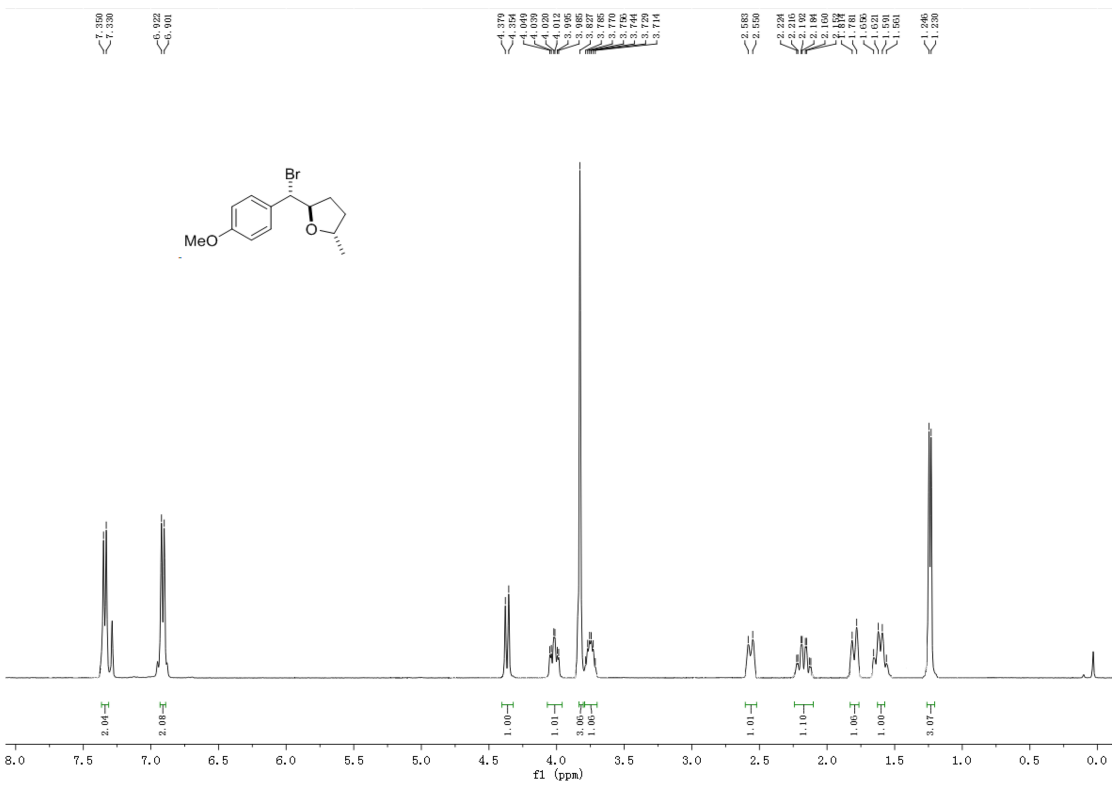

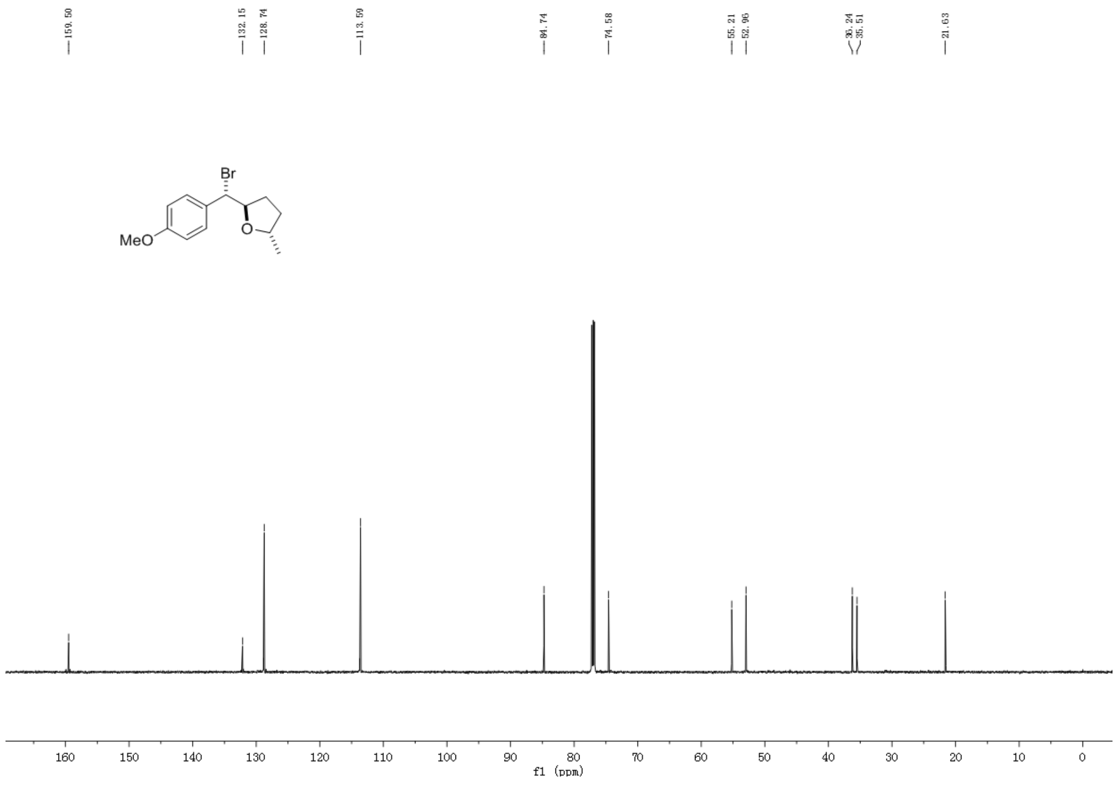

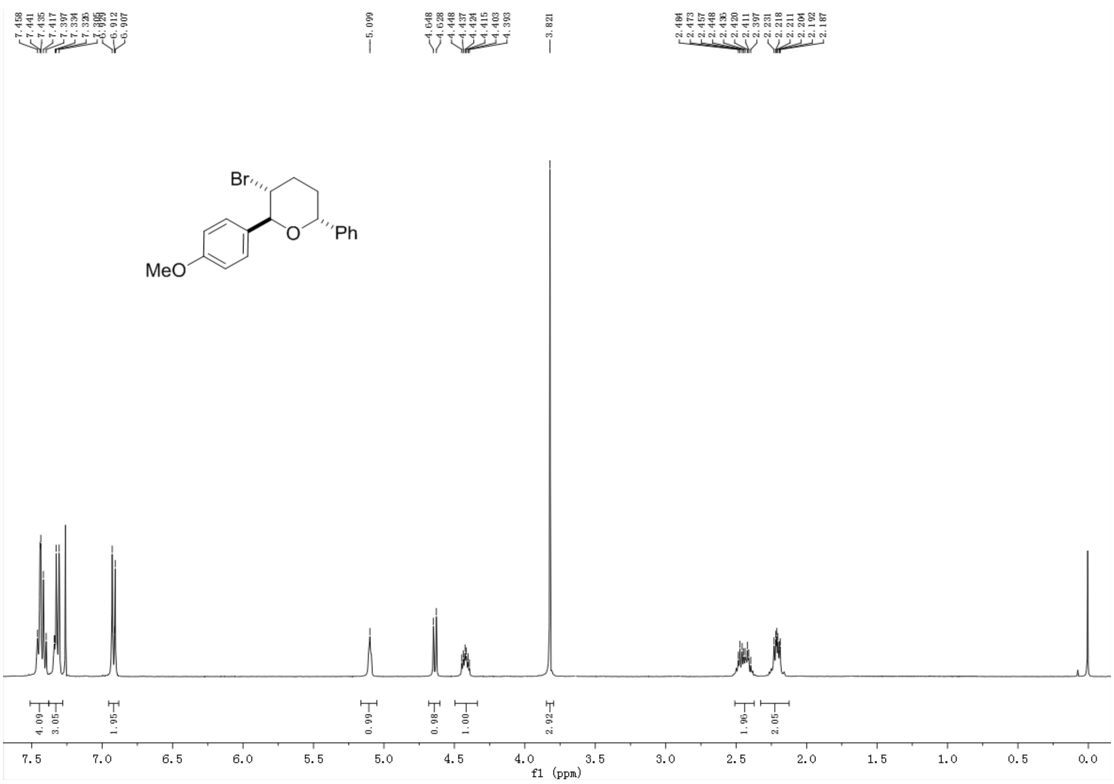
Br
O
Ph
MeO
f1 (ppm)

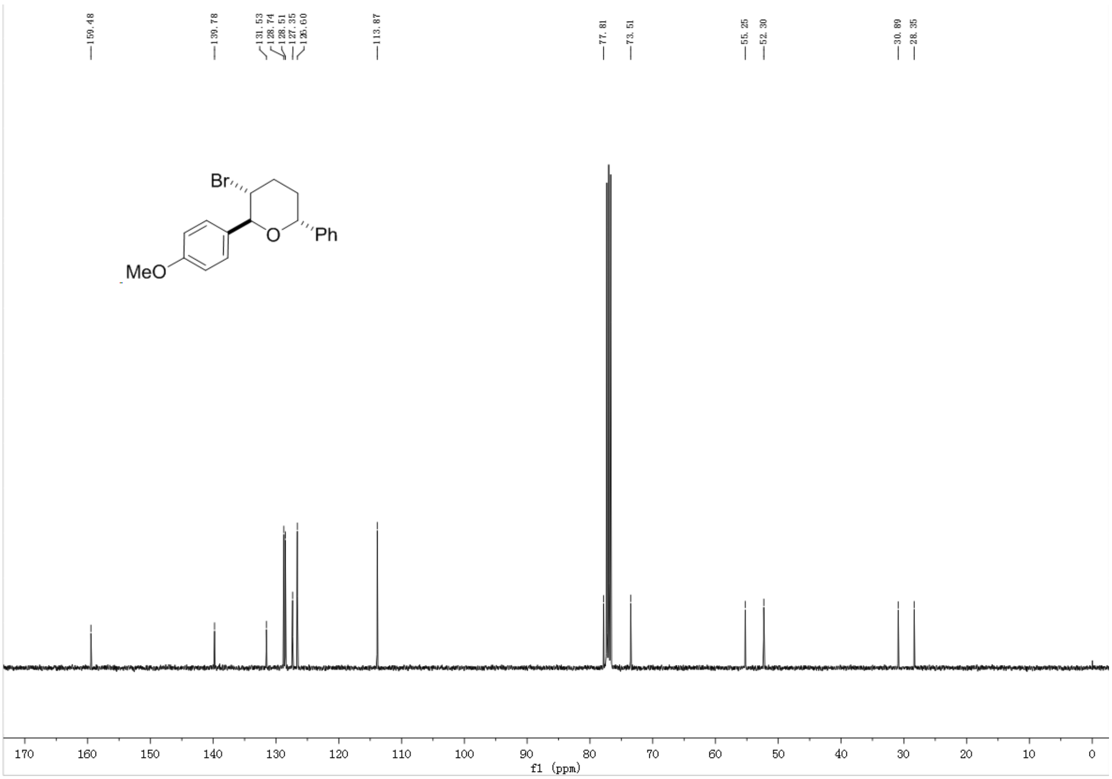
Br
O
Ph
MeO
f1 (ppm)

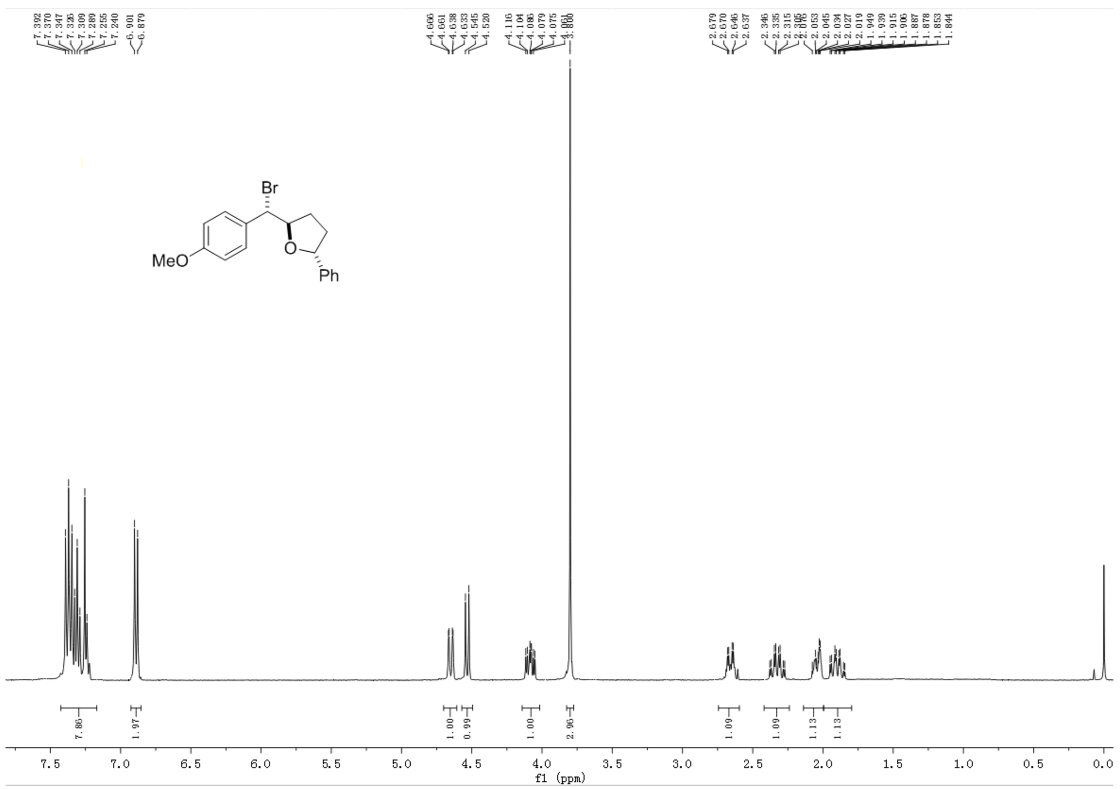

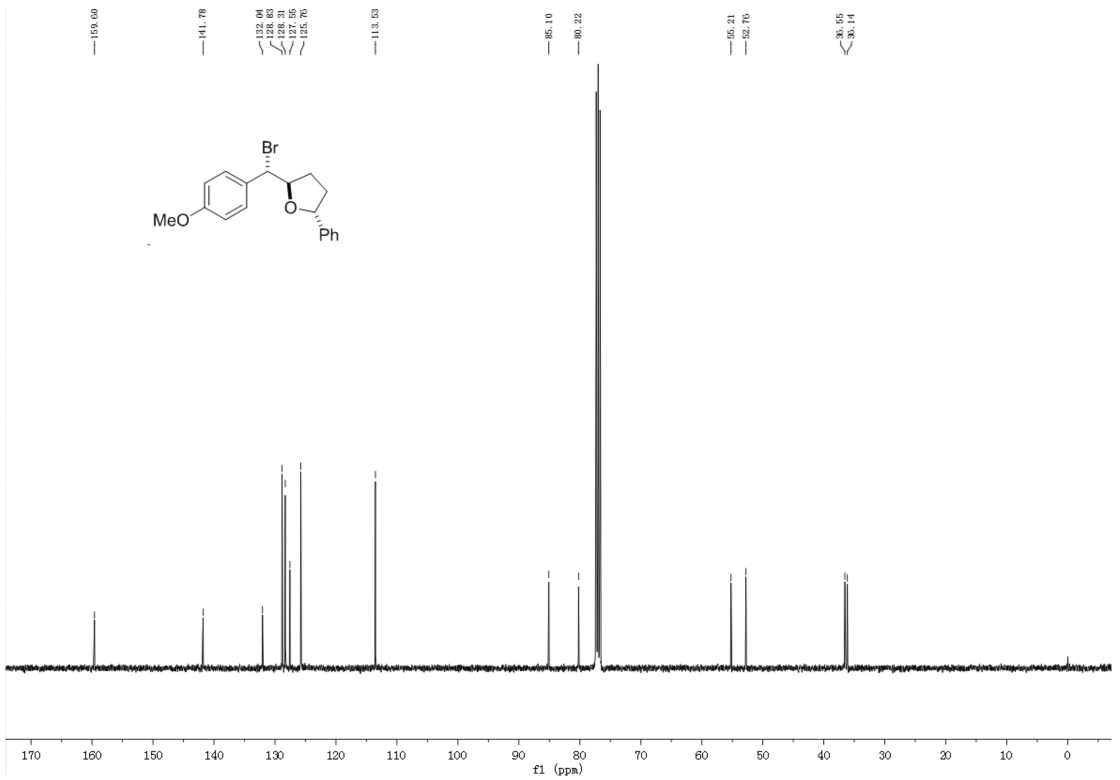

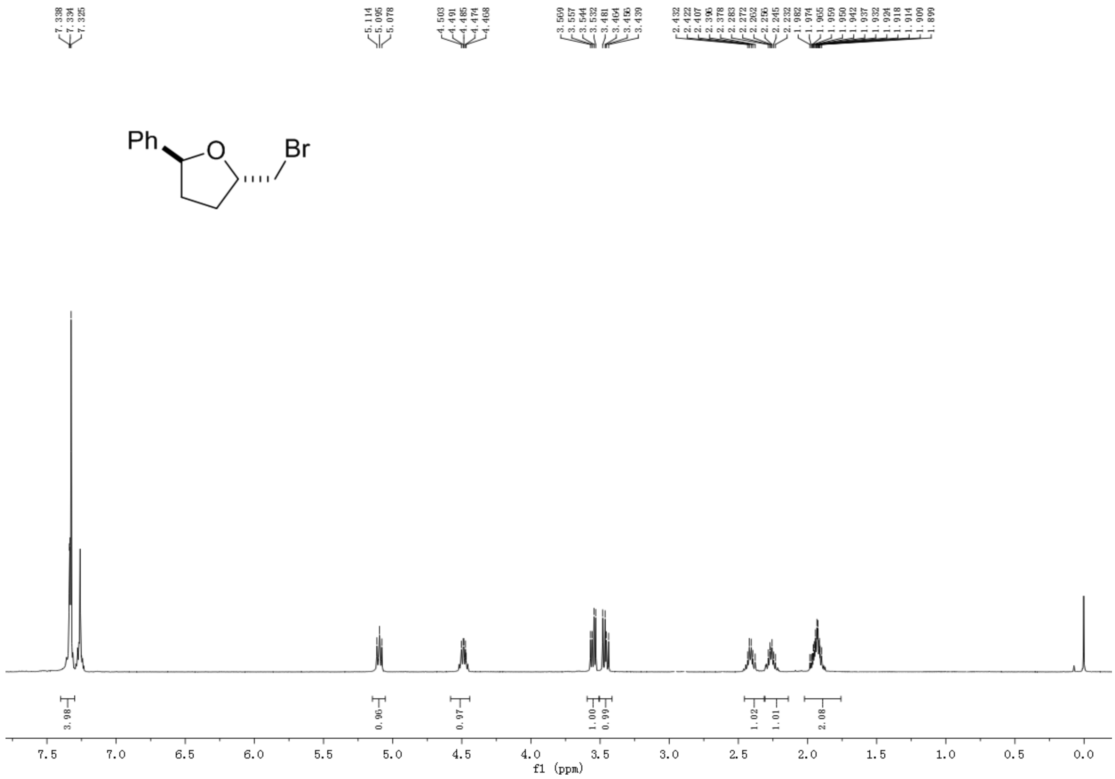
Ph
O
Br
7.5
7.0
6.5
6.0
5.5
5.0
4.5
4.0
3.5
3.0
2.5
2.0
1.5
1.0
0.5
0.0
f1 (ppm)

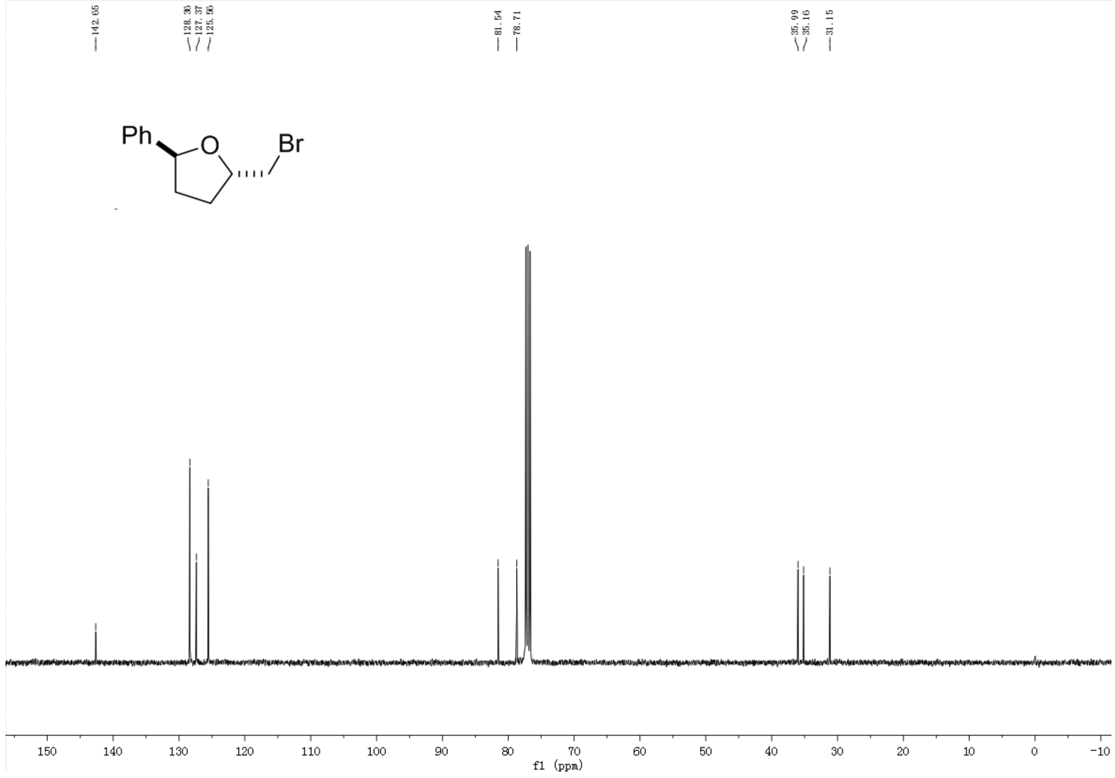
142.65
128.36
127.37
125.56
81.54
78.71
35.99
35.16
31.15
Ph
O
Br
150
140
130
120
110
100
90
80
70
60
50
40
30
20
10
0
-10
f1 (ppm)
